# New Organotin (IV) Compounds Derived from Dehydroacetic Acid and Thiosemicarbazides: Synthesis, Rational Design, Cytotoxic Evaluation, and Molecular Docking Simulation

**DOI:** 10.1155/2023/7901843

**Published:** 2023-10-25

**Authors:** Elizabeth Gómez, José Miguel Galván-Hidalgo, Guillermo Pérez-Cuéllar, Karoline Alondra Huerta-Landa, Arturo González-Hernández, Omar Gómez-García, Dulce Andrade-Pavón, Teresa Ramírez-Apan, Karla Daniela Rodríguez Hernández, Simón Hernández, Patricia Cano-Sánchez, Homero Gómez-Velasco

**Affiliations:** ^1^Instituto de Química, Universidad Nacional Autónoma de México, Circuito Exterior S/N, Ciudad Universitaria, Alcaldía Coyoacán, C. P. 04510, Ciudad de México, Mexico; ^2^Escuela Nacional de Ciencias Biológicas, Instituto Politécnico Nacional, Prolongación de Carpio y Plan de Ayala S/N, Colonia Santo Tomás 11340, Ciudad de México, Mexico; ^3^Departamento Microbiología, Escuela Nacional de Ciencias Biológicas, Instituto Politécnico Nacional, Prolongación de Carpio y Plan de Ayala S/N, Colonia Santo Tomás 11340, Ciudad de México, Mexico; ^4^Departamento Fisiología, Escuela Nacional de Ciencias Biológicas, Instituto Politécnico Nacional, Av. Wilfrido Massieu 399, Colonia Nueva Industrial Vallejo 07738, Ciudad de México, Mexico

## Abstract

Organotin complexes were prepared through a one-pot reaction with three components by reacting thiosemicarbazide or 4-methyl-3-thiosemicarbazide or 4-phenylthiosemicarbazide, dehydroacetic acid (DHA) and dibutyl, diphenyl, dicyclohexyl, and bis[(trimethylsilyl)methyl]tin(IV) oxides; all complexes were characterized by infrared (IR), ultraviolet-visible (UV-vis), mass spectrometry (MS), and nuclear magnetic resonance (NMR) spectroscopy. The ^119^Sn NMR revealed chemical shifts corresponding to a pentacoordinated environment in solution. The X-ray crystallography of the two complexes evidenced the formation of monomeric complexes with a pentacoordinated geometry around tin via three donor atoms from the ligand, the sulfur of the thiol, the nitrogen of the imine group, and the oxygen of the pyran ring. The geometries of the five-coordinated complexes **3a** (Bu_2_SnL3), **3c** (Ph_2_SnL3), and **3d** (Cy_2_SnL3) acid were intermediate between square pyramidal and trigonal bipyramidal, and complex **1a** (Bu_2_SnL1) adopted a bipyramidal trigonal geometry (BPT). The sulforhodamine B assay assessed the cytotoxicity of organotin(IV) complexes against the MDA-MB-231 and MCF-7 (human mammary adenocarcinoma) cell lines and one normal COS-7 (African green monkey kidney fibroblast). The IC_50_ values evidenced a significant antiproliferative effect on cancer cells; the complexes were more potent than the positive cisplatin control and the corresponding ligands, dehydroacetic acid thiosemicarbazone (**L1**), dehydroacetic acid-N(4)-methylthiosemicarbazone (**L2**), and dehydroacetic acid-N(4)-phenylthiosemicarbazone (**L3**). The IC_50_ values also indicated that the organotin(IV) complexes were more cytotoxic against the triple-negative breast cell line MDA-MB-231 than MCF-7, inducing significant morphological alterations. The interactions of organotin(IV) **1c** (Ph_2_SnL1), **1d** (Cy_2_SnL1), and **1e** (((CH_3_)_3_SiCH_2_)_2_SnL1) were evaluated with ss-DNA by fluorescence; intensity changes of the fluorescence were indicative of the displacement of ethidium bromide (EB), confirming the interaction of the organotin(IV) complexes with ss-DNA; the results showed a DNA binding affinity. The thermodynamic parameters obtained through isothermal titration calorimetry showed that the interaction of **1c** (Ph_2_SnL1), with ss-ADN, was exothermic. Molecular docking studies also demonstrated that the organotin(IV) complexes were intercalated in DNA by conventional hydrogen bonds, carbon-hydrogen bonds, and *π*-alkyl interactions. These complexes furthermore showed a greater affinity towards DNA than cisplatin.

## 1. Introduction

Thiosemicarbazones and semicarbazones are privileged ligands whose chelating properties have been widely studied due to the presence of different donor atoms such as nitrogen, sulfur, and oxygen; their soft and hard donor characters allow the formation of a variety of complexes in the presence of metallic ions with different coordination geometries [1, 2]. These types of compounds also possess interesting biological properties, including antiviral, antibacterial, antioxidant, antifungal, antimalarial, antileprotic, and anticancer ones [3–5]; their use in the treatment of Alzheimer's disease has also been studied [6], and in many cases, they act as primary pharmacophores [7]. The functionalization of N4-substituted thiosemicarbazones has been demonstrated to represent a valuable strategy, for improving their anticancer and antibacterial activities [8]. Thiosemicarbazones have been the focus of special attention due to their proven efficacy against cancers such as leukemia, breast, pancreas, cervical, prostate, bladder, and lung tumors. The structure-activity relationship of thiosemicarbazones indicates that the antineoplastic activity is significant if heterocyclic nitrogen in the *α*-position to thiosemicarbazone and a conjugated N, N, S donor system is present in the molecule. Moreover, the modification of the heteroaromatic ring produces better activity; although some compounds have entered phase I trials, their use has so far been avoided due to their associated gastrointestinal and hematological side effects [9]. Thiosemicarbazones are well known for their remarkable coordination, flexibility, and chelating ability to form complexes with different metals, and their pharmacological response depends on the metal and ligand properties. However, the ligand is a determinant in targeting specific sites in the cancer cells [10, 11].

3-Acetyl-4-hydroxy-6-methyl-2-oxo-2*H*-pyran, also known as dehydroacetic acid (DHA), is widely used because of its different reactive sites allow it to react with electrophiles and nucleophiles, giving rise to the formation of various heterocyclic compounds. Consequently, several synthetic strategies have been reported for the transformation of DHA into pharmacologically active compounds such as pyrazole, pyrimidines, pyrones, pyrano-pyrazole, and oxazinthiones, which exhibit antimicrobial, antibacterial, antifungal, anti-inflammatory, and anti-HIV activities [12]. DHA has also been included in the preparation of both symmetrical and unsymmetrical Schiff-based ligands using classical and microwave-green chemical approaches; these ligands act as excellent chelating agents in the presence of oxo-metal ions, copper(II) and nickel(II), and organosilicon(IV) [13, 14]. Schiff-based ligands have been widely explored due to their versatility, synthesis, feasibility, and ability to act as complexing ligands with metallic ions, with applications in diverse areas of research such as catalysis, medicinal, and pharmaceutical research, in the preparation of novel materials, organic solar cells, metal-organic frameworks, and more recently as fluorescent chemosensors [15–21].

Tin(IV) complexes are known to display unique biological and medicinal properties; although the cytotoxic and anticancer potential of diverse classes of organotin compounds has been widely proven *in vitro*, fewer *in vivo* studies have been described [22–24]. Their biological response has been associated with the organic moieties bonded to the metal, the oxidation state of the metal, and their coordination geometry. However, the nature and lipophilicity of the ligand are involved in transporting and targeting specific sites [25, 26]. The therapeutical properties of organotin compounds have been explored with multiple ligands; among these, thiosemicarbazones have been found to possess interesting antifungal and antibacterial activities [27–29].

Despite the number of reports that have evidenced the highly cytotoxic effect of organotin complexes with a plethora of ligands against cancer cell lines, DHA has been scarcely studied. Considering that both the metal nature and the ligand properties affect its therapeutical response, particularly the substitution on the nitrogen of the thiosemicarbazone and the substituent attached to the tin atom, in this study, a series of organotin(IV) Schiff-based complexes were designed and synthesized using biological organic ligands to assess their cytotoxic activity in vitro. The complexes were fully characterized and tested against cancer cell lines MCF-7, MDA-MB-231 (human mammary adenocarcinoma), and the normal cell line COS-7 (African green monkey kidney fibroblast). The results revealed that the complexes had a greater inhibitory effect on the cells compared to the positive control Cisplatin.

## 2. Materials and Methods

### 2.1. Materials

The reagents and solvents were purchased from commercial suppliers and used without additional purification; dicyclohexyl and bis[(trimethylsilyl)methyl]tin oxide were prepared as described in the literature [30].

### 2.2. Physical Measurements

Melting points were measured on a Fisher-Johns MEL-TEMP II apparatus. Infrared (IR) attenuated total reflectance (ATR) spectra were recorded on a Thermo Scientific instrument over a range from 200 to 4000 cm^−1^. Molar conductivity measurements were carried out on a Hanna 6484 instrument using anhydrous methanol as a solvent. The ultraviolet-visible (UV-Vis) absorption spectra were obtained on a Cary 100 series Agilent UV-Vis spectrophotometer, using a methanol solution at a concentration of 2.04510 × 10^−5^ M. The ^1^H, ^13^C, and ^119^Sn spectra were recorded on Bruker Avance 300 and 400 MHz spectrometers and analyzed using the Mestre Nova software. Two-dimensional (2D) nuclear magnetic resonance (NMR) experiments (COSY (correlated spectroscopy), DEPT-135 (distortionless enhancement by polarization transfer), HSQC (heteronuclear single quantum coherence), and HMBC (heteronuclear multiple bond correlation)) were conducted for a complete assignment of the signals. The DART+ (direct analysis in real time) mass spectra were acquired using a JEOL-JMS-X103 spectrometer. The CLogP values were computed via the Molinspiration Cheminformatics free web services at https://www.molinspiration.com in Slovensky Grob, Slovakia.

A Bruker Smart Apex CCD diffractometer with a graphite monochromator (*λ*_(Mo-K_*α*_)_ = 0.71073 A) at *T* = 150 K was used to collect diffraction intensity data for the **1a**, **3a**, **3c**, and **3d** compounds. The structures were solved by direct methods and refined by full-matrix least-squares on *F*^2^. The refinement of the structures was carried out using the SHELXL software. All nonhydrogen atoms were refined anisotropically, and the hydrogen atoms were located at idealized positions. Crystallographic data for structures reported in this manuscript have been deposited with the Cambridge Crystallographic Data Centre under the CCDC numbers: 2241000 (complex **3c**), 2241001 (complex **3d**), 2241002 (complex **1a**), and 2241003 (complex **3a**). Copies of these data can be obtained free of charge from https://ccdc.cam.ac.uk/data_request/cif.

### 2.3. Culture of Cell Lines

The compounds were screened in vitro against two cancer cell lines, MCF-7 and MDA-MB-231 (human mammary adenocarcinoma), and the normal cell line Cos-7 (African green monkey kidney fibroblast). The MCF-7 and MDA-MB231 cell lines were cultured in RPMI-1640 medium, and Cos-7 was cultured in DMEM (Dulbecco's Modified Eagle's Medium) and supplemented with 10% of fetal bovine serum, 2 mM L-glutamine, 10000 units/ml penicillin G sodium, 10000 *μ*g/ml streptomycin sulfate, 25 *μ*g amphotericin B (Gibco), and 1% of nonessential amino acids (Gibco). The cell lines were maintained at 37°C in a humidified atmosphere with 5% CO_2._ The viability of the cells used in the experiments exceeded 95%, as determined with trypan blue.

### 2.4. Cytotoxicity Assay

The human tumor cytotoxicity was assessed using the protein-binding dye sulforhodamine B (SRB) in a microculture assay to measure the cell growth as described by [31]. The human tumors and normal cells were treated with the complexes. The cells were removed from the tissue culture flask by treatment with trypsin and diluted with fresh media from the cell suspension to 100 *μ*l containing 5000–10000 cells per well. The cells were then pipetted into 96 microliter plates (Costar) and incubated at 37°C for 24 h in a 5% CO_2_ atmosphere. Subsequently, 100 *μ*l of a solution of the corresponding organotin complex at different concentrations was added to each well. The cultures were exposed to the compounds for 48 h. After the incubation period, the cells were fixed to the plastic substratum by the addition of 50 *μ*l of cold trichloroacetic acid (50% aqueous).

The plates were incubated at 4°C for one hour, washed with tap water, and air-dried. The addition of 4% SRB stained trichloroacetic acid-fixed cells. The free SRB was removed by washing with 1% aqueous acetic acid, the plates were air-dried, and the dyed was solubilized by adding ten mM unbuffered Tris base (100 *μ*l). The plates were placed on a shaker for 10 min, and the absorption was measured at 515 nm using an ELISA plate reader (Bio-Tex Instruments).

### 2.5. Morphological Changes Evaluation (Light Microscopy)

To evaluate the morphological alterations induced by organotin(IV) complexes, MCF-7 and MDA-MB231 cell lines were cultured on a tissue culture flask with or without different treatments for 48 h. After incubation, cells were washed two times with PBS, fixed with methanol 100%, and stained with Wright-Giemsa (Sigma-Aldrich, WG32-1L). The culture flasks were examined under an Olympus IX71 Inverted Fluorescence Phase Contrast Microscope. The images were recorded with a Nikon Coolpix 4300 digital camera using the 20x objectives and analyzed by ImageJ software [32].

### 2.6. Stability of the Complexes in Solution

The stability of the complexes in biological media was tested to evaluate their cytotoxicity. The compounds were dissolved in a DMSO-Tris-HCl buffer (3 : 7 v/v) over a 72 h period, and the UV-Vis spectra were recorded at 0 and 72 h. ^1^H NMR spectra in DMSO-*d6* of complexes **1a**, **1d**, **2a**, **2d**, **3a**, and **3d** were also acquired at 0, 24, 56, and 72 h.

### 2.7. Isothermal Titration Calorimetry (ITC)

The ITC experiment was performed on a MicroCal^TM^ iTC 200 System, manufactured by GE Healthcare, Northampton, MA, USA. The binding assay of ss-DNA to organotin(IV) complex (**1c**) was measured at 25°C. The concentration of compound **1c** was maintained at 1 × 10^−5^ M in the cell, while the syringe contained ss-DNA at a concentration of 1 × 10^−4^ M. Both samples were prepared in Tris-HCl buffer at pH 7.4 and degassed for a duration of 10 minutes. Compound **1c** was injected 18 times during the titration schedule. The binding constant (*K*_*b*_), the enthalpy (Δ*H*_*b*_), and the entropy changes (Δ*S*_*b*_) were determined through the nonlinear fitting of normalized titration data using a model of identical and independent binding sites [33].

### 2.8. Molecular Docking

Molecular docking studies between the crystallized structure of the A B-DNA dodecamer (PDB: 1BNA) and diorganotin(IV) derivatives were carried out in the AutoDock 4 program [34]. Water molecules were removed from the crystallized structure. The 3D structures of the compounds were sketched in 2D with ChemSketch and converted into a 3D mol2 format in the Open Babel GUI program [35]. The ligands were optimized with PM6 using the Gaussian 98 software to obtain the lowest energy conformation. All possible rotatable bonds, torsion angles, atomic partial charges, and nonpolar hydrogens were determined for each ligand. The grid dimensions in AutoDockTools were 58 × 58 × 118 Å^3^ with points separated by 0.375 Å and centered at *X* = 14.719, *Y* = 20.979, and *Z* = 8.824. The hybrid Lamarckian genetic algorithm was applied for the minimization utilizing default parameters. A total of one hundred docking runs were conducted, adopting the conformation with the lowest binding energy (kcal/mol) for all further simulations. AutoDockTools was used to prepare the script and files and to visualize the docking results, which were edited in the Discovery 4.0 client.

### 2.9. Procedure for the Synthesis of Ligands (**L1**–**L3**)

The ligands thiosemicarbazone (**L1**), dehydroacetic acid-*N*(4)-methylthiosemicarbazone (**L2**), dehydroacetic acid-*N*(4)-phenylthiosemicarbazone (**L3**) were prepared from dehydroacetic acid and thiosemicarbazide or 4-methyl-3-thiosemicarbazide or 4-phenyl-3-thiosemicarbazide and ethanol as solvent, following the methodology described in the literature by Nechak et al. [36].

### 2.10. General Procedure for the Synthesis of Diorganotin(IV) Derivatives

Thiosemicarbazide, 4-methyl-3-thiosemicarbazide, or 4-phenyl-3-thiosemicarbazide, dehydroacetic acid, and the corresponding diorganotin oxide were added to a solution of 30 ml of toluene : methanol mixture (4 : 1 v/v) in a stoichiometric ratio of 1 : 1 : 1. The reaction mixture was stirred under reflux (8 h for butyl, octyl, and trimethylsilyl methyl; 48 h for cyclohexyl and phenyl derivatives), and the water formed was removed using a Dean-Stark trap. Then, the solvent was removed under reduced pressure to obtain the diorganotin complexes. The amorphous yellow solids were purified by recrystallization in CH_2_Cl_2_/hexane.

#### 2.10.1. 4-Amino-2,2-dibutyl-7,10-dimethylpyrane[3,4-h]-1,3,5,6,2-oxathiadiazastannonin-8-one (**1a**)

Compound **1a** was prepared from 0.0732 g (0.8034 mmol) of thiosemicarbazide, 0.1351 g (0.8034 mmol) of dehydroacetic acid, and 0.2 g (0.8034 mmol) of dibutyltin oxide. A yellow solid was isolated with 77% yield (0.2938 g); melting point (m.p.) 133−135°C (dec); *R*_*f*_ = 0.63 (70% ethyl acetate/hexane); molar conductance, Λ_*M*_ (1 × 10^−3^ M, methanol) = 8.5 ohm^−1^ cm^2^ mol^−1^ (nonelectrolyte); UV-Vis [methanol, *λ*_max_/nm (*ε*/M^−1^ cm^−1^)]: 217 (17648) *π*-*π*^*∗*^ (ligand), 271 (9470) *π*-*π*^*∗*^ (C=N), 363 (6161) n-*π*^*∗*^ (C=N); IR (ATR) cm^−1^: 3382, 3177 *ν*(N-H), 1670 *ν*(OC=O), 1558 *ν*(C=N), 1332 *ν*(C-O_Pyrone_), 1244 *ν*(C-S), 683 *ν*(Sn-O), 520 *ν*(Sn-C), 432 *ν*(Sn-N), 314 *ν*(Sn-S); ^1^H NMR (300.52 MHz, CDCl_3_)*δ*: 0.90 (6H, t, *J* = 7.51 Hz, Sn(CH_2_)_3_CH_3_), 1.27–1.36 (4H, m, Hz, *J* = 7.51 Hz, Sn(CH_2_)_2_CH_2_CH_3_), 1.48–1.54 (4H, m, SnCH_2_(CH_2_)_2_CH_3_), 1.59–1.70 (4H, m, SnCH_2_CH_2_CH_2_CH_3_), 2.19 (3H, d, *J* = 0.90 Hz, H-12), 2.64 (3H, s, H-13), 4.98 (2H, s, NH_2_), 5.74 (1H, d, *J* = 0.90 Hz, H-11); ^13^C NMR (75.57 MHz, CDCl_3_)*δ*: 178.2 (C-8), 168.6 (C-7), 165.1 (C-4), 163.7 (C-11a), 163.1 (C-10), 105.7 (C-11), 99.1 (C-7a), 22.1 (C-13), 19.9 (C-12), 27.4 (Sn(CH_2_)_2_CH_2_CH_3_, ^3^*J*(^13^C-^119^Sn) = 29.47 Hz), 26.5 (SnCH_2_CH_2_CH_2_CH_3_, ^2^*J*(^13^C-^119/117^Sn) = 90.68, 86.91 Hz), 24.2 (SnCH_2_(CH_2_)_2_CH_3_, ^1^*J*(^13^C-^119/117^Sn) = 556.95, 550.90 Hz), 13.6 (Sn(CH_2_)_3_CH_3_); ^119^Sn NMR (112.07 MHz, CDCl_3_)*δ*: −129.2; ^119^Sn NMR (112.07 MHz, DMSO-_d6_)*δ*: −173.7; mass spectrometry (MS): (DART^+^) [*m/z*] (%): [M^+^+1, 474] (100); HR-MS (DART^+^) [*m/z*]: 474.0873 (calc. for C_17_H_27_N_3_O_3_SnS); observed: 474.0884.

#### 2.10.2. 4-Amino-2,2-dioctyl-7,10-dimethylpyrane-[3,4-h]-1,3,5,6,2-oxathiadiazastannonin-8-one (**1b**)

Compound **1b** was obtained from 0.0504 g (0.5537 mmol) of thiosemicarbazide, 0.0931 g (0.5537 mmol) of dehydroacetic acid, and 0.2 g (0.5537 mmol) of dioctyl tin oxide, to produce a yellow oil in a 94% yield (0.3040 g); *R*_*f*_ = 0.61 (70% ethyl acetate/hexane); molar conductance, Λ_M_ (1 × 10^−3^ M, methanol): 21.7 ohm^−1^ cm^2^ mol^−1^ (nonelectrolyte); UV-Vis [methanol, *λ*_max_/nm (*ε*/M^−1^ cm^−1^)]: 217 (20183) *π*-*π*^*∗*^ (aromatic), 305 (10321), *π*-*π*^*∗*^ (C=N), 362 (6777) n-*π*^*∗*^ (C=N); IR (ATR) cm^−1^: 3222, 3179 *ν*(N-H), 1697 *ν*(OC=O), 1544 *ν*(C=N), 1354 *ν*(C-O_Pyrone_), 1234 *ν*(C-S), 695 *ν*(Sn-O), 523 *ν*(Sn-C), 441 *ν*(Sn-N), 325 *ν*(Sn-S); ^1^H NMR (300.52 MHz, CDCl_3_)*δ*: 0.88 (6H, t, *J* = 6.91 Hz, Sn-(CH_2_)_7_-CH_3_), 1.21–1.33 (20H, m_broad_, Sn-CH_2_-CH_2_-CH_2_-(CH_2_)_4_-CH_3_) 1.47–1.54 (4H, m, Sn-CH_2_-(CH_2_)_6_-CH_3_), 1.61–1.68 (4H, m, Sn-CH_2_-CH_2_-CH_2_-(CH_2_)_4_-CH_3_), 2.18 (3H, d, *J* = 0.80 Hz, H-12), 2.64 (3H, s, H-13), 4.97 (2H, s, NH_2_), 5.73 (1H, d, *J* = 0.80 Hz, H-11); ^13^C NMR (75.57 MHz, CDCl_3_)*δ*: 178.2 (C-8), 168.6 (C-7), 165.1 (C-4), 163.7 (C-11a), 163.1 (C-10), 105.7 (C-11), 99.1 (C-7a), 33.5 (Sn-CH_2_-CH_2_-(CH_2_)_5_-CH_3,_^2^*J*(^13^C-^119/117^Sn) = 84.64, 81.62 Hz), 31.8 (Sn-(CH_2_)_6_-CH_2_-CH_3_), 29.2 (Sn-(CH_2_)_3_-CH_2_-(CH_2_)_3_CH_3,_ ^4^*J*(^13^C-^119^Sn) = 15.87 Hz), 29.1 (Sn-(CH_2_)_4_-CH_2_-(CH_2_)_2_CH_3_), 25.3 (Sn-(CH_2_)_2_-CH_2_-(CH_2_)_4_-CH_3_, ^3^*J*(^13^C-^119^Sn) = 29.47 Hz), 24.6 (Sn-CH_2_-(CH_2_)_6_-CH_3_, ^1^*J*(^13^C-^119/117^Sn) = 549.4, 525.2 Hz), 22.7 (Sn-(CH_2_)_5_-CH_2_-CH_2_-CH_3_), 22.1 (C-13), 19.9 (C-12), 14.1 (Sn-(CH_2_)_7_-CH_3_); ^119^Sn NMR (112.07 MHz, CDCl_3_)*δ*: −129.3; ^119^Sn NMR (112.07 MHz, DMSO-_d6_) *δ*: −169.7; MS: (DART^+^) [*m/z*] (%): [M^+^+1, 586] (100); HR-MS (DART^+^) *m/z*: 586.2125 (calc. for C_25_H_44_N_3_O_3_SnS); observed: 586.2139.

#### 2.10.3. 4-Amino-2,2-diphenyl-7,10-dimethylpyrane[3,4-h]-1,3,5,6,2-oxathiadiazastannonin-8-one (**1c**)

Compound **1c** was prepared from 0.1164 g (0.6922 mmol) of thiosemicarbazide, 0.0631 g (0.6922 mmol) of dehydroacetic acid, and 0.2 g (0.6922 mmol) of diphenyl tin oxide. A yellow solid was isolated with 77% yield (0.2723 g); m.p. 86–88°C (dec); *R*_*f*_ = 0.63 (70% ethyl acetate/hexane); molar conductance, Λ_M_ (1 × 10^−3^ M, methanol): 23.0 ohm^−1^ cm^2^ mol^−1^ (nonelectrolyte); UV-Vis [methanol, *λ*_max_/nm (*ε*/M^−1^ cm^−1^)]: UV-Vis [methanol, *λ*max/nm (*ε*/M^−1^ cm^−1^)]: 215 (72627) *π*-*π*^*∗*^ (aromatic), 262 (24119) *π*-*π*^*∗*^ (C=N), 367 (18410) n-*π*^*∗*^ (C=N); IR (ATR) cm^−1^: 3312, 3177 *ν*(N-H), 1694 *ν*(OC=O), 1595 *ν*(C=N), 1354 *ν*(C-O_Pyrone_), 1230 *ν*(C-S), 693 *ν*(Sn-O), 527 *ν*(Sn-C), 439 *ν*(Sn-N), 326 *ν*(Sn-S); ^1^H NMR (300.52 MHz, CDCl_3_)*δ*: 2.17 (3H, d *J* = 0.8 Hz, H-12), 2.68 (3H, s, H-13), 5.07 (2H, s, NH_2_), 5.94 (1H, d *J* = 0.8 Hz H-11), 7.83–7.85 (4H, m, H-*meta*) 7.40–7.42 (6H, m, H-*ortho*, H-*para*); ^13^C NMR (75.57 MHz, CDCl_3_)*δ*: 178.4 (C-8), 169.6 (C-7), 163.53 (C-10), 163.47 (C-11a), 163.3 (C-4), 139.9 (C-*ipso*, ^1^*J*(^13^C-^119/117^Sn) = 902.31, 870.56 Hz), 135.9 (C-*meta*, ^3^*J*(^13^C-^119^Sn) = 58.19 Hz), 130.5 (C-*para*, ^4^*J*(^13^C-^119^Sn) = 16.63 Hz), 128.9 (C-*ortho*, ^2^*J*(^13^C-^119/117^Sn) = 84.64, 81.62 Hz), 105.9 (C-11), 99.5 (C-7a), 22.6 (C-13), 19.9 (C-12); ^119^Sn NMR (112.07 MHz, CDCl_3_)*δ*: −239.9; ^119^Sn NMR (112.07 MHz, DMSO-_d6_)*δ*: −324.2; MS: (DART^+^) [*m/z*] (%): [M^+^+1, 514] (100); HR-MS (DART^+^) *m/z*: 514.0247 (calc. for C_21_H_19_N_3_O_3_SnS); observed: 514.0246.

#### 2.10.4. 4-Amino-2,2-dicyclohexyl-7,10-dimethylpyrane[3,4-h]-1,3,5,6,2-oxathiadiazastannonin-8-one (**1d**)

Compound **1d** was obtained from 0.0605 g (0.6644 mmol) of thiosemicarbazide, 0.1117 g (0.6644 mmol) of dehydroacetic acid, and 0.2 g (0.6644 mmol) of dicyclohexyl tin oxide. A yellow solid was isolated with 79% yield (0.2755 g); m.p. 140−142°C (dec); *R*_*f*_ = 0.82 (70% ethyl acetate/hexane); molar conductance, Λ_M_ (1 × 10^−3^ M, methanol): 6.3 ohm^−1^ cm^2^ mol^−1^ (nonelectrolyte); UV-Vis [methanol, *λ*_max_/nm (*ε*/M^−1^ cm^−1^)]: 212 (22955) *π*-*π*^*∗*^ (ligand), 272 (9324) *π*-*π*^*∗*^ (C=N), 369 (6234) n-*π*^*∗*^ (C=N); IR (ATR) cm^−1^: 3384 *ν*(N-H), 1707 *ν*(OC=O), 1580 *ν*(C=N), 1350 *ν*(C-O_Pyrone_), 1229 *ν*(C-S), 696 *ν*(Sn-O), 527 *ν*(Sn-C), 439 *ν*(Sn-N), 323 *ν*(Sn-S); ^1^H NMR (300.52 MHz, CDCl_3_)*δ*: 1.44–2.00 (20H, m, Sn-CH-(CH_2_)_5_-), 2.18 (2H, m, Sn-CH-(CH_2_)_5_-), 2.27 (3H, d, *J* = 0.60 Hz, H-12), 2.70 (3H, s, H-13), 5.04 (2H, s, NH_2_), 5.85 (1H, d, *J* = 0.6 Hz, H-11); ^13^C NMR (75.57 MHz, CDCl_3_)*δ*: 178.4 (C-8), 168.4 (C-7), 163.8 (C-11a), 163.0 (C-10), 165.1 (C-4), 105.8 (C-11), 99.2 (C-7a), 42.0 (Sn-CH, ^1^*J*(^13^C-^119/117^Sn) = 532.0, 519.9 Hz), 30.44, 30.24 (Sn-CH-CH_2_-CH_2_-(CH_2_)_3_-, ^3^*J*(^13^C-^119^Sn) = 16.63 Hz), 28.67, 28.60 (Sn-CH-CH_2_-(CH_2_)_4_-, ^2^*J*(^13^C-^119/117^Sn) = 92.2, 89.2 Hz), 26.0 (Sn-CH-(CH_2_)_2_-CH_2_-(CH_2_)_2_-, ^4^*J*(^13^C-^119^Sn) = 10.58 Hz); 22.1 (C-13), 19.9 (C-12); ^119^Sn NMR (112.07 MHz, CDCl_3_)*δ*: −172.8; ^119^Sn NMR (112.07 MHz, DMSO-_d6_)*δ*: −184.6; MS: (DART^+^) [*m/z*] (%): [M^+^+1, 526] (100); HR-MS (DART^+^) *m/z*: 526.1183 (calc. for C_21_H_32_N_3_O_3_SnS); observed: 526.1192.

#### 2.10.5. 4-Amino-2,2-bis(trimethylsilyl)methyl-7,10-dimethylpyrane[3,4-h]-1,3,5,6,2-oxathiadiazastannonin-8-one (**1e**)

Compound **1e** was prepared from 0.0589 g (0.6470 mmol) of thiosemicarbazide, 0.1487 g (0.6470 mmol) of dehydroacetic acid, and 0.2 g (0.6470 mmol) of bis[(trimethylsilyl)methyl tin] oxide. A yellow solid was isolated with a 69% yield (0.2386 g); m.p. 40−42°C (dec); *R*_*f*_ = 0.74 (70% ethyl acetate/hexane); molar conductance, Λ_M_ (1 × 10^−3^ M, methanol): 11.6 ohm^−1^ cm^2^ mol^−1^ (nonelectrolyte); UV-Vis [methanol, *λ*_max_/nm (*ε*/M^−1^ cm^−1^)]: 210 (52560) *π*-*π*^*∗*^ (ligand), 265 (24808) *π*-*π*^*∗*^ (C=N), 369 (20794) n-*π*^*∗*^ (C=N); IR (ATR) cm^−1^: 3317, 3180 *ν*(N-H), 1696 *ν*(OC=O), 1595 *ν*(C=N), 1320 *ν*(C-O_Pyrone_), 1246 *ν*(C-S), 827 *ν*(Si-CH_3_), 689 *ν*(Sn-O), 523 *ν*(Sn-C), 437 *ν*(Sn-N), 324 *ν*(Sn-S); ^1^H NMR (300.52 MHz, CDCl_3_)*δ*: 0.00 (18H, s, Si-(CH_3_)_3_), 0.30, 0.53 (4H, AB, *J* = 12.62 Hz, Sn-CH_2_-Si), 2.12 (3H, d, *J* = 0.60 Hz, H-12), 2.59 (3H, s, H-13), 4.91 (2H, s, NH_2_), 5.64 (1H, d, *J* = 0.60 Hz, H-11); ^13^C NMR (75.57 MHz, CDCl_3_)*δ*: 176.7 (C-8), 167.9 (C-7), 163.6 (C-10), 162.5 (C-11a), 162.0 (C-4), 104.6 (C-11), 97.7 (C-7a), 21.3 (C-13), 18.7 (C-12), 9.9 (Sn-CH_2_-Si, ^1^*J*(^13^C-^119/117^Sn) = 448.13, 428.48 Hz), 0.00 (Si-(CH_3_)_3_^1^*J*(^13^C-^29^Si) = 26.9 Hz); ^119^Sn NMR (112.07 MHz, CDCl_3_)*δ*: −118.0; ^119^Sn NMR (112.07 MHz, DMSO-_d6_)*δ*: −129.0; MS: (DART^+^) [*m/z*] (%): [M^+^+1, 534] (63); HR-MS (DART^+^) *m/z*: 534.0725 (calc. for C_17_H_32_N_3_O_3_SnSi_2_S); observed: 534.0723.

#### 2.10.6. 2,2-Dibutyl-7,10-dimethyl-4-(amino methyl) Pyrane[3,4-h]-1,3,5,6,2-oxathiadiazastannonin-8-one (**2a**)

Compound **2a** was prepared from 0.0845 g of 4-methyl-3-thiosemicarbazide (0.8034 mmol), 0.1351 g of dehydroacetic acid (0.8034 mmol), and 0.2 g of dibutyltin oxide (0.8034 mmol). A yellow solid was isolated with a 62% yield (0.2406 g); m.p. 88–90°C (dec); *R*_*f*_ = 0.35 (70% ethyl acetate/hexane); molar conductance, Λ_M_ (1 × 10^−3^ M, methanol): 6.5 ohm^–1^ cm^2^·mol^−1^ (nonelectrolyte); UV-Vis [methanol, *λ*_max_/nm (*ε*/M^−1^ cm^−1^)]: 211 (39515) *π*-*π*^*∗*^ (ligand), 267 (22554) *π*-*π*^*∗*^ (C=N), 367 (16340) n-*π*^*∗*^ (C=N); IR (ATR) cm^−1^: 3305 *ν*(N-H), 1684 *ν*(OC=O), 1552 *ν*(C=N), 1355 *ν*(C-O_Pyrone_), 1237 *ν*(C-S), 582 *ν*(Sn-O), 523 *ν*(Sn-C), 446 *ν*(Sn-N), 352 *ν*(Sn-S); ^1^H NMR (400.13 MHz, CDCl_3_)*δ*: 0.89 (6H, t, *J* = 7.20 Hz, Sn-(CH_2_)_3_-CH_3_), 1.28–1.36 (4H, m, Hz, Sn-(CH_2_)_2_-CH_2_-CH_3_), 1.47–1.52 (4H, m, Sn-CH_2_-(CH_2_)_2_-CH_3_), 1.59–1.67 (4H, m, Sn-CH_2_-CH_2_-CH_2_-CH_3_), 2.18 (3H, d, *J* = 0.40 Hz, H-12), 2.67 (3H, s, H-13), 2.96 (3H, d, *J* = 4.80 Hz, NH-CH_3_), 4.89 (1H, q, *J* = 4.80 Hz, NH-CH_3_), 5.73 (1H, d, *J* = 0.40 Hz, H-11); ^13^C NMR (100.62 MHz, CDCl_3_)*δ*: 178.0 (C-8), 167.7 (C-7), 164.4 (C-4), 163.8 (C-11a), 162.8 (C-10), 105.8 (C-11), 99.1 (C-7a), 30.3 (NH-CH_3_), 27.5 (Sn-(CH_2_)_2_-CH_2_-CH_3_, ^3^*J*(^13^C-^119^Sn) = 29.17 Hz), 26.5 (Sn-CH_2_-CH_2_-CH_2_-CH_3_, ^2^*J*(^13^C-^119^Sn) = 87.54 Hz), 24.1 (Sn-CH_2_-(CH_2_)_2_-CH_3_, ^1^*J*(^13^C-^119/117^Sn) = 564.47, 540.33 Hz), 22.1 (C-13), 19.8 (C-12), 13.6 (Sn-(CH_2_)_3_-CH_3_); ^119^Sn NMR (112.07 MHz, CDCl_3_)*δ*: −135.7; ^119^Sn NMR (112.07 MHz, DMSO-_d6_)*δ*: −177.1; MS: (DART^+^) [*m/z*] (%): [M^+^+1, 488] (100); HR-MS (DART^+^) [*m/z*]: 488.1030 (calc. for C_22_H_21_N_3_O_3_SnS); observed: 488.1034.

#### 2.10.7. 2,2-Dioctyl-7,10-dimethyl-4-(amino methyl) Pyrane[3,4-h]-1,3,5,6,2-oxathiadiazastannonin-8-one (**2b**)

Compound **2b** was obtained from 0.0582 g of 4-methyl-3-thiosemicarbazide (0.5537 mmol), 0.0931 g of dehydroacetic acid (0.5537 mmol), and 0.2 g of dioctyl tin oxide (0.5537 mmol). A yellow solid oil was isolated with a 91% yield (0.3025 g); *R*_*f*_ = 0.58 (70% ethyl acetate/hexane); molar conductance, Λ_M_ (1 × 10^−3^ M, methanol): 15.8 ohm^–1^·cm^2^·mol^−1^ (nonelectrolyte); UV-Vis [methanol, *λ*_max/nm_(*ε*/M^−1^ cm^−1^)]: 217 (18153) *π*-*π*^*∗*^ (ligand), 269 (10879) *π*-*π*^*∗*^ (C=N), 370 (9588) n-*π*^*∗*^ (C=N); IR (ATR) cm^−1^: 3345 *ν*(N-H), 1693 *ν*(OC=O), 1547 *ν*(C=N), 1353 *ν*(C-O_Pyrone_), 1232 *ν*(C-S), 582 *ν*(Sn-O), 522 *ν*(Sn-C), 441 *ν*(Sn-N), 355 *ν*(Sn-S); ^1^H NMR (400.13 MHz, CDCl_3_)*δ*: 0.88 (6H, t, *J* = 7.20 Hz, Sn-(CH_2_)_7_-CH_3_), 1.26–1.29 (20H, m, Sn-CH_2_-CH_2_-CH_2_-(CH_2_)_4_-CH_3_), 1.47–1.53 (4H, m, Sn-CH_2_-(CH_2_)_6_-CH_3_), 1.61–1.67 (4H, m, Sn-(CH_2_)_2_-CH_2_-(CH_2_)_4_CH_3_), 2.17 (3H, s, H-12), 2.71 (3H, s, H-13), 2.95 (3H, d, *J* = 4.80 Hz, NH-CH_3_), 4.87 (1H, q, *J* = 4.80 Hz, NH-CH_3_), 5.73 (1H, s, H-11); ^13^C NMR (100.62 MHz, CDCl_3_)*δ*: 178.2 (C-8), 168.7 (C-7), 164.4 (C-4), 163.8 (C-11a), 162.8 (C-10), 106.0 (C-11), 99.5 (C-7a), 33.5 (Sn-(CH_2_)_2_-CH_2_-(CH_2_)_4_CH_3_, ^2^*J*(^13^C-^119^Sn) = 82.51 Hz), 31.8(Sn-(CH_2_)_6_-CH_2_-CH_3_), 30.2 (NH-CH_3_), 29.2 (Sn-(CH_2_)_3_-CH_2_-(CH_2_)_3_CH_3_, ^4^*J*(^13^C-^119^Sn) = 27.16 Hz), 25.3 (Sn-(CH_2_)_2_-CH_2_-(CH_2_)_4_CH_3_, ^3^*J*(^13^C-^119^Sn) = 28.17 Hz), 24.5 (Sn-CH_2_-(CH_2_)_6_-CH_3_, ^1^*J*(^13^C-^119/117^Sn) = 550.4, 527.3 Hz), 22.7 (Sn-(CH_2_)_5_-CH_2_-CH_2_-CH_3_), 22.1 (C-13), 19.8 (C-12), 14.1 (Sn-(CH_2_)_7_-CH_3_); ^119^Sn NMR (149.21 MHz, CDCl_3_)*δ*: −135.8; ^119^Sn NMR (149.21 MHz, DMSO-_d6_)*δ*: −173.2; MS: (DART^+^) [*m/z*] (%): [M^+^+1, 600] (100); HR-MS: (DART^+^) [*m/z*]: 600.2282 (calc. for C_26_H_46_N_3_O_3_SnS); observed: 600.2254.

#### 2.10.8. 2,2-Diphenyl-7,10-dimethyl-4-(amino methyl) Pyrane[3,4-h]-1,3,5,6,2-oxathiadiazastannonin-8-one (**2c**)

Compound **2c** was prepared from 0.0728 g of 4-methyl-3-thiosemicarbazide (0.6922 mmol), 0.1164 g of dehydroacetic acid (0.6922 mmol), and 0.2 g of diphenyl tin oxide (0.6922 mmol). A yellow solid was isolated with 81% yield (0.2946 g); m.p. 180–182°C (dec); *R*_*f*_ = 0.33 (70% ethyl acetate/hexane); molar conductance, Λ_M_ (1 × 10^−3^ M, methanol): 20.1 ohm^–1^ cm^2^·mol^−1^ (nonelectrolyte); UV-Vis [methanol, *λ*_max/nm_(*ε*/M^−1^ cm^−1^)]: 212 (41955) *π*-*π*^*∗*^ (aromatic), 264 (13759) *π*-*π*^*∗*^ (aromatic, C=N), 370 (9920) n-*π*^*∗*^ (C=N); IR (ATR) cm^−1^: 3308 *ν*(N-H), 1681 *ν*(OC=O), 1547 *ν*(C=N), 1352 *ν*(C-O_Pyrone_), 1228 *ν*(C-S), 581 *ν*(Sn-O), 528 *ν*(Sn-C), 448 *ν*(Sn-N), 364 *ν*(Sn-S); ^1^H NMR (400.13 MHz, CDCl_3_)*δ*: 2.17 (3H, d, *J* = 0.80 Hz, H-12), 2.71 (3H, s, H-13), 2.96 (3H, d, *J* = 4.80 Hz, NH-CH_3_), 5.00 (1H, q, *J* = 4.80 Hz, NH-CH_3_), 5.94 (1H, d, *J* = 0.80 Hz, H-11), 7.84–7.86 (4H, m, H-*meta*) 7.39–7.42 (6H, m, H-*ortho*, H-*para*); ^13^C NMR (100.62 MHz, CDCl_3_)*δ*: 178.2 (C-8), 168.7 (C-7), 163.6 (C-4), 163.2 (C-11a), 162.7 (C-10), 140.2 (C-*ipso*, ^1^*J*(^13^C-^119/117^Sn) = 1088.71, 849.23 Hz), 135.9 (C-*meta*, ^3^*J*(^13^C-^119^Sn) = 57.35 Hz), 130.4 (C-*para*, ^4^*J*(^13^C-^119^Sn) = 17.10 Hz), 128.9 (C-*ortho*, ^2^*J*(^13^C-^119^Sn) = 83.51 Hz), 106.0 (C-11), 99.5 (C-7a), 30.1 (NH-CH_3_), 22.6 (C-13), 19.9 (C-12); ^119^Sn NMR (149.21 MHz, CDCl_3_)*δ*: −246.0; ^119^Sn NMR (149.21 MHz, DMSO-_d6_)*δ*: −326.8; MS: (DART^+^) [*m/z*] (%): [M^+^+1, 528] (100); HR-MS: (DART^+^) [*m/z*]: 528.0404 (calc. for C_22_H_22_N_3_O_3_SnS); observed: 528.0386.

#### 2.10.9. 2,2-Dicyclohexyl-7,10-dimethyl-4-(amino methyl) Pyrane[3,4-h]-1,3,5,6,2-oxathiadiazastannonin-8-one (**2d**)

Compound **2d** was obtained from 0.0699 g of 4-methyl-3-thiosemicarbazide (0.6644 mmol), 0.1117 g of dehydroacetic acid (0.6644 mmol), and 0.2 g of dicyclohexyl tin oxide (0.6644 mmol), affording 0.2479 g (71%) of a yellow solid; m.p. 144–146°C (dec); *R*_*f*_ = 0.34 (70% ethyl acetate/hexane); molar conductance, Λ_M_(1 × 10^−3^ M, methanol): 18.7 ohm^–1^ cm^2^ mol^−1^ (nonelectrolyte); UV-Vis [methanol, *λ*_max/nm_(*ε*/M^−1^ cm^−1^)]: 212 (25395) *π*-*π*^*∗*^ (ligand), 272 (12311) *π*-*π*^*∗*^ (C=N), 368 (6596) n-*π*^*∗*^ (C=N); IR (ATR) cm^−1^: 3344 *ν*(N-H), 1701 *ν*(OC=O), 1545 *ν*(C=N), 1353 *ν*(C-O_pyrone_), 1233 *ν*(C-S), 583 *ν*(Sn-O), 523 *ν*(Sn-C), 437 *ν*(Sn-N), 356 *ν*(Sn-S); ^1^H NMR (400.13 MHz, CDCl_3_)*δ*: 1.35–1.93 (20H, m, Sn-CH-(CH_2_)_5_-) 2.03–2.13 (2H, m, Hz, Sn-CH-(CH_2_)_5_-), 2.19 (3H, d, *J* = 0.6 Hz, H-12), 2.65 (3H, s, H-13), 2.95 (3H, d, *J* = 4.80 Hz, NH-CH_3_), 4.88 (3H, q, *J* = 4.80 Hz, NH-CH_3_), 5.77 (1H, d, *J* = 0.60 Hz, H-11); ^13^C NMR (100.62 MHz, CDCl_3_)*δ*: 178.2 (C-8), 167.5 (C-7), 164.4 (C-4), 163.9 (C-11a), 162.7 (C-10), 105.9 (C-11), 99.2 (C-7a), 41.7 (Sn-CH-(CH_2_)_5_-, ^1^*J*(^13^C-^119/117^Sn) = 535.79, 523.70 Hz), 30.4 (NH-CH_3_), 30.28, 30.22 (Sn-CH-CH_2_-CH_2_-(CH_2_)_3_-, ^3^*J*(^13^C-^119^Sn) = 24.18 Hz), 28.67, 28.61 (Sn-CH-CH_2_-(CH_2_)_4_- ^2^*J*(^13^C-^119/117^Sn) = 83.88, 80.86 Hz), 26.6 (Sn-CH-(CH_2_)_2_-CH_2_-(CH_2_)_2_-, ^4^*J*(^13^C-^119/^Sn) = 9.82 Hz), 22.1 (C-13), 19.9 (C-12); ^119^Sn NMR (149.21 MHz, CDCl_3_)*δ*: −178.6; ^119^Sn NMR (149.21 MHz, DMSO-_d6_)*δ*: −189.4; MS: (DART^+^) [*m/z*] (%): [M^+^+1, 540] (100); HR-MS: (DART^+^) [*m/z*]: 540.1343 (calc. for C_22_H_33_N_3_O_3_SnS); observed: 540.1353.

#### 2.10.10. 2,2-Bis(trimethylsilyl)methyl-7,10-dimethyl-4-(aminomethyl) Pyrane[3,4-h]-1,3,5,6,2-oxathiadiazastannonin-8-one (**2e**)

Compound **2e** was synthesized from 0.0680 g of 4-methyl-3-thiosemicarbazide (0.6470 mmol), 0.1087 g of dehydroacetic acid (0.6470 mmol), and 0.2 g of bis[(trimethylsilyl)methyl tin] oxide (0.6470 mmol). A yellow solid was isolated with a 60% yield (0.2107 g); m.p. 133–135°C (dec); *R*_*f*_ = 0.34 (70% ethyl acetate/hexane); molar conductance, Λ_M_ (1 × 10^−3^ M, methanol): 0.0 ohm^–1^ cm^2^ mol^−1^ (nonelectrolyte); UV-Vis [methanol, *λ*_max_/nm (*ε*/M^−1^ cm^−1^)]: 210 (41725) *π*-*π*^*∗*^ (ligand), 266 (22178) *π*-*π*^*∗*^ (C=N), 379 (15391) n-*π*^*∗*^ (C=N).; IR (ATR) cm^−1^: 3381 *ν*(N-H), 1692 *ν*(OC=O), 1556 *ν*(C=N), 1355 *ν*(C-O_Pyrone_), 1250 *ν*(C-S), 829 *ν*(Si-CH_3_), 587 *ν*(Sn-O), 519 *ν*(Sn-C), 437 *ν*(Sn-N), 360 *ν*(Sn-S); ^1^H NMR (400.13 MHz, CDCl_3_)*δ*: 0.00 (18H, s, Si-(CH_3_)_3_), 0.30, 0.52 (4H, AB, *J* = 12.40 Hz, Sn-CH_2_-Si), 2.12 (3H, s, H-12), 2.63 (3H, s, H13), 2.89 (3H, d, *J* = 4.80 Hz, NH-CH_3_), 4.82 (3H, q, *J* = 4.80 Hz, NH-CH_3_), 5.65 (1H, s, H-11); ^13^C NMR (100.62 MHz, CDCl_3_)*δ*: 176.5 (C-8), 166.9 (C-7), 163.0 (C-4), 162.7 (C-11a), 161.7 (C-10), 104.7 (C-11), 97.8 (C-7a), 28.9 (NH-CH_3_), 21.4 (C-13), 18.7 (C-12), 9.92 (Sn-CH_2_-Si, ^1^*J*(^13^C-^119/117^Sn) = 450.78, 430.65 Hz), 0.00 (Si-(CH_3_)_3_, ^1^*J*(^13^C-^29^Si) = 26.16 Hz); ^119^Sn NMR (149.21 MHz, CDCl_3_)*δ*: −125.4; ^119^Sn NMR (149.21 MHz, DMSO-_d6_)*δ*: −133.1; MS: (DART^+^) [*m/z*] (%): [M^+^+1, 548] (100); HR-MS: (DART^+^) [*m/z*]: 505.0881 (calc. for C_18_H_33_N_3_O_3_SnSi_2_S); observed: 548.0895.

#### 2.10.11. 2,2-Dibutyl-7,10-dimethyl-4-(aminophenyl) Pyrane[3,4-h]-1,3,5,6,2-oxathiadiazastannonin-8-one (**3a**)

Compound **3a** was prepared from 0.1343 g of 4-phenyl-3-thiosemicarbazide (0.8034 mmol), 0.1351 g of dehydroacetic acid (0.8034 mmol), and 0.2 g of dibutyltin oxide (0.8034 mmol). A yellow solid was isolated with 80% yield (0.3504 g); m.p. 118–120°C (dec); *R*_*f*_ = 0.34 (70% ethyl acetate/hexane); molar conductance, Λ_M_ (1 × 10^−3^ M, methanol): 13.0 ohm^–1^ cm^2^ mol^−1^ (nonelectrolyte); UV-Vis [methanol, *λ*_max_/nm (*ε*/M^−1^ cm^−1^)]: 209 (48272) *π*-*π*^*∗*^ (aromatic), 256 (29619) *π*-*π*^*∗*^ (aromatic), 274 (26818) *π*-*π*^*∗*^ (C=N), 376 (23380) n-*π*^*∗*^ (C=N); IR (ATR) cm^−1^: 3384, 3290 *ν*(N-H), 1683 *ν*(OC=O), 1553 *ν*(C=N), 1353 *ν*(C-O_Pyrone_), 1238 *ν*(C-S), 690 *ν*(Sn-O), 527 *ν*(Sn-C), 440 *ν*(Sn-N), 322 *ν*(Sn-S); ^1^H NMR (400.13 MHz, CDCl_3_)*δ*: 0.89 (6H, t, *J* = 7.60 Hz, Sn-(CH_2_)_3_-CH_3_), 1.27–1.37 (4H, m, Sn-(CH_2_)_2_-CH_2_-CH_3_), 1.53–1.57 (4H, m, Sn-CH_2_-(CH_2_)_2_-CH_3_), 1.62–1.70 (4H, m, Sn-CH_2_-CH_2_-CH_2_-CH_3_), 2.20 (3H, d, *J* = 0.80 Hz, H-12), 2.73 (3H, s, H-13), 5.76 (1H, d, *J* = 0.80 Hz, H-11), 6.89 (1H, s, NH-Ph), 7.04 (1H, t, *J* = 7.60 Hz, H-*para*), 7.31 (2H, t, *J* = 8.4 Hz, H-*meta*), 7.52 (2H, dd, *J* = 0.8 Hz, *J* = 8.4 Hz, H-*ortho*); ^13^C NMR (100.62 MHz, CDCl_3_)*δ*: 178.4 (C-8), 169.6 (C-7), 163.7 (C-119a), 163.3 (C-10), 160.9 (C-4), 139.8 (C-*ipso*), 128.9 (C-*meta*), 123.0 (C-*para*), 119.8 (C-*ortho*), 105.8 (C-11), 99.2 (C-7a), 22.9 (C-13), 19.9 (C-12), 27.5 (Sn-(CH_2_)_2_-CH_2_-CH_3_, ^3^*J*(^13^C-^119^Sn) = 28.17 Hz), 26.5 (Sn-CH_2_-CH_2_-CH_2_-CH_3_, ^2^*J*(^13^C-^119^Sn) = 87.54 Hz), 24.1 (Sn-CH_2_-(CH_2_)_2_-CH_3_, ^1^*J*(^13^C-^119/117^Sn) = 554.41, 537.31 Hz), 13.6 (Sn-(CH_2_)_3_-CH_3_); ^119^Sn NMR (149.21 MHz, CDCl_3_)*δ*: −141.2; ^119^Sn NMR (149.21 MHz, DMSO-_d6_)*δ*: −197.5; MS: (DART^+^) [*m/z*] (%): [M^+^+1, 550] (100); HR-MS: (DART^+^) [*m/z*]: 550.1186 (calc. for C_23_H_31_N_3_O_3_SnS); observed: 550.1194.

#### 2.10.12. 2,2-Dioctyl-7,10-dimethyl-4-(aminophenyl) Pyrane[3,4-h]-1,3,5,6,2-oxathiadiazastannonin-8-one (**3b**)

Compound **3b** was obtained from 0.0926 g of 4-phenyl-3-thiosemicarbazide (0.5537 mmol), 0.0931 g of dehydroacetic acid (0.5537 mmol), and 0.2 g of dioctyl tin oxide (0.5537 mmol). A yellow solid was isolated with a 74% yield (0.3074 g); m.p. 73–75°C (dec); *R*_*f*_ = 0.58 (70% ethyl acetate/hexane); molar conductance, Λ_M_ (1 × 10^−3^ M, methanol): 5.1 ohm^–1^ cm^2^ mol^−1^ (nonelectrolyte); UV-Vis [methanol, _*λ*max_/nm (*ε*/M^−1^ cm^−1^)]: 217 (33149) *π*-*π*^*∗*^ (aromatic), 250 (24877) *π*-*π*^*∗*^ (aromatic), 283 (18833) *π*-*π*^*∗*^ (C=N), 379 (22784) n-*π*^*∗*^ (C=N); IR (ATR) cm^−1^: 3301 *ν*(N-H), 1687 *ν*(OC=O), 1551 *ν*(C=N), 1353 *ν*(C-O_Pyrone_), 1235 *ν*(C-S), 690 *ν*(Sn-O), 523 *ν*(Sn-C), 442 *ν*(Sn-N), 324 *ν*(Sn-S); ^1^H NMR (400.13 MHz, CDCl_3_)*δ*: 0.87 (6H, t, *J* = 7.20 Hz, Sn-(CH_2_)_7_-CH_3_), 1.24–1.30 (20H, m, Sn-CH_2_-CH_2_-CH_2_-(CH_2_)_4_-CH_3_), 1.52–1.56 (4H, m, Sn-CH_2_-(CH_2_)_6_-CH_3_), 1.64–1.69 (4H, m, Sn-(CH_2_)_2_-CH_2_(CH_2_)_4_-CH_3_), 2.20 (3H, s, H-12), 2.73 (3H, s, H-13), 5.75 (1H, s, H-11), 6.77 (1H, s, NH-Ph), 7.04 (1H, t, *J* = 7.51 Hz, H-*para*), 7.31 (2H, t, *J* = 7.51 Hz, H-*meta*), 7.51 (2H, d, *J* = 7.51 Hz, H-*ortho*); ^13^C NMR (100.62 MHz, CDCl_3_)*δ*: 178.4 (C-8), 169.6 (C-7), 163.7 (C-11a), 163.3 (C-10), 160.9 (C-4), 139.7 (C-*ipso*), 128.9 (C-*meta*), 123.0 (C-*para*), 119.7 (C-*ortho*), 105.8 (C-11), 99.2 (C-7a), 33.5 (Sn-CH_2_-CH_2_-(CH_2_)_5_-CH_3_, ^2^*J*(^13^C-^119^Sn) = 83.51 Hz), 31.8 (Sn-(CH_2_)_6_-CH_2_-CH_3_), 29.2 (Sn-(CH_2_)_3_-CH_2_-(CH_2_)_3_-CH_3_, ^4^*J*(^13^C-^119^Sn) = 24.15 Hz), 29.1 (Sn-(CH_2_)_4_-CH_2_-(CH_2_)_2_-CH_3_), 25.3 (Sn-(CH_2_)_2_-CH_2_-(CH_2_)_4_-CH_3_, ^3^*J*(^13^C-^119^Sn) = 28.17 Hz), 24.6 (Sn-CH_2_-(CH_2_)_6_-CH_3_, ^1^*J*(^13^C-^119/117^Sn) = 548.38, 526.24 Hz), 22.7 (Sn-(CH_2_)_5_-(CH_2_)-CH_2_-CH_3_), 22.9 (C-13), 19.9 (C-12), 14.1 (Sn-(CH_2_)_7_-CH_3_); ^119^Sn NMR (149.21 MHz, CDCl_3_)*δ*: −141.4; ^119^Sn NMR (149.21 MHz, DMSO-_d6_)*δ*: −195.3; MS: (DART^+^) [*m/z*] (%): [M^+^+1, 662] (100); HR-MS: (DART^+^) [*m/z*]: 661.5130 (calc. for C_31_H_48_N_3_O_3_SnS); observed: 590.0559.

#### 2.10.13. 2,2-Diphenyl-7,10-dimethyl-4-(aminophenyl) Pyrane[3,4-h]-1,3,5,6,2-oxathiadiazastannonin-8-one (**3c**)

Compound **3c** was synthesized from 0.1157 g of 4-phenyl-3-thiosemicarbazide (0.6922 mmol), 0.1164 g of dehydroacetic acid (0.6922 mmol), and 0.2 g of diphenyl tin oxide (0.6922 mmol). A yellow solid was isolated with a 53% yield (0.2152 g); m.p. 105–107°C (dec); *R*_*f*_ = 0.37 (70% ethyl acetate/hexane); molar conductance Λ_M_ (1 × 10^−3^ M, methanol): 38.0 ohm^–1^ cm^2^ mol^−1^ (nonelectrolyte); UV-Vis [methanol, *λ*_max_/nm (*ε*/M^−1^ cm^−1^)]: 209 (40624) *π*-*π*^*∗*^ (aromatic), 243 (17797) *π*-*π*^*∗*^ (aromatic), 280 (13617) *π*-*π*^*∗*^ (C=N), 382 (10849) n-*π*^*∗*^ (C=N); IR (ATR) cm^−1^: 3384, 3290 *ν*(N-H), 1683 *ν*(OC=O), 1549 *ν*(C=N), 1352 *ν*(C-P_yrone_), 1227 *ν*(C-S), 691 *ν*(Sn-O), 526 *ν*(Sn-C), 439 *ν*(Sn-N), 330 *ν*(Sn-S); ^1^H NMR (400.13 MHz, CDCl_3_)*δ*: 2.19 (3H, d, *J* = 0.80 Hz, H-12), 2.77 (3H, s, H-13), 5.96 (1H, d, *J* = 0.80, 8.4 Hz, H-11), 6.94 (1H, s, NH-Ph), 7.04 (1H, t, *J* *=* 7.6 Hz, H-*para* NH-Ph), 7.31 (2H, t, *J* = 7.6 Hz, H-*meta* NH-Ph), 7.51 (2H, dd, *J* = 0.8 Hz, *J* = 7.6 Hz, H-*ortho* NH-Ph), 7.85–7.87 (4H, m, H-*meta* (Sn-Ph)), 7.40–7.42 (6H, m, H-*ortho, para*, (Sn-Ph)); ^13^C NMR (100.62 MHz, CDCl_3_)*δ*: 178.6 (C-8), 170.6 (C-7), 163.8 (C-11a), 163.5 (C-10), 159.2 (C-4), 139.52 (C-*ipso*), 139.48 (C-*ipso*, ^1^*J*(^13^C-^119/117^Sn) = 916.64, 873.38 Hz), 135.9 (C-*meta*, ^3^*J*(^13^C-^119^Sn) = 57.35 Hz), 130.6 (C-*para*, ^4^*J*(^13^C-^119^Sn) = 16.09 Hz), 129.0 (C-*ortho*, ^2^*J*(^13^C-^119^Sn) = 83.51 Hz), 128.9 (C-*meta*), 123.3 (C-*para*), 120.0 (C-*ortho*), 106.0 (C-11), 99.6 (C-7a), 23.4 (C-13), 20.0 (C-12); ^119^Sn NMR (149.21 MHz, CDCl_3_)*δ*: −251.0; ^119^Sn NMR (149.21 MHz, DMSO-_d6_)*δ*: −347.6; MS: (DART^+^) [*m/z*] (%): [M^+^+1, 590] (100); HR-MS: (DART^+^) [*m/z*]: 590.0560 (calc. for C_27_H_23_N_3_O_3_SnS); observed: 590.0573.

#### 2.10.14. 2,2-Dicyclohexyl-7,10-dimethyl-4-(aminophenyl) Pyrane[3,4-h]-1,3,5,6,2-oxathiadiazastannonin-8-one (**3d**)

Compound **3d** was prepared from 0.1111 g of 4-phenyl-3-thiosemicarbazide (0.6644 mmol), 0.1117 g of dehydroacetic acid (0.6644 mmol), and 0.2 g of dicyclohexyl tin oxide (0.6644 mmol). A yellow solid was isolated with a 62% yield (0.2477 g); m.p. 88–90°C (dec); *R*_*f*_ = 0.34 (70% ethyl acetate/hexane); molar conductance, Λ_M_(1 × 10^−3^ M, methanol): 8.0 ohm^–1^ cm^2^ mol^−1^ (nonelectrolyte); UV-Vis [methanol, *λ*_max/nm_(*ε*/M^−1^ cm^−1^)]: 208 (41803) *π*-*π*^*∗*^ (aromatic), 261 (22246) *π*-*π*^*∗*^ (aromatic), 274 (20921) *π*-*π*^*∗*^ (C=N), 381 (12140) n-*π*^*∗*^ (C=N); IR (ATR) cm^−1^: 3302 *ν*(N-H), 1684 *ν*(COO), 1549 *ν*(C=N), 1352 *ν*(C-O_Pyrone_), 1232 *ν*(C-S), 634 *ν*(Sn-O), 523 *ν*(Sn-C), 440 *ν*(Sn-N), 325 *ν*(Sn-S); ^1^H NMR (400.13 MHz, CDCl_3_)*δ*: 1.35–1.94 (20H, m, Sn-CH-(CH_2_)_5_-), 2.10–2.17 (2H, m, Sn-CH-(CH_2_)_5_-), 2.20 (3H, d, *J* = 0.80 Hz, H-12), 2.71 (3H, s, H-13), 5.79 (1H, d, *J* = 0.80 Hz, H-11), 6.82 (1H, s, NH-Ph), 7.03 (1H, tt, *J* = 1.20 Hz, *J* = 8.40 Hz, H-*para*), 7.31 (2H, t, *J* = 8.40 Hz, H-*meta*), 7.51 (2H, dd, *J* = 1.20 Hz, *J* = 8.80 Hz, H-*ortho*); ^13^C NMR (100.62 MHz, CDCl_3_)*δ*: 178.6 (C-8), 169.4 (C-7), 163.8 (C-11a), 163.2 (C-10), 160.7 (C-4), 139.9 (C-*ipso*), 128.9 (C-*meta*), 122.8 (C-*ortho*), 119.7 (C-*para*), 105.8 (C-11), 99.3 (C-7a), 40.0 (C-*α*, ^1^*J*(^13^C-^119/117^Sn) = 531.27, 507.12 Hz), 30.51, 30.22 (Sn-CH-CH_2_-CH_2_-(CH_2_)_3_-, ^3^*J*(^13^C-^119^Sn) = 23.14 Hz), 28.69, 28.63 (Sn-CH-CH_2_-(CH_2_)_4_-, ^2^*J*(^13^C-^119^Sn) = 82.50 Hz), 26.5 (Sn-CH-(CH_2_)_2_-CH_2_-(CH_2_)_2-_), 23.0 (C-13), 19.9 (C-12); ^119^Sn NMR (149.21 MHz, CDCl_3_)*δ*: −184.7; ^119^Sn NMR (149.21 MHz, DMSO-_d6_)*δ*: −203.0; MS: (DART^+^) [*m/z*] (%): [M^+^+1, 602] (100); HR-MS: (DART^+^) [*m/z*]: 602.1499 (calc. for C_27_H_35_N_3_O_3_SnS); observed: 602.1510.

#### 2.10.15. 2,2-Bis(trimethylsilyl)methyl-7,10-dimethyl-4-(aminophenyl) Pyrane[3,4-h]-1,3,5,6,2-oxathiadiazastannonin-8-one (**3e**)

Compound **3e** was prepared from 0.1082 g of 4-phenyl-3-thiosemicarbazide (0.6470 mmol), 0.1087 g of dehydroacetic acid (0.6470 mmol), and 0.2 g (0.6470 mmol) of bis[(trimethylsilyl)methyl tin] oxide. A yellow solid was isolated with 64% yield (0.2514 g); m.p. 58–60°C (dec); *R*_*f*_ = 0.34 (70% ethyl acetate/hexane); molar conductance, Λ_M_ (1 × 10^−3^ M, methanol): 43.0 ohm^−1^ cm^2^ mol^−1^ (nonelectrolyte); UV-Vis [methanol, *λ*_max/nm_(*ε*/M^−1^ cm^−1^)]: 208 (29306) *π*-*π*^*∗*^ (aromatic), 260 (16746) *π*-*π*^*∗*^ (aromatic), 279 (14863) *π*-*π*^*∗*^ (C=N), 382 (11798) n-*π*^*∗*^ (C=N); IR (ATR) cm^−1^: 3316 *ν*(N-H), 1684 *ν*(OC=O), 1557 *ν*(C=N), 1352 *ν*(C-O_pyrone_), 1246 *ν*(C-S), 830 *ν*(Si-CH_3_), 693 *ν*(Sn-O), 526 *ν*(Sn-C), 447 *ν*(Sn-N), 342 *ν*(Sn-S); ^1^H NMR (400.13 MHz, CDCl_3_) *δ*: 0.00 (18H, s, Si-(CH_3_)_3_), 0.34, 0.55 (4H, AB, *J* = 12.8 Hz, H-Sn-CH_2_-Si), 2.13 (3H, d, *J* = 0.40 Hz, H-12), 2.68 (3H, s, H-13), 5.65 (1H, d, *J* = 0.4 Hz, H-11), 6.65 (1H, s, NH-Ph), 6.97 (1H, tt, *J* = 1.20 Hz, *J* = 8.40 Hz, H-*para*), 7.24 (2H, t, *J* = 8.40 Hz, H-*meta*), 7.43 (2H, dd, *J* = 1.20 Hz, *J* = 8.80 Hz, H-*ortho*); ^13^C NMR (100.62 MHz, CDCl_3_)*δ*: 176.8 (C-8), 168.8 (C-7), 162.4 (C-11a), 162.2 (C-10), 159.4 (C-4), 138.5 (C-*ipso*), 127.7 (C-*meta*), 121.8 (C-*para*), 118.6 (C-*ortho*), 104.6 (C-11), 97.8 (C-7a), 22.1 (C-13), 18.7 (C-12), 9.90 (Sn-CH_2_-Si, ^1^*J*(^13^C-^119/117^Sn) = 447.76, 428.64 Hz), 0.00 (Si-(CH_3_)_3_, ^1^*J*(^13^C-^29^Si) = 26.16 Hz); ^119^Sn NMR (149.21 MHz, CDCl_3_)*δ*: −129.5; ^119^Sn NMR (149.21 MHz, DMSO-*d*_6_)*δ*: −142.0; MS: (DART^+^) [*m/z*] (%): [M^+^+1, 610] (100); HR-MS: (DART^+^) [*m/z*]: 610.1038 (calc. for C_23_H_36_N_3_O_3_SnSi_2_S); observed: 610.1024.

## 3. Results and Discussion

**Scheme 1:**
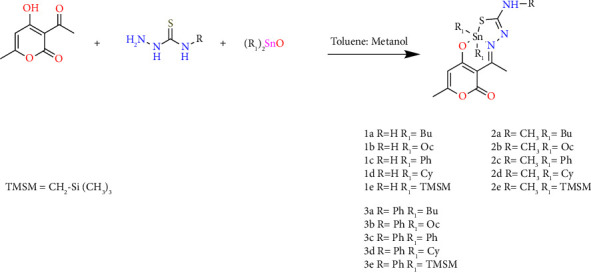
Synthesis of diorganotin(IV) complexes **1a–1e**, **2a–2e**, and **3a–3e** through a multicomponent reaction.

Organotin(IV) complexes were synthesized in a one-pot reaction as shown in Scheme 1. In a three-component reaction, dehydroacetic acid was combined with thiosemicarbazide, 4-methyl-3-thiosemicarbazide or 4-phenyl-3-thiosemicarbazide, and the corresponding diorganotin(IV) oxide in a stoichiometric ratio (1 : 1 : 1). The reaction proceeded to provide the complexes in moderate to good yields of 53 to 94% as shown in Scheme 1. The organotin complexes(IV) were soluble in organic solvents (chloroform, dichloromethane, methanol, ethanol, and DMSO). The molar conductivity at room temperature using methanol as solvent showed values ranging from 5.1 to 43.0 Ω^−1^ cm^2^·mol^−1^, indicating the absence of counter ions, where the donor atoms of the ligand were covalently bonded [37]. Considering that the biological response of organotin compounds can be modified by the ligand, the nature of the donor atoms, and the substituents coordinated to the tin atom. The molecular structures of the ligand were varied on the nitrogen atom from the thiosemicarbazone moiety. The latter was substituted by methyl and phenyl, and the substituents on the tin atom were from the acyclic alkyls (Bu and Oc) to cyclic (Cy) and bulky (Ph and trimethylsilyl methyl (TMSM)) groups. All these compounds were purified by crystallization in the dichloromethane-hexane mixture.

### 3.1. Electronic Absorption

**Figure 1:**
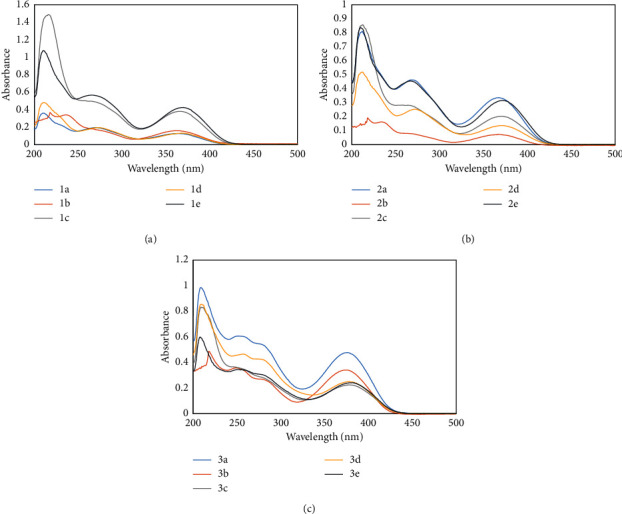
Ultraviolet-visible (UV-Vis) spectra in methanol of diorganotin (IV) complexes.

The electronic spectra of the organotin(IV) complexes (Figure 1) were measured in a solution of methanol (2.04510 × 10^−5^ M); the series of complexes showed similar absorption bands ranging from 210 to 217 nm and associated with the *π*-*π*^*∗*^ of the aromatic systems transition, and the **3a**–**3e** complexes revealed two bands at 215 and 284 nm. The bands at 265 to 280 nm and 362 to 382 nm were assigned to the n-*π*^*∗*^ electronic transitions of the azomethine (C=N) group and *π*-*π*^*∗*^ metal-ligand transfer charge (MLTC) and were in the range reported for organotin(IV) complexes [26, 38]. Upon the utilization of dichloromethane as the solvent (Figure S1), two absorption bands for **1a**–**2e** were detected and **3a**–**3e** displayed three, and the increase in the polarity produced a slight blue shift (∼10 nm) associated with the HOMO stabilization in the polar solvent [39].

### 3.2. Fourier Transform (FT)-IR

An absence of the characteristic bands corresponding to OH at 3257 and C=S at 1150 cm^−1^ of the ligand [36] was observed in the IR spectra of all complexes (Figures S2–S16), indicative of the formation of organotin complexes. The complexation process occurred through the tautomerization of the ligand towards the thiol form, followed by its deprotonation. The presence of only one vibration band for the carbonyl group *υ*(C=O) over the 1670–1707 cm^−1^ range for the pyrone corroborates the condensation of the thiosemicarbazide with the carbonyl group of DHA and the formation of azomethine occurred. The stretching vibrational band of the latter was observed around *υ*(C=N) 1544–1595 cm^−1^. A comparative analysis of these values with the free ligand *υ*(C=N) 1628 cm^−1^ stretch described in the literature revealed a shift towards lower energies as a consequence of the coordination of the nitrogen atom via the azomethine towards the metallic center [40].

Additional evidence for the successful formation of organotin(IV) complexes was provided by the identification of the characteristic vibrational frequencies corresponding to *υ*(Sn-C), *υ*(Sn-O), *υ*(Sn-C), and (Sn-S), as shown in Table 1; for complexes **1d**, **2d**, and **3d**, stretching vibrations *υ*(Si-C) were identified from 827 to 829 cm^−1^.

### 3.3. Mass Spectrometry

**Figure 2:**
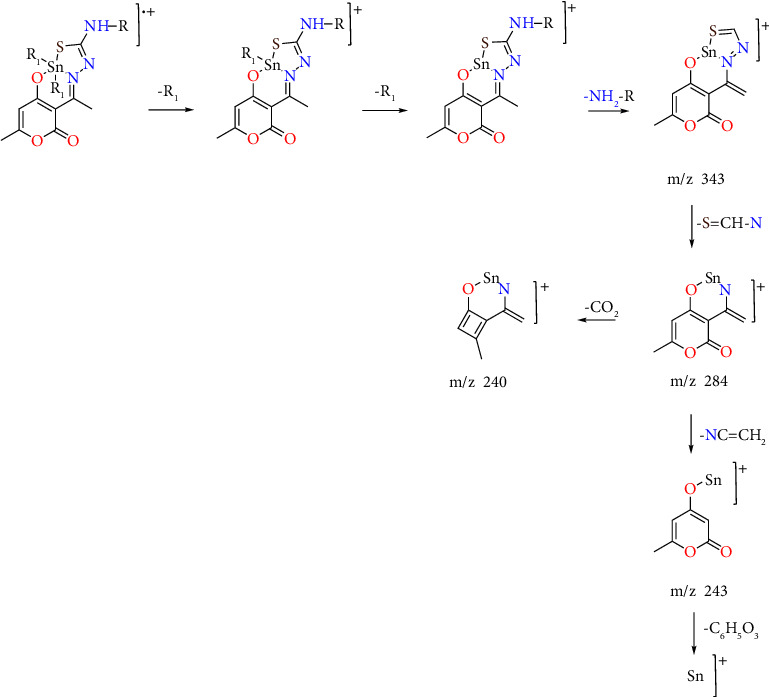
Proposed mass fragmentation pattern of organotin (IV) complexes.

The samples of all complexes were analyzed using direct analysis in real time (DART-MS). The spectra showed a high relative abundance of [M + H]^+^ molecular ions and were consistent with the expected structure and isotopic patterns, supporting the formation of discrete monomeric compounds. Compounds **1a–1e**, **2a**, **2d**, **3a**, and **3d** were also analyzed by electron impact; fragment ions [M − *R*_1_]^+^ and [M − 2*R*_1_]^+^ were detected, corresponding to the subsequent loss of the organic groups attached to the tin atom. The peaks at m/z 343, 284, 243, and 240 were observed, indicating the loss of different organic groups. This allowed us to propose the fragmentation pattern as shown in Figure 2. The isotopic pattern of the molecular ion peaks observed for all complexes was expected for a single tin atom (Figures S17–S35).

### 3.4. Nuclear Magnetic Resonance (NMR) Spectroscopy

The ^1^H NMR spectra of the complexes (Figures S36–S50) showed the expected signals, chemical shifts, and integrals, which were consistent with the structure of the complexes and are listed in the experimental section. The disappearance of aldehyde and thiosemicarbazide signals indicates that a condensation reaction occurred. The absence of OH and SH signals corroborates the double deprotonation due to the coordination of tin. Signals related to the organic groups (Bu, Oc, Ph, Cy, and CH_2_-SiMe_3_) were detected in the aliphatic or aromatic region. In addition, two single signals in the aliphatic due to the methyl groups of the DHA moiety were observed in the regions of 2.17–2.20 and 2.64–2.71 ppm for H-12 and H-13, respectively.

The ^13^C NMR spectra revealed four signals in the aliphatic region for the organic butyl groups **1a**, **2a**, and **3a** and eight for the octyl groups **1b**, **2b**, and **3b** attached to the metallic center. Signals of the *ipso*, *ortho, meta*, and *para* carbons for phenyl derivatives **1c**, **2c**, and **3c** were identified. However, in the case of the **1d**, **2d**, and **3d** complexes, the cyclohexyl moieties gave rise to eight signals, indicating the inequivalence of substituents due to the high steric demand and conformation of the cyclohexyl rings. The **1e**, **2e**, and **3e** complexes also exhibited nonequivalent protons for the methylene group of the bis(trimethylsilyl)methyl bonded to the tin atom. An AB system was observed alongside a single signal for the methyl groups (the ^13^C NMR spectra of all complexes are shown in Figures S51–S65). The 2D experiments corroborated the assignment of the individual signals. Figures S66–S73 show the HSQC and HMBC experiments involving the **1a**–**1c** and **1e** complexes.

Additionally, satellite signals were observed for all complexes, and coupling constants ^*1*^*J* (^13^C-^119^Sn), ^*2*^*J* (^13^C-^119^Sn), ^*3*^*J* (^13^C-^119^Sn), and ^*4*^*J* (^13^C-^119^Sn) were measured. The Lockhart–Manders and Holeček equations [41, 42] and the values of the ^*1*^*J* (^13^C-^119^Sn) were used to calculate the C-Sn-C bond angles in solution (Table 2), which ranged from 119 to 144° [43].

The ^119^Sn NMR spectra were acquired in noncoordinated and coordinated solvents (Figures S74–S103); single signals were observed in both cases (Table 2). The coordination numbers in the solution could be associated with the ^119^Sn chemical shifts; the values depended on the electronic environment around the tin, whereby the concentrations of dialkyl tin complexes ranged from −90 to −190 ppm for the five-coordinated compounds and from −210 to −400 ppm for the six-coordinated compounds [42]. The chemical shifts of the aryl groups occurred at higher fields than those of the aliphatic ones. The chemical shifts for all complexes corresponded to a pentacoordinated environment in both noncoordinated (CDCl_3_) and coordinated solvents (DMSO) and agreed with the ^119^Sn NMR spectra of the pentacoordinated organotins(IV) derived from the Schiff base ligands [26, 44].

### 3.5. X-Ray Crystal Structure Analysis

**Figure 3:**
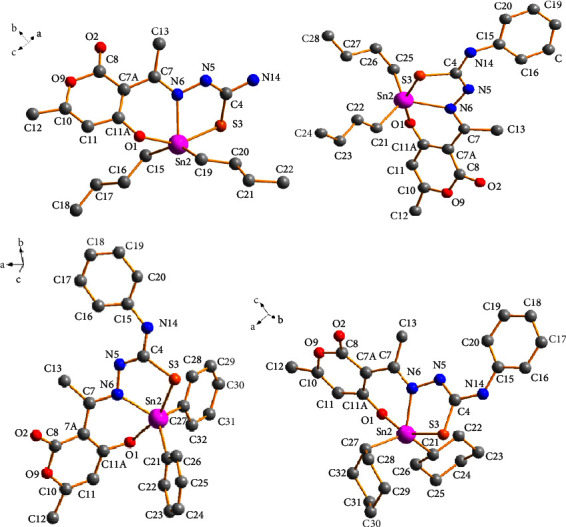
Molecular structure of compounds **1a**, **3a**, **3c**, and **3d**.

Suitable crystals for single-crystal X-ray diffraction (SCXRD) analyses of compounds **1a**, **3a**, **3c**, and **3d** were obtained from the concentrate solvent mixtures: heptane : dichloromethane; methanol : chloroform; hexane : dichloromethane; and methanol, respectively. The four compounds possess similar structural features, and their molecular structures are illustrated in Figure 3, while the crystal data are listed in Table S1. Selected bond lengths and angles are listed in Table 3. Compounds **3a** and **3c** crystallized in the monoclinic space group P2_1_/c, while compounds **2a** and **3d** were formed in the orthorhombic system space groups Pbcn and P2_1_2_1_2_1_, respectively.

All compounds were monomeric molecular structures in which the tin center was coordinated by two carbon atoms from the alkyl or aryl groups and a nitrogen, oxygen, and sulfur atom from the tridentate Schiff base ligand. The resulting pentacoordinated geometry defined by the C_2_NOS donor set was distorted from an ideal trigonal bipyramidal geometry; the indices of trigonality of the plane were calculated using the equation *τ* = (*β*-*α*)/60 [45, 46], where the most considerable donor-metal-donor angles in the five-coordinated environment defined *α* and *β*. A value of *τ* = 1 corresponded to a perfect trigonal-bipyramidal geometry, whereas *τ* = 0 corresponded to a perfect square pyramidal structure.

For compounds **1a**, **3a**, **3c**, and **3d,** the *τ* values calculated were 0.10, 0.59, 0.39, and 0.40, respectively, indicating that compound **3a** possessed a distorted trigonal bipyramidal geometry in which the two carbon atoms of *n*-butyl groups (C21 and C25) and the imine nitrogen (N6) held an equatorial position. At the same time, the oxygen (O1) and sulfur (S3) atoms occupy an apical position. The *τ* values for compounds **1a**, **3c**, and **3d** indicated a distorted square-pyramidal geometry, with oxygen-(O1), thiolate-sulfur (S3) atoms, and two carbon atoms (C15 and C19 or C21 and C27) occurring at the basal positions and the azomethine nitrogen (N6) at the apical position. The preference for a square pyramidal over the trigonal bipyramidal geometry was likely the result of the packing effects and intermolecular contacts.

According to the angle values (Table 3), the distortions of the trigonal bipyramidal or square pyramidal geometries of the complexes were also evidenced by the O(1)-Sn-(2)-S (3) angles which were smaller than 180° (151.8(3)–158.0(1)°). The primary distortion in the coordination geometry around the tin center in compounds **1a**, **3a**, **3c**, and **3d** may have been attributed to the formation of five- (Sn2, S3, C4, N5, and N6) and six-membered (Sn2, O1, N6, C7, C7A, and C11A) chelated rings from the tridentate ligand, resulting in tight N(6)-Sn(2)-O(1) [78.1(1)°–80.3(1)°] and N6-Sn2-S3 [75.52(4)°–77.8(1)°] chelate angles which were lower than 90°. The dihedral angles involving these chelate rings ranged from 0.42 (5)° to 45.1(3)°, indicating the nonplanarity of the ligand plane around the tin atom (see Table 3).

The distance Sn(2)-S(3) of the five-membered ring ranged from 2.563(1) to 2.507(2) Å for compounds **1a 3a**, **3c**, and **3d**, while for Sn(2)-O(1) at the six-membered ring, the distances ranged from 2.140(4) to 2.276(12) Å; both distances were close to the sum of the covalent radii of the Sn-S (2.44 Å) and Sn-O (2.10 Å) atoms, respectively, but smaller than those of the van der Waals radii between the Sn and S (4.0 Å) and Sn and O (3.75 Å) atoms. These values indicate that the complexes formed strong Sn-S and Sn-O bonds. The Sn (2)-N (6) bond distances ranged from 2.218(2) to 2.193(2), which were slightly larger than the sum of the covalent radii for Sn and N (2.10 Å), suggesting a strong tin-nitrogen interaction comparable to the analogous diorganotin(IV) complexes containing both five- and six-membered chelate rings including a set of ONS donor atoms. [47, 48].

The Sn-C bond lengths ranged from 2.176 to 2.117 Å and were quite similar to one another. However, the C-Sn-C angles were different; for compounds **1a**, **3a**, and **3d,** these angles were 145.7(1), 131.2(2), and 129.0(2)°, respectively, i.e., significantly wider than the equivalent angle of, 122.2 (3)° for **3c**. The bond angles for complexes **3a**, **3c**, and **3d** were similar to those calculated for the compounds hosted in the solution.

The bond lengths C(4)-N(5) and C(4)-(S3) ranged from 1.313(2) Å to 1.288(6) Å and 1.795(5) to 1.750(5) Å, respectively, indicative of a C(4)-N(5) double bond. On the other hand, C(4)-(S3) represented a single bond as a result of the tautomerism and electron delocalization of the C=S double bound on the SCN moiety during the complexation process, which was consistent with the results of the IR and NMR analysis. These values agreed with those described for similar complexes [29, 49, 50].

**Figure 4:**
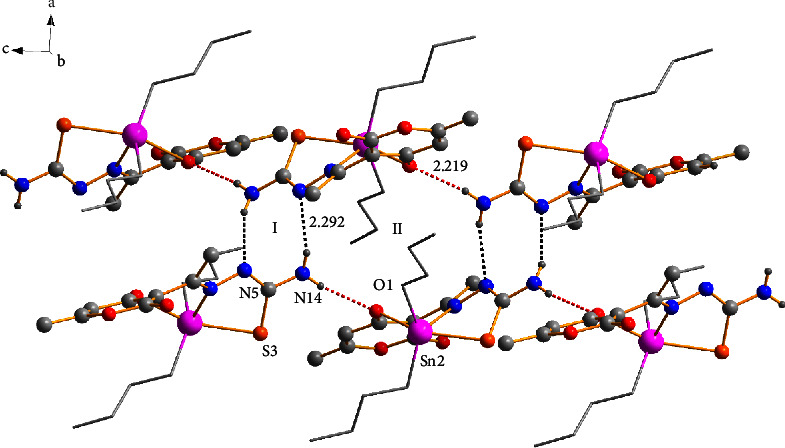
The formation of a 1D polymeric chain through hydrogen bonds -N-H···N (black dots) and -N-H···O (red dots).

The crystalline lattice of compound **1a** showed an interaction between the hydrogen atom (H14B) of the donor amine with one neighboring nitrogen atom (N5) of the thiosemicarbazide group with a bond distance of (N5) ⋯H(14B) (2.292 Å) giving rise to the formation of homosynthon **I** (eight-membered ring). Besides, the hydrogen bond —N–H···O of the amine group (H14A) with the oxygen atom (O1) of the six-membered ring participated in the formation of synthon **II {**(macrocycle [*R*$\left(\begin{array}{c} 2\\ 2 \end{array}\right)$ (14)]} [51] with a bond distance of (O1)⋯H(14A) (2.219 Å) which helped to assemble the 1D polymeric chain (Figure 4).

**Figure 5:**
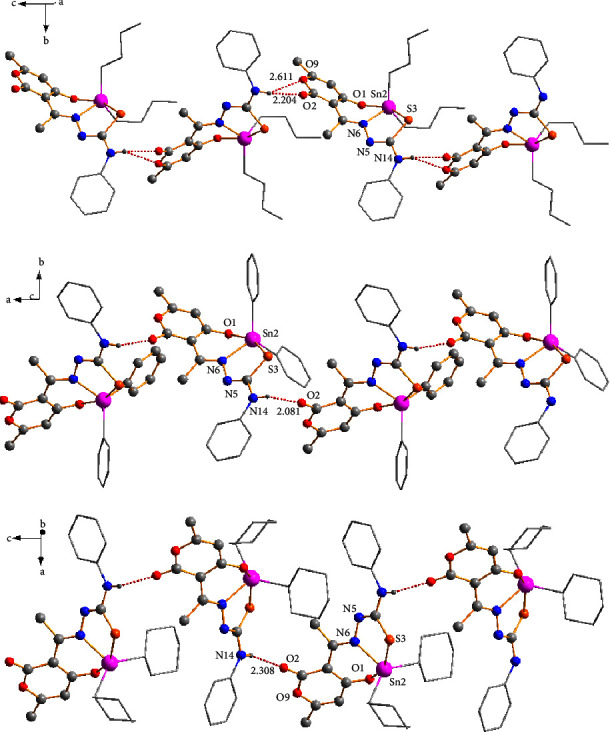
The formation of 1D polymeric chains was helped by N-H⋯O hydrogen bonds (red dots) in compounds **3a** (up), **3c** (middle), and **3d** (down).

Compounds **3a**, **3c**, and **3d** formed 1D polymeric chains in the crystalline lattice (Figure 5) supported by amine -N-H···O hydrogen bonds. Compound **3a** had a bifurcated interaction between the hydrogen atom (H14) of the donor amine with two neighboring oxygen atoms (O2 and O9) of the dehydroacetic acid ring and C=O group with bond distances of (O2)⋯H(14) (2.204 Å) and (O9)⋯H(14) (2.611 Å), respectively. On the other hand, only the first interaction with the oxygen atom (O2) of the C=O group was observed with bond distances of 2.081 Å for **3c** and 2.308 Å for **3d** for compounds **3c** and **3d** (the selected intermolecular interactions are provided in Table S2).

**Figure 6:**
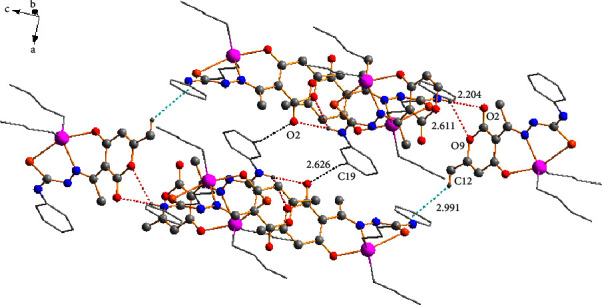
Two-dimensional (2D) polymeric structure for **3a** supported by C-H⋯O (black dots) and C-H⋯*π* (turquoise dots) intermolecular interactions.

**Figure 7:**
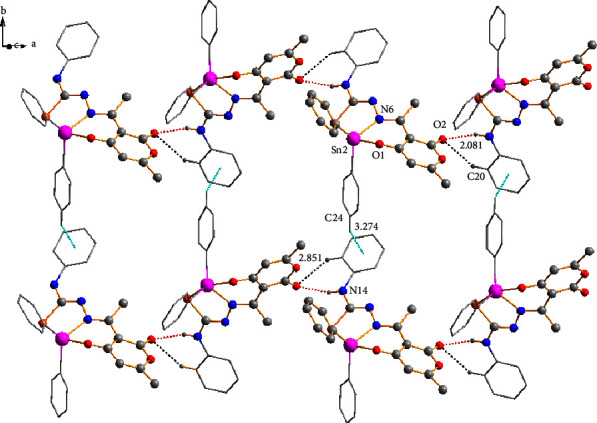
One-dimensional (ID) polymeric chains of compound **3c** connected by C-H⋯O (black dots) and C-H⋯*π* (turquoise dots) intermolecular interactions.

**Figure 8:**
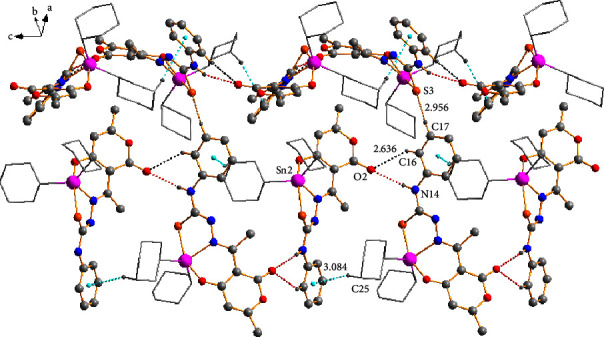
Two-dimensional (2D) polymeric structure of compound **3d** supported by C-H⋯O (black dots), C-H⋯S (orange dots), and C-H⋯*π* (turquoise dots) intermolecular interactions.

At the supramolecular level, secondary interactions were also crucial in the crystalline lattices of the four compounds; the structural analysis of compounds **3a**, **3c**, and **3d** also revealed the presence of C-H⋯O and C-H···*π* interactions 2.629 Å and 2.991 Å for **3a**, 2.851 Å, and 3.271 Å for **3c**, and 2.636 Å and 3.084 Å for **3d** which involves the dehydroacetic acid and phenyl aromatic rings giving rise to the formation of 2D polymeric structures (Figure 6). In compound **3a**, the C-H⋯O interaction participated in the construction of a centrosymmetric macrocycle R $\left(\begin{array}{c} 2\\ 2 \end{array}\right)$ (16) [51] together with a hydrogen bond of the amine group (N14-H14⋯O2). Moreover, compounds **3c** and **3d**, in conjunction with the hydrogen bond of the amine group, formed a six-membered {···HNC⋯OHC} heterosynthon which helped assemble small cages into one-dimensional (ID) polymeric chains in compound **3c** (Figure 7). In addition, for compound **3d**, the C-H···S contacts (2.956 Å) between the hydrogen atom H-17 of the phenyl ring with the sulfur atom (S3) of another molecule helped to form a 2D polymeric chain (Figure 8).

**Figure 9:**
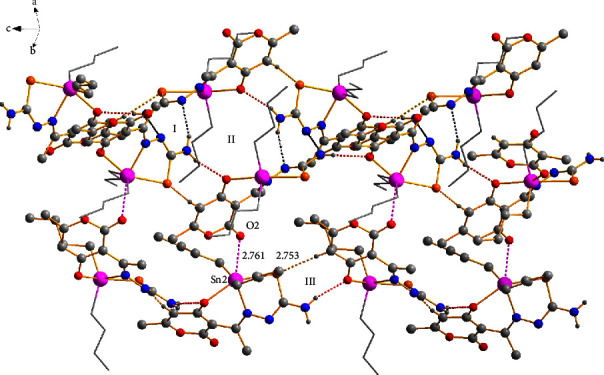
Three-dimensional (3D) polymeric structure of compound **1a** supported by hydrogen bonds N-H⋯N (black dots), N-H⋯O (red dots), and intermolecular interactions C-H⋯S (orange dots) and C-O⋯Sn (pink dots).

In compound **1a**, the C-H⋯S (2.761 Å) interaction participated in the formation of a heterosynthon **III** (eight-membered ring) together with a hydrogen bond of the amine group (N14-H14B O1) and joined by the interaction (O2)⋯Sn (2) with a bond distance of (2.761 Å) forming a 3D polymeric structure (Figure 9).

### 3.6. Stability

The stability of complexes **1a–3e** was examined using UV-Vis spectroscopy prior to the cytotoxic evaluation (Fig. S104). The complexes were solubilized in a DMSO-Tris-HCl buffer (3 : 7 v/v). After 72 h, the absorption pattern bands of the complexes remained unchanged and neither a significant red nor blue shift was observed; additionally, no new bands appeared, indicating that all compounds were stable in the solvent systems used. As representative examples, the spectra of complexes **1a**, **1d, 2a**, **2d**, and **3a**, in the DMSO-d6 solution, were acquired from the time of preparation until 72 h later. These complexes displayed good stability, and there were no observed changes in chemical shifts, signal intensity or multiplicity, or the presence of any additional signals (Figures S105–S110).

### 3.7. Antiproliferative Activity Structure-Activity Relationship

**Figure 10:**
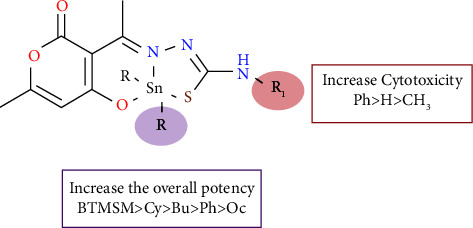
Structure-activity relationship (SAR) of organotin (IV) complexes.

All synthesized complexes were screened for their cytotoxic activity using the sulforhodamine B proliferation assay against two human cancer cell lines MCF-7 and MDA-MB-231 (human mammary adenocarcinoma) and a nonhuman cell line COS-7 (African green monkey kidney fibroblast). Following an incubation period of 72 h, the IC_50_ (*µ*M) values (summarized in Table 4) were measured and Cisplatin was used as a positive control. In general, the IC_50_ values suggest that the compounds were more cytotoxic on MDA-MB-231 than on MCF-7 cells and their cytotoxic effect was significantly more potent than that of Cisplatin. The ligands L1–L3 were synthesized to test their cytotoxicity in comparison to the organotin(IV) complexes, on the MDA-MB231 and the normal cell lines, at a concentration of 20 *µ*M, L1–L2 were found noncytotoxic, whereas L-3 exhibited an antiproliferative effect on both normal and MCF-7 cell lines. However, the organotin complexes were remarkably more cytotoxic. The **1b**, **2b**, and **3b** complexes (Octyl substituents attached to the tin atom) exhibited a less cytotoxic effect against both cancer cell lines. Regarding the N-substitution of the thiosemicarbazone moiety, the N-phenyl derivatives (**3a–3e**) were found to be more potent than thiosemicarbazone (**1a–1e**), followed by the N-methyl derivatives (**2a–2e**), against both MDA-MB-231 and MCF-7 (Figure 10). The analysis of the cytotoxicity based on the substitution of the organic substituents on the metallic center and the IC_50_ values evidenced that compounds **1b**, **2b**, and **3b,** whose substituents bonded to the tin atom were octyl, were the least cytotoxic, while the cyclohexyl and bis(trimethylsilyl)methyl (BTMSM) substituents were the most potent. The steric hindrance provided by the BTMSM substituents increased the cell growth inhibition against the two cancer cell lines. The structure-activity relationship analysis suggests that the order cytotoxicity followed the overall trend BTMSM > Cy > Bu > Ph > Oc (Figure 10). The CLogP (octanol/water partition coefficient) values are provided in Table 4, which were calculated using Molinspiration cheminformatics software. The order of lipophilicity was Oc > BTMSM > Ph ≈ Cy > Bu. Based on the cytotoxic trend, it is possible that the lipophilic-hydrophilic balance might aid in the diffusion and absorption across the cell membrane. The complexes were also evaluated towards a normal cell line (COS-7), showing a selectivity towards cancer cell lines; nondetectable cell proliferation was observed at concentrations from 0.01 to 0.2 *µ*M (Table S3). The selectivity index (SI) values indicated that compounds **2b** and **3e** emerged as the most selective for the MCF-7 cell line and **1a** and **1c** for the MDA-MB-231 one, respectively (Table 4). Additionally, the organotin(IV) complexes exhibited a higher selectivity than Cisplatin on both cancer cell lines compared to the normal reference one. Therefore, the organotin(IV) complex may have had a less toxic effect on normal cells than Cisplatin.

The comparison of IC_50_ values obtained for **1a–3e** with analogous complexes, prepared from 2-hydroxy-5-methoxybenzaldehyde-N(4)-methylthiosemicarbazone and tested on human colorectal cancer cells (HCT 116) with IC_50_ values ranged from 3.5 to 7.58 *µ*M [52], reveals that replacing the aromatic ring on the thiosemicarbazone with a DHA fragment results in a substantial increase in cytotoxicity on breast cancer cell lines (MCF7 and MDA-MB-231).

### 3.8. Morphological Changes on Breast Cancer Cell Lines

**Figure 11:**
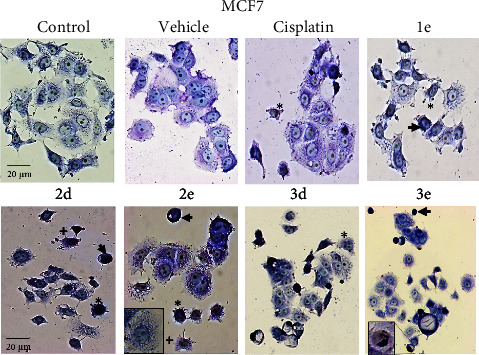
Diorganotin complexes induced morphological changes in the MCF7 breast cancer cell line. Giemsa stain of cells incubated with different organotin (IV) complexes concentrations for 48 h. Cell size reduction and rounding (black arrows), cytoplasm vacuolization (**2e**, box), chromatin condensation and pyknotic nuclei (**3e**, box), apoptotic bodies (star), cell lysis and necrosis (+). Bar: 20 *µ*m (20x).

**Figure 12:**
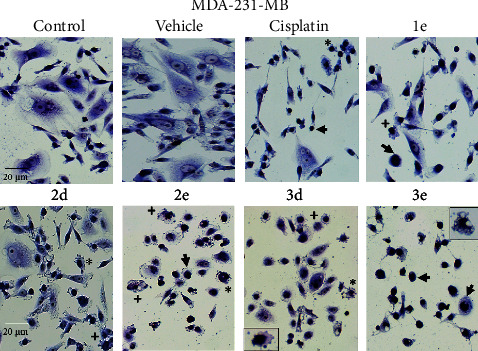
Diorganotin complexes induced morphological changes in the MDA-MB-231 breast cancer cell line. Giemsa stain of cells incubated with different organotin (IV) complexes concentrations for 48 h. Cell size reduction, rounding and pyknotic nuclei (black arrows), cytoplasm vacuolization (**3e**, box), chromatin condensation (**3d**, box), apoptotic bodies (star), cell lysis and necrosis (+). Bar: 20 *µ*m (20x).

Morphological alterations on MCF-7 and MDA-MB-231 were analyzed by Giemsa stain and phase contrast microscopy. After 48 h treatment, both control and vehicle cells showed normal size and morphology. In contrast, cells treated with different organotin(IV) derivates, showed cell size reduction, significant membrane damage and swelling (blebbing), nuclear condensation (dark, condensed, and rounded nuclei), degradation of nuclear chromatin, cytoplasmic vacuolization and the formation of apoptotic bodies (as depicted in Figures 11 and 12). Cell density decreased for **2b** and **3e** on MCF-7 cells, and **1a** and **1c** for MDA-MB-231. The compound **3e** produced the presence of nonadherent cells. These morphological alterations suggested different stages of apoptosis easily distinguishable [53, 54]. As reported previously, organotin(IV) compounds have been suggested as potential for developing anticancer agents, due to their ability to bind to DNA and interact through intercalating interaction and induce apoptosis [55].

### 3.9. Interaction of Organotin (IV) Complexes with DNA, Fluorescence Study

**Figure 13:**
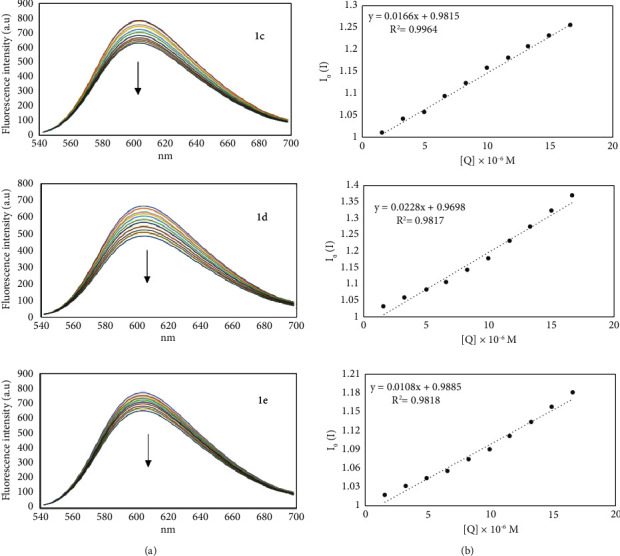
Fluorescence emission spectra (a). The arrow showing the increase in the concentration of complexes **1c**, **1d**, and **1e** represents the change in intensity. The Stern–Volmer plots are displayed on the (b).

The ability of the complexes **1c**, **1d,** and **1e** to interact with DNA, was assessed through competitive displacement assays; these complexes were selected as representative examples of their balance between lipophilicity and potency.  Figure 13 shows the emission spectra of the ss-DNA-EB in the presence of increasing concentrations of organotin complexes from 1.66 *µ*M to 16.6 *µ*M. The results show that, as the amount of the organotin(IV) increases, the fluorescence intensity of the ss-DNA-EB solution decreases, indicating that the organotin(IV) complexes have the ability to release EB from the DNA, which is indicative of an intercalative binding mode for the interaction of these compounds with DNA [56]. The Stern- Volmer plots allowed us to calculate the quenching constant K_sv_ [57], 1.66 × 10^4^, 1.08 × 10^4^, and 2.28 × 10^4^ mol/L for **1c**, **1d,** and **1e,** respectively, the data evidence a strong interaction of the organotin(IV) complexes with the DNA following the order **1e** > **1c** > **1d**.

### 3.10. DNA Interaction of Organotin Complex **1a** by Isothermal Titration Calorimetry (ICT)

**Figure 14:**
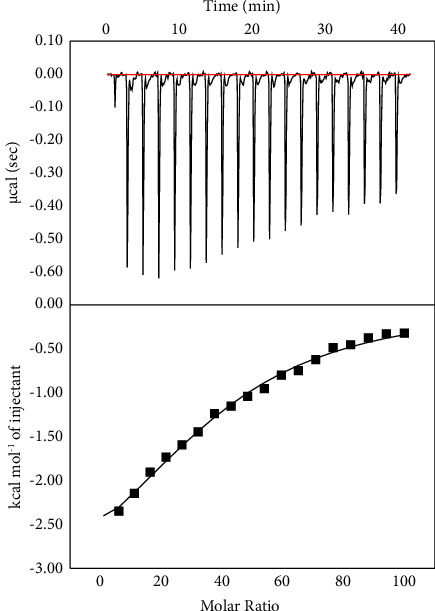
Interaction of complex **1c** with ss-DNA in Tris-HCl 10 mM. The upper panel displays the raw ITC data for the binding, while the lower panel shows the integrated normalized data fitted into an independent binding model.

Isothermal Titration Calorimetry was used to examine the interaction between ss-DNA and the organotin(IV) complex **1c** as a representative example of the series. The ITC curve shows the outcomes of injecting **1c** into an ss-DNA solution at 25°C (Figure 14). The plot exhibits the heat evolved per mole of the organotin(IV) complex added, and against the molar ratio of the complex **1c** to ss DNA, the thermodynamic parameters for the binding interaction of **1c** with ss-DNA were determined using a single set of identical site models and are listed in Table 5. The titration curve reveals an exothermic reaction between the complex **1c** and ss-DNA, resulting in negative peaks on time plot vs *µ*cal/sec. The binding Gibbs free energy change (ΔGb) was obtained from the equation ΔG = −RT ln K, where *R* = 1.987 cal·mol^−1^·K^−1^ and *T* = 298.15 K, the calculated value for the binding reaction of −6.3474 kcal·mol^−1^ of the complex to ss-DNA indicates that the process was driven by both enthalpy and entropy.

## 4. Molecular Docking Studies

As previously described, a molecular docking study between the diorganotin(IV) derivatives and A B-DNA dodecamer was conducted for other organotin complexes [58–60]. The results in Table 6 show that such derivatives recognized the DNA fragments, which suggests a probable binding mechanism for such a molecule known to be one of the main therapeutic targets of antitumor drugs [61]. It should be noted that the binding energy values of all derivatives were lower than the Cisplatin reference compound, which is commonly used as a control in antitumor therapy (−7.18 kcal/mol), namely compounds **3c** (−9.92), **3d** (−10.12) and **3e** (−9.29 Kcal/mol)], which have been shown to have better binding energy. These observations suggest that the synthesized diorganotin(IV) derivatives, when intercalated with DNA fragments, could be used in anticancer therapy, as reported in other studies for other organotins [55, 59, 62, 63].

In addition, it is important to note that substituting the N4 of thiosemicarbazone of the pyrane[3,4-*h*]-1,3,5,6,2-oxathiadiazastannonin-8-one system with a radical increases the affinity of the complexes towards the DNA. In this study was found that better binding energies were observed as a result of the presence of the phenyl group, due to the increase in the electronic density on the aromatic ring through a positive mesomeric effect with the amine. This effect increases the electronic density of the aromatic, allowing it to interact favorably with the pyrimidine-type *π*-electron deficient heterocyclic ring of the DC3 DNA fragment through *π*-*π*T-shaped interaction, as seen with **3e**. The same effect was observed in interaction **3a** with the pyrimidine and imidazole heterocyclic rings of the guanine system in DG4. On the other hand, the substituent on the tin atom affects the affinity; longer alkyl chains result in a weaker interaction due to the different conformations that the alkyl chain can adopt, limiting its ability to intercalate with the DNA in comparison with other radicals, this effect explains the lower affinity of complexes **1b**, **2b** and **3b** with DNA, these findings correlated with biological activity data showing higher IC_50_ values. However, the presence of the cyclohexyl substituent enhances the interaction with the DNA, by exhibiting additional *π*-alkyl interactions with the guanine heterocyclic system of DG4 with **1d** and DG22 with **3d**.

**Figure 15:**
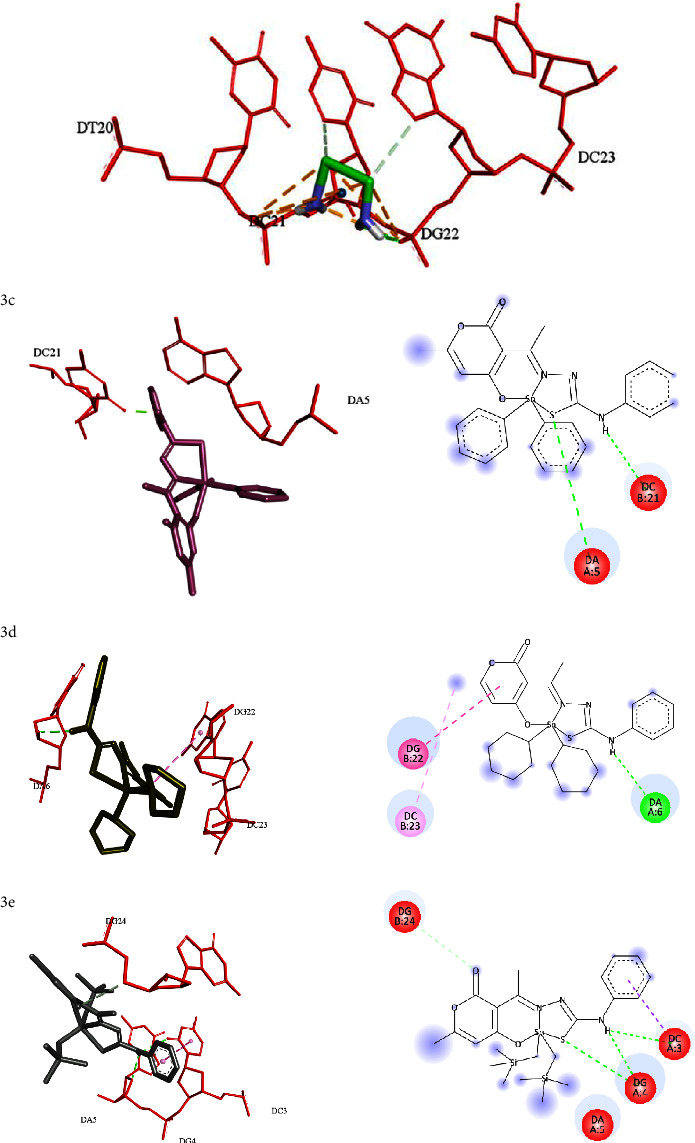
Representation of the interactions between the A B-DNA dodecamer and diorganotin (IV) derivatives **3c-e** and Cisplatin. The interactions and DNA residues are shown (red) in the 3D and 2D models. Conventional hydrogen bonds (dark green dotted lines), carbon-hydrogen bonds (light green), *π*-sigma (purple), *π*-*π* T-shaped (Fuchsia), *π*-alkyl (pink), and *π*-anions (orange) are also shown in 3D and 2D.

Of note, some of the DNA fragments were conserved in most of the derivatives (Figure 15), such as DG4, DA6, DC21, DG22, and DC23, among others, forming hydrophilic and hydrophobic interactions, with the hydrophilic ones predominating. For example, in the three series, the S······H-N (DG4), S-H······O (DC21), S······H-N (DG22), and N-H······N (DG22) interactions were conserved in at least two or more complexes, i.e., N-H······O (DC23), N-H······O (DG24). On the other hand, the predominant hydrophobic interactions were *π*-alkyl ones (DG4, DA5, DA6, DC21, DG22, and DC23). It should be noted that some of these interactions have been found in other works where tin (IV) complexes exhibited binding affinities with DNA fragments [59, 62, 64]. On the other hand, the molecular interaction correlated with *in vitro* studies; these diorganotin(IV) derivatives have better values of IC_50_ compared to Cisplatin.

We also conducted molecular docking studies using HDOCK software (https://hdock.phys.hust.edu.cn/). The results are presented in Table S4. Based on the results, the binding energies of complexes **1a–1e**, **2a–2e**, and **3a–3e** (−92.53 to −110.64 kcal/mol) were significantly higher than the Cisplatin −50.63 kcal/mol. Autodock 4.2 software showed a similar trend with compounds **3d**, **3c**, **3e**, **1d**, **2e**, and **3a** exhibiting the strongest interaction energies, with binding energies of −110.64, −109.83, −109.78, −109.66, −105.82, and −105.74 kcal/mol, respectively. It is worth noting that both programs confirm that organotin(IV) complexes have a higher ability to interact with the DNA compared to Cisplatin and that most of the DNA nucleotides with which tin complexes interact are conserved. This validates the effectiveness of tin complexes in DNA interaction.

These findings indicated that such compounds could be proposed as possible anticancer agents because they inhibit the proliferation of various cancer cell lines and intercalate with DNA fragments. The other representations of tin (IV) complexes are described in Figure S111.

## 5. Conclusions

The synthesis of organotin(IV) complexes through a one-pot reaction using a three-component approach provides an efficient strategy to produce monomeric complexes in high yields. All organotin complexes evaluated exhibited higher cytotoxicity than Cisplatin on the two breast cancer cell lines. The organotin complexes were more cytotoxic against the triple-negative breast cancer cell line MDA-MB-23. The phenyl substituent on the nitrogen of the thiosemicarbazone moiety increased the cytotoxicity; the IC_50_ values suggest that the substituents bonded to the tin atom increased the cell growth inhibition, and the CLogP values indicate that the lipophilic-hydrophilic balance facilitated the permeability and absorption through the cell membrane. The selectivity index of the organotin(IV) complexes showed a higher selectivity than Cisplatin on both MCF-7 and MDA-MB-231 cancer cell lines compared to the normal cell line COS-7. Thus, the organotin(IV) complex may have a less toxic effect on normal cells than Cisplatin. The morphological alterations on both cancer cell lines suggested different stages of apoptosis. The fluorescence emission spectra of complexes **1a**, **1c**, and **1d** revealed the interaction of the organotin(IV) complexes with DNA, and the constant K_sv_ values suggested that the bis-trimethylsilyl methyl substituent on tin had a stronger interaction with the DNA, followed by the phenyl substituent which could be stacked inside the DNA. Thermodynamic parameters obtained by Isothermal Titration Calorimetry showed that the binding reaction **1c** with the DNA was exothermic. Molecular docking studies revealed that the organotin(IV) complexes were intercalated in the DNA; in general, most of the compounds showed better binding energy than the Cisplatin, particularly compounds with the phenyl ring on the nitrogen of thiosemicarbazone fraction showed a higher affinity with the dodecamer DNA. Such findings were correlated with the cytotoxicity results. The organotin(IV) complexes exhibited selective cytotoxicity and potency towards breast cancer cell lines, and thus, they could be used in promising applications in the design of anticancer agents.

## Figures and Tables

**Table 1:** Characteristic Fourier transform (FT) infrared (IR) vibrations (cm^−1^) of organotin(IV) complexes.

Compound	*ν* (OC=O)	*ν* (CO_pyrone_)	*ν* (C=N)	*ν* (Sn-N)	*ν* (Sn-S)	*ν* (Sn-O)	*ν* (Sn-C)
**1a**	1670	1332	1558	432	314	683	520
**1b**	1697	1354	1544	441	325	695	523
**1c**	1694	1354	1595	439	326	693	527
**1d**	1707	1350	1580	439	323	696	527
**1e**	1696	1320	1595	437	324	689	523
**2a**	1684	1355	1552	446	352	582	523
**2b**	1693	1353	1547	441	355	582	522
**2c**	1681	1352	1547	448	364	581	528
**2d**	1701	1353	1545	437	356	583	523
**2e**	1692	1355	1556	437	360	587	519
**3a**	1683	1353	1553	440	322	690	527
**3b**	1687	1353	1551	442	324	690	523
**3c**	1683	1352	1549	439	330	691	526
**3d**	1684	1352	1549	440	325	634	523
**3e**	1684	1352	1557	447	342	693	526

**Table 2:** ^119^Sn nuclear magnetic resonance (NMR) spectra, coupling constants, and C-Sn-C bond angle values in solution.

Compound	CDCl_3_-d	DMSO-d_6_	^1^ *J* (^13^C-^119^Sn)	*θ* (C-Sn-C)
**1a**	−129.2	−173.7	556.9	130.3
**1b**	−135.8	−173.2	550.4	129.7
**1c**	−239.9	−324.2	902.3	132.5
**1d**	−172.7	−184.6	532.0	123.4
**1e**	−118.0	−129.0	448.1	119.5
**2a**	−135.7	−177.1	564.5	131.2
**2b**	−135.8	−173.2	550.4	129.7
**2c**	−246.0	−326.8	1088.7	144.5
**2d**	−178.6	−189.4	535.8	123.7
**2e**	−125.4	−133.1	450.8	119.7
**3a**	−141.2	−197.5	554.4	130.1
**3b**	−141.4	−195.3	548.4	129.5
**3c**	−251.0	−347.6	916.6	133.4
**3d**	−184.7	−203.0	531.3	123.3
**3e**	−129.5	−142.0	447.8	119.4

**Table 3:** Selected bond distances (Å), angles (°), and torsion angles (°) for compounds **1a**, **3a**, **3c**, and **3d**.

Bond distances (Å)	**1a**	**3a**	**3c**	**3d**

Sn2-N6	2.218 (15)	2.193 (2)	2.194 (5)	2.193 (4)
Sn2-S3	2.533 (5)	2.563 (1)	2.507 (2)	2.531 (1)
Sn2-O1	2.276 (12)	2.160 (2)	2.140 (4)	2.168 (3)
Sn2-C15/C21	2.119 (2)	2.126 (4)	2.127 (6)	2.141 (5)
Sn2-C19/C25/C27	2.127 (19)	2.123 (3)	2.117 (7)	2.176 (5)
O2-C8	1.227 (2)	1.210 (4)	1.217 (8)	1.208 (6)
O9-C8	1.389 (2)	1.390 (4)	1.390 (8)	1.393 (6)
N5-C4	1.313 (2)	1.302 (4)	1.288 (6)	1.303 (6)
S(3)-C4	1.755 (18)	1.751 (3)	1.795 (5)	1.750 (5)
N14-C4	1.346 (2)	1.361 (4)	1.360 (7)	1.372 (6)
O1-C11A	1.291 (2)	1.295 (4)	1.290 (6)	1.2865 (5)

*Bond angles (°)*				
O1-Sn2-N6	76.3 (1)	79.1 (1)	80.3 (1)	78.1 (1)
O1-Sn2–S3	151.8 (3)	155.1 (1)	158.0 (1)	153.4 (1)
O1-Sn2-C15/C21	88.2 (1)	92.5 (1)	91.7 (2)	89.3 (2)
O1-Sn2-C19/C25/C27	84.0 (1)	87.9 (1)	87.8 (2)	90.0 (2)
S3-Sn2-N6	75.5 (4)	76.3 (1)	77.8 (1)	76.4 (1)
S3-Sn2-C15/C21	100.3 (1)	100.3 (1)	101.0 (2)	96.7 (2)
S3-Sn2-C19/C25/C27	102.3 (1)	99.2 (1)	100.3 (3)	105.8 (2)
N6-Sn2-C15/C21	107.4 (1)	113.8 (1)	109.9 (1)	119.0 (2)
N6-Sn2-C219C25/C27	103.1 (1)	114.2 (1)	117.9 (2)	110.6 (2)
C15/C21-Sn2-C19/C25/C27	145.7 (1)	131.2 (2)	122.2 (3)	129.0 (2)

*Torsion angles (°)*				
Sn2-O1-C11A-C7A	−45.08 (3)	41.11 (5)	7.98 (4)	35.99 (3)
Sn2-N6-C7-C7A	−0.68 (2)	0.42 (5)	39.12 (4)	7.65 (3)
Sn2-N6-N5-C4	35.31 (2)	27.91 (4)	24.32 (3)	26.50 (3)
Sn2-S3-C4-N5	−24.74 (6)	27.85 (3)	20.41 (3)	25.35 (2)

**Table 4:** IC_50_ (*µ*M) values of diorganotin complexes **1a–1e**, **2a–2e**, and **3a–3e**, selectivity index (SI), and calculated coefficient (CLogP), and percent inhibition of **L1a–L1c** at 20 (*µ*M).

Complex	MCF-7^*∗*^	MDA-MB-231^*∗*^	COS-7^*∗*^	SI		CLogP
				MCF-7	MDA-MB-231	
**1a**	0.380 ± 0.030	0.12 ± 0.0100	0.49 ± 0.030	1.23	4.9	1.88
**1b**	8.650 ± 0.700	11.05 ± 1.500	12.17 ± 0.300	1.40	1.10	5.92
**1c**	0.611 ± 0.020	0.170 ± 0.010	0.68 ± 0.050	1.11	4.00	2.40
**1d**	0.203 ± 0.008	0.119 ± 0.003	0.18 ± 0.006	0.16	1.51	2.82
**1e**	0.180 ± 0.005	0.042 ± 0.008	0.14 ± 0.010	0.77	3.33	3.61
**2a**	0.21 ± 0.040	0.31 ± 0.020	0.62 ± 0.060	2.95	2.00	2.26
**2b**	1.39 ± 0.030	3.28 ± 0.080	11.58 ± 0.800	8.33	2.53	6.30
**2c**	1.08 ± 0.010	1.02 ± 0.030	1.15 ± 0.100	1.06	1.13	2.78
**2d**	0.20 ± 0.002	0.087 ± 0.200	0.28 ± 0.010	1.40	3.21	3.19
**2e**	0.15 ± 0.020	0.12 ± 0.008	0.29 ± 0.030	1.93	2.41	3.98
**3a**	0.21 ± 0.009	0.10 ± 0.010	0.34 ± 0.010	1.61	3.40	3.95
**3b**	0.73 ± 0.100	0.54 ± 0.080	1.60 ± 0.200	2.19	2.96	8.0
**3c**	0.16 ± 0.010	0.49 ± 0.002	0.25 ± 0.006	1.56	0.51	4.47
**3d**	0.16 ± 0.006	0.07 ± 0.005	0.16 ± 0.020	1.00	0.30	4.89
**3e**	0.04 ± 0.002	0.14 ± 0.003	0.25 ± 0.010	6.25	1.79	5.68
Cisplatin	5.30 ± 0.50	27.7 ± −2.90	7.17 ± 0.600	1.35	0.26	

Ligand	% Inhibition at 20 (*µ*M)		COS-7			
	MCF-7	MDA-MB-231				

**L1a**	7.0	NC	NC	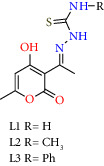		
**L1b**	3.6	NC	NC			
**L1c**	48.8	17.6	25.5			

^*∗*^Data represent the average of three or four independent assays and are expressed as the mean ± standard error (SE), NC: noncytotoxic at 20 (*µ*M).

**Table 5:** Thermodynamic parameters of affinity assay of compound **1c** with ss-DNA.

Temperature (°C)	*K*_*b*_ (M)	Δ*G*_*b*_ (kcal·mol^−1^)	Δ*H*_*b*_ (kcal·mol^−1^)	−TΔ*S*_*b*_ (kcal·mol^−1^)
25	4.5 × 10^4^ ± 5.35 ×10^3^	−6.3474	−3.775 ± 0.272	−2.573

**Table 6:** Docking results of diorganotin (IV) derivatives **1a–**1**e**, **2a–**2**e**, and **3a–**3**e**, and Cisplatin with A B-DNA dodecamer.

Compound	Binding energy Δ*G* (kcal/mol)	DNA residues interacting with the ligand	Polar interactions	Hydrophobic interactions
***Cis*-platin**	−7.18	DC21	**N**-H·····O (DC21)	*π * **–alkyl** DG22

**1a**	−7.57	DG4, DA6, DC21, DG22, DC23	S······H-N (DG4) S-H·····O (DC21) S·····H-N (DG22) N-H·····N (DG22) N-H·····O (DC23)	*π * **-alkyl** DA6

**1b**	−7.07	DG4, DA6, DT20, DC21, DG22, DC23	S······H-N (DG4) S-H······O (DC21) S······H-N (DG22) N-H······N (DG22) N-H······O (DC23)	*π * **-alkyl** DA6, DT20

**1c**	−8.46	DC3, DG4, DA5	N-H······O (DC3) N-H······O (DG4) S-H······O (DA5)	—

**1d**	−9.23	DG4, DC23, DG24	S-H······O (DC23) N-H······O (DC23) N-H······O (DG24)	*π * **-alkyl** DG4

**1e**	−8.24	DG22, DC23, DG24	S······H-N (DG22) N-H······O (DC23) N-H······O (DG24)	—

**2a**	−7.91	DG4, DA5, DA6, DC21, DG22, DC23	O······H-N (DG4) O······H-C (DC23)	*π * **-alkyl** DA5, DA6, DC21, DG22, DC23

**2b**	−7.51	DG22, DC23, DG24	N-H······C (DC23) N-H······C (DG24) N-H······O (DG24)	*π * **-alkyl** DG22, DC23

**2c**	−8.68	DG4, DG24	N-H······O (DG4) O······H-C (DG24)	—

**2d**	−9.07	DA5, DG22, DC23	O······H-C (DA5) S······H-N (DG22) N-H······O (DC23) S-H······O (DC23)	—

**2e**	−8.98	DG4, DA5, DC21, DG22, DC23	S······H-N (DA5) C-H······O (DC23)	*π * **-alkyl** DG22

**3a**	−8.86	DG4, DA5, DA6, DC23	N-H······N (DG4) N-H······N (DA5) S······H-N (DA5) S-H······O (DA6)	*π * **- ** *π * **T-shaped** DG4 *π ***-anion** DC23

**3b**	−8.11	DG4, DA5, DG22	N-H······N (DA5) S-H······O (DG22)	*π * **-alkyl** DG4, DG22

**3c**	−9.92	DA5, DC21	S-H······O (DA5) N-H······O (DC21)	—

**3d**	−10.12	DA6, DG22, DC23	N-H······O (DA6)	*π * **-alkyl** DG22, DC23

**3e**	−9.29	DC3, DG4, DA5, DG24	N-H······O (DC3) S-H······O (DG4) N-H······O (DG4) S-H······O (DA5) O······H-C (DG24)	*π * **-sigma** DC3 *π*-*π*T-shaped DC3

The bold values correspond to intermolecular hydrophobic interactions between the organotin complexes and DNA nucleotides.

## Data Availability

The article includes the data supporting the findings of this study; further information can be obtained from Supplementary Materials.

## Supplementary Materials

Supplementary Materials Table S1: Crystallographic and structure refinement data for compounds **1a**, **3a**, **3c**, and **3d;** Table S2: Selected intermolecular interactions (Å,°) for compounds **1a**, **3a**, **3c**, and **3d**; Table S3: Noncytotoxic concentration (*µ*M) on normal cell line COS-7of complexes **1a**–**3e**; Table S4: Molecular docking studies using HDOCK; Figures S1: Ultraviolet-visible (UV-Vis) spectra in CH_2_Cl_2_ of complexes **1a**–**3e;** S2–S16: FT-IR spectra of complexes **1a**–**3e**; Figures S17–S35: Mass spectrometry (DART^+^) and (IE^+^) spectra of complexes **1a**–**3e**; Figure S36–S50: ^1^H NMR spectra of complexes **1a**–**3e**; Figures S51–S65: ^13^C NMR spectra of complexes **1a**–**3e**; Figure S66–S73: 2D NMR spectra (HSQC and HMBC) of complexes **1a**–**3e**; Figures S74-S103: ^119^Sn NMR spectra of complexes **1a**–**3e**; Figure S104: UV-Vis in DMSO-Tris-HCl buffer A) 0 h, B) 72 h; Figure S105–110: ^1^H NMR spectra in DMSO at 0, 24, 56, and 72 h; Figure S111: Representation of the interactions between A B-DNA dodecamer and diorganotin(IV) derivatives **1a**–**1e**, **2a**–**2e**, and **3a-3b**.
