# Supplementary material for: New Organotin (IV) Compounds Derived from Dehydroacetic Acid and Thiosemicarbazides: Synthesis, Rational Design, Cytotoxic Evaluation, and Molecular Docking Simulation
Source: Bioinorg Chem Appl. 2023 Oct 25;2023:7901843. doi: 10.1155/2023/7901843 (PMC10620030; doi:10.1155/2023/7901843)
Supplement: Supplementary Materials — Table S1: Crystallographic and structure refinement data for compounds 1a, 3a, 3c, and 3d; Table S2: Selected intermolecular interactions (Å,°) for compounds 1a, 3a, 3c, and 3d; Table S3: Noncytotoxic concentration (µM) on normal cell line COS-7of complexes 1a–3e; Table S4: Molecular docking studies using HDOCK; Figures S1: Ultraviolet-visible (UV-Vis) spectra in CH2Cl2 of complexes 1a–3e; S2–S16: FT-IR spectra of complexes 1a–3e; Figures S17–S35: Mass spectrometry (DART+) and (IE+) spectra of complexes 1a–3e; Figure S36–S50: 1H NMR spectra of complexes 1a–3e; Figures S51–S65: 13C NMR spectra of complexes 1a–3e; Figure S66–S73: 2D NMR spectra (HSQC and HMBC) of complexes 1a–3e; Figures S74-S103: 119Sn NMR spectra of complexes 1a–3e; Figure S104: UV-Vis in DMSO-Tris-HCl buffer A) 0 h, B) 72 h; Figure S105–110: 1H NMR spectra in DMSO at 0, 24, 56, and 72 h; Figure S111: Representation of the interactions between A B-DNA dodecamer and diorganotin(IV) derivatives 1a–1e, 2a–2e, and 3a-3b. [file 7901843.f1.docx]

Supporting Material

**New organotin (IV) compounds derived from dehydroacetic acid and thiosemicarbazides: Synthesis, rational design, cytotoxic evaluation, and molecular docking simulation.**

Elizabeth Gómez^1^, José Miguel Galván-Hidalgo^1^, Guillermo Pérez-Cuéllar^1^, Karoline Alondra Huerta-Landa^1^, Arturo González-Hernández^1^, Jorge Omar Gómez-García^2^, Dulce Andrade-Pavón^3,4^ ,Teresa Ramírez-Apan^1^, Karla Daniela Rodríguez Hernández^1^, Simón Hernández^1^ Patricia Cano-Sánchez^1^, and Homero Gómez-Velasco^1^.

*^1^Instituto de Química, Universidad Nacional Autónoma de México, Circuito exteriori S/N, Ciudad Universitaria, Alcaldía Coyoacán, C. P. 04510, Ciudad de México, México.*

*^2^ Departamento Química Orgánica, Escuela Nacional de Ciencias Biológicas-IPN, Prolongación de Carpio y Plan de Ayala S/N; Colonia Santo Tomás, 11340, Ciudad de México, México.*

*^3^ Departamento Microbiología, Escuela Nacional de Ciencias Biológicas, Instituto Politécnico Nacional, Prolongación de Carpio y Plan de Ayala S/N; Colonia Santo Tomás, 11340, Ciudad de México, México.*

*^4^Departamento Fisiología, Escuela Nacional de Ciencias Biológicas, Instituto Politécnico Nacional, Av. Wilfrido Massieu 399, Colonia Nueva Industrial Vallejo, 07738, Ciudad de México, México.*

**Table S1**. Crystallographic and structure refinement data for compounds **1a**, **3a**, **3c** and **3d**

| Identification code | **1a** | **3a** | **3c** | **3d** |
| --- | --- | --- | --- | --- |
| Empirical formula | C_17_H_27_N_3_O_3_SSn | C_23_H_31_N_3_O_3_SSn | C_27_H_23_N_3_O_3_SSn | C_27_H_35_N_3_O_3_SSn_3_ |
| Formula weight | 472.16 | 548.26 | 588.23 | 600.33 |
| Temperature/K | 298(2) | 298(2) | 298(2) | 250(2) |
| Crystal system | orthorhombic | Monoclinic | monoclinic | orthorhombic |
| Space group | Pbcn | P2_1_/c | Cc | P2_1_2_1_2_1_ |
| a/Å | 15.8383(5) | 7.9587(3) | 15.2998(5) | 13.0234(4) |
| b/Å | 15.7893(5) | 18.0150(9) | 12.6384(4) | 13.9917(4) |
| c/ Å | 16.5875(6) | 18.2614(7) | 14.0421(5) | 14.8767(4) |
| α/° | 90 | 90 | 90 | 90 |
| β/° | 90 | 95.8390(10) | 102.8274(10) | 90 |
| γ/° | 90 | 90 | 90 | 90 |
| Volume/ Å | 4118.1(2) | 2610.26(19) | 2647.49(15) | 2710.82(14) |
| Z | 8 | 4 | 4 | 4 |
| ρcalg/cm^3^ | 1.512 | 1.395 | 1.476 | 1.471 |
| μ/mm^-1^ | 1.352 | 1.085 | 1.076 | 1.052 |
| F(000) | 1920 | 1120 | 1184 | 12.32 |
| Crystal size/mm^3^ | 0.37 x 0.29 x 0.21 | 0.33 x 0.32 x 0.29 | 0.36 x 0.29 x 0.16 | 0.36 x 0.32 x 0.31 |
| Radiation | MoKα (λ= 0.71073) | MoKα (λ= 0.71073) | MoKα (λ= 0.71073) | MoKα (λ= 0.71073) |
| 2Ɵ range for data collection | 2.197 to 25.388 | 2.242 to 25.378 | 2.402 to 25.404 | 2.538 to 25.383 |
| Reflections collected | 26360 | 16255 | 16474 | 26757 |
| Independent reflections | 3816[R_int_ = 0.0318] | 4774[R_int_ = 0.0343] | 4801[R_int_ = 0.0295] | 4978[R_int_ = 0.0243] |
| Data/restraints/parameters | 3816/42/265 | 4774/1/287 | 4801/189/367 | 4978/1/321 |
| Goodness-of-fit on F^2^ | 1.037 | 1.106 | 1.127 | 1.110 |
| Final R indexes [I≥2σ(I)] | R_1_ = 0.0192,  wR_2_ = 0.0454 | R_1_ = 0.0365,  wR_2_ = 0.0719 | R_1_ = 0.0310,  wR_2_ = 0.0570 | R_1_ = 0.0289,  wR_2_ = 0.0638 |
| Final R indexes [all data] | R_1_ = 0.0225,  wR_2_ = 0.0474 | R_1_ = 0.0484,  wR_2_ = 0.0777 | R_1_ = 0.0372  wR_2_ = 0.0596 | R_1_ = 0.0310,  wR_2_ = 0.0654 |

**Table S2**. Selected intermolecular interactions (Å,°) for compounds **1a**, **3a**, **3c,** and **3d**.

| **Compound** | **D-H···A** | **Distances (Å)**  **H···A** | **Distances (Å)**  **D···A** | **Angle (°)**  **D-H···A** | **Symmetry operation** |
| --- | --- | --- | --- | --- | --- |
|  | N(14)-H(14A)···N(5) | 2.292 | 3.108 | 159 | -x+1,y-z+1/2 |
| **1a** | N(14)-H(14B)···O(1) | 2.219 | 3.038 | 166 | x,-y+1, z-1/2 |
|  | C(11)-H(11)···S(3) | 2.753 | 3.693 | 170 | x,1-y,-1/2+z |
|  | C(8)-O(2)···Sn(2) | 2.761 | 3.662 | 129 | 1/2-x,-1/2+y,z |
|  |  |  |  |  |  |
|  | N(14)-H(14)···O(2) | 2.204 | 3.089 | 173 | x,-y+3/2, z+1/2 |
| **3a** | N(14)-H(14)···O(9) | 2.611 | 3.227 | 127 | x,-y+3/2, z+1/2 |
|  | C(19)-H(19)···O(2) | 2.626 | 3.429 | 145 | 2-x,-1/2+y, 1.5-z |
|  | C(12)-H(12A)···Cg(1) | 2.991 | 3.697 | 131 | -1+x,1.5-y,-1/2+z |
|  |  |  |  |  |  |
| **3c** | N(14)-H(14)···O(2) | 2.081 | 2.946 | 162 | x-1/2,- y+3/2, z-1/2 |
|  | C(20)-H(20)···O(2) | 2.581 | 3.356 | 141 | x-1/2,- y+3/2, z-1/2 |
|  | C(24)-H(24)···Cg(1) | 3.274 | 3.842 | 135 | 1/2+x,2.5-y,1/2+z |
|  |  |  |  |  |  |
|  | N(14)-H(14)···O(2) | 2.308 | 3.153 | 157 | 1/2-x,1- y,1/2+z |
| **3d** | C(16)-H(16)···O(2) | 2.636 | 3.425 | 142 | 1/2-x,1- y,1/2+z |
|  | C(25)-H(25A)···Cg(1) | 3.084 | 4.010 | 158 | 1/2-x,1- y,1/2+z |
|  | C(17)-H(17)···S(3) | 2.956 | 3.738 | 141 | -x,-1/2+ y,1.5-z |

Cg(1): (C15-C20)

**Table S3**. Non-Cytotoxic concentration (µM) on normal cell line COS-7 of Organotin(IV) complexes **1a-3e**.

| Complex | 1a | 1b | 1c | 1d | 1e | 2a | 2b | 2c | 2d | 2e | 3a | 3b | 3c | 3d | 3e |
| --- | --- | --- | --- | --- | --- | --- | --- | --- | --- | --- | --- | --- | --- | --- | --- |
| COS-7 (µM) | 0.02 | 0.2 | 0.02 | 0.01 | 0.01 | 0.01 | 0.2 | 0.02 | 0.01 | 0.01 | 0.02 | 0.01 | 0.01 | 0.01 | 0.01 |

**Table S4**. Docking results of diorganotin (IV) derivatives **1a**-**e**, **2a**-**e**, **3a**-**e**, and cisplatin with A B-DNA dodecamer, using HDOCK server.

| **Compound** | **Binding energy ΔG (kcal/mol)** | **DNA residues interacting with**  **the ligand** |
| --- | --- | --- |
| **cisplatin** | -50.63 | DC1, DG2, DC3, DG4, DC21, DG22, DC23 |
| **1a** | -104.82 | DG2, DC3, DG4, DA5, DA6, DT19, DT20, DC21, DG22 |
| **1b** | -103.35 | DG2, DC3, DG4, DA5 DT20, DC21, DG22 |
| **1c** | -108.73 | DG2, DC3, DG4, DA5, DT19, DT20, DC21, DG22 |
| **1d** | -109.66 | DC1, DG2, DC3, DG4, DT20,DC21, DG22, DC23 |
| **1e** | -103.83 | DC1, DG2, DC3, DT20, DC21, DG22, DC23 |
| **2a** | -105.50 | DC1, DG2, DC3, DG4, DA5, DT20, DC21, DG22 |
| **2b** | -105.42 | DC1, DG2, DC3, DT20, DC21, DG22, DC23 |
| **2c** | -105.71 | DG4, DA5, DA6, DT7, DT8, DG16, DA17, DA18, DA19, DG24 |
| **2d** | -92.53 | DC1, DG2, DC3, DG4, DA5, DT19, DT20, DC21, DG22 |
| **2e** | -105.82 | DG2, DC3, DG4, DA5, DT19, DT20, DC21, DG22 |
| **3a** | -105.74 | DG4, DA5, DA6, DT7, DG16, DA17, DA18, DT19, DT20 |
| **3b** | -104.04 | DC1, DG2, DC3, DG4, DA5, DT19, DT20, DC21, DG22, DC23 |
| **3c** | -109.83 | DC1, DG2, DC3, DG4, DA5, DT19, DT20, DC21, DG22 |
| **3d** | -110.64 | DC1, DG2, DC3, DG4, DA5, DA6, DT19, DT20, DC21, DG22 |
| **3e** | -109.78 | DC3, DG4, DA5, DT19, DT20, DC21, DG22 |


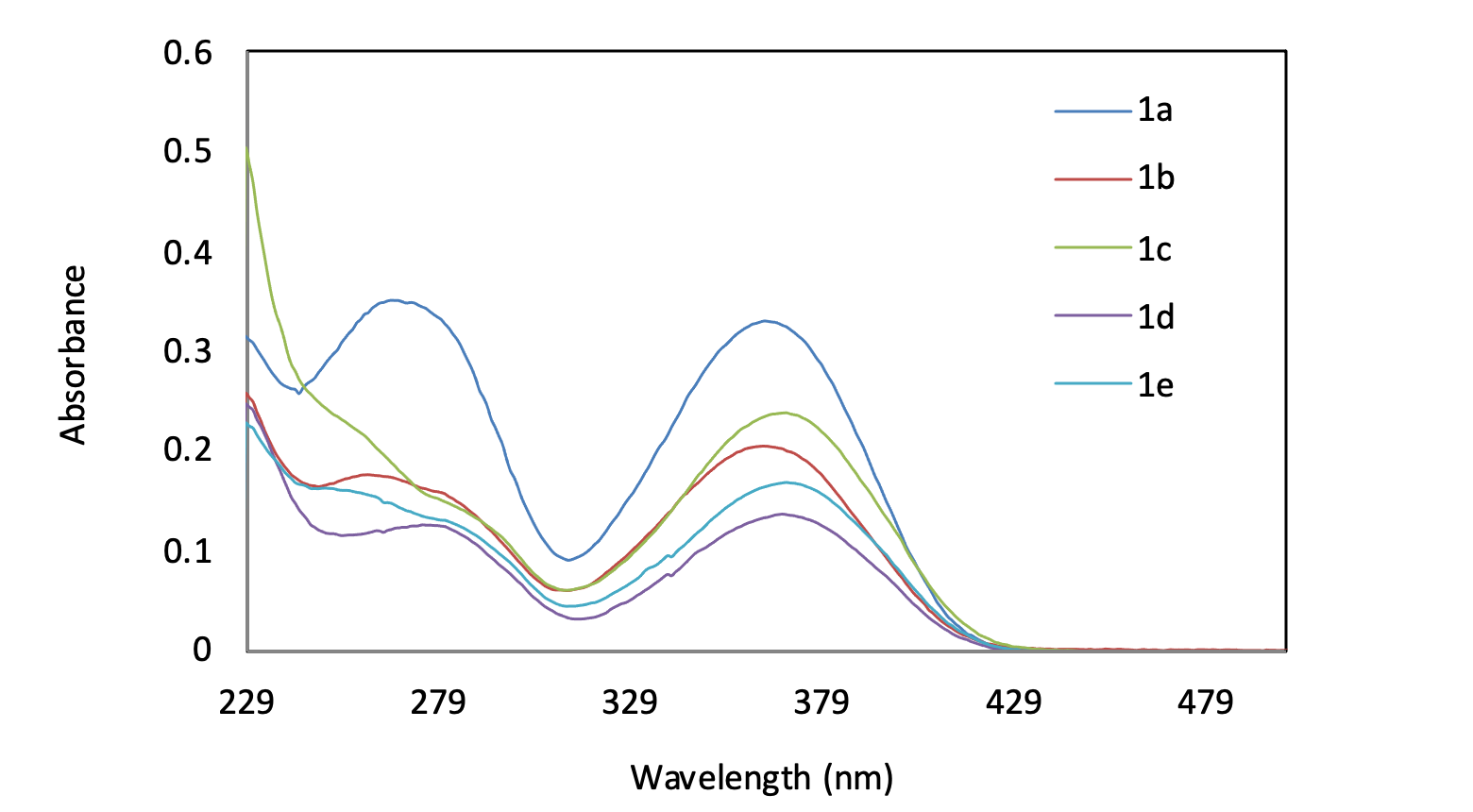


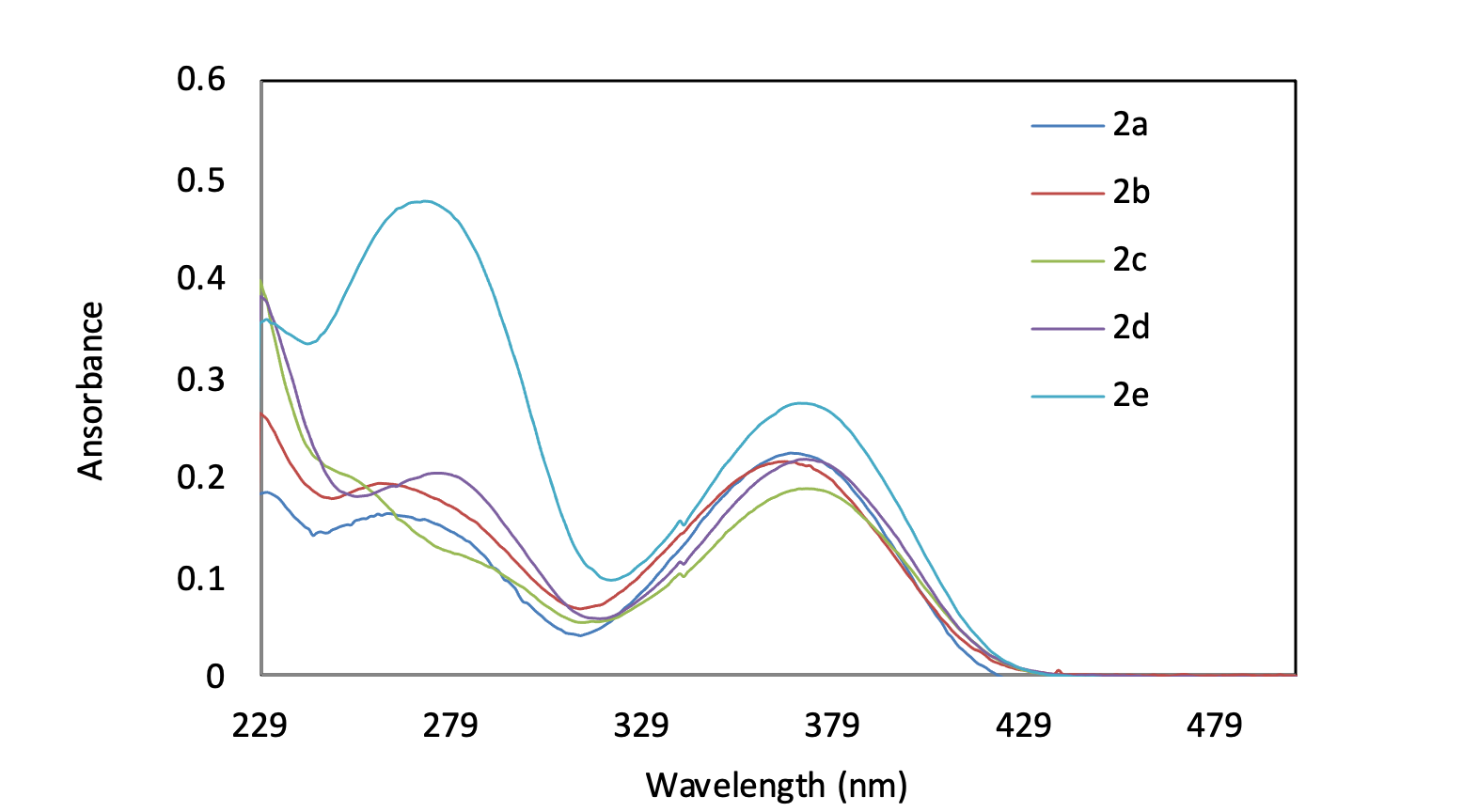


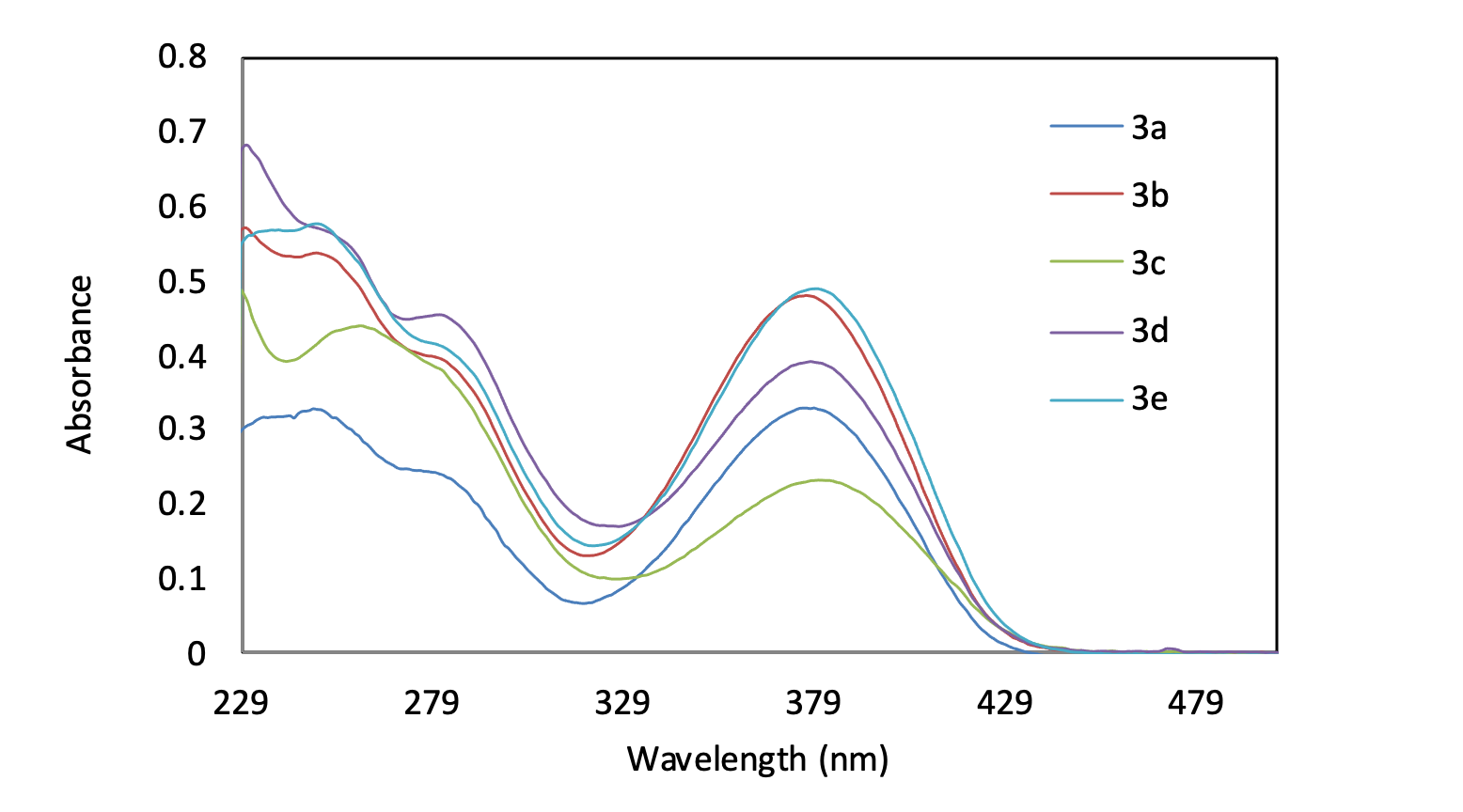


**Figure S1.** Ultraviolet-visible (UV-Vis) spectra in CH_2_Cl_2_ of diorganotin(IV) complexes

(2.04530 x10^-3^ M).


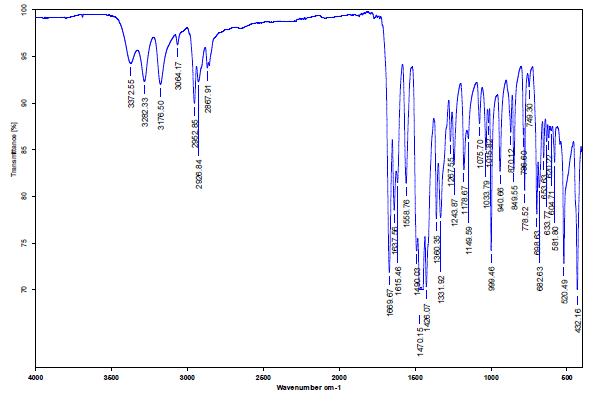


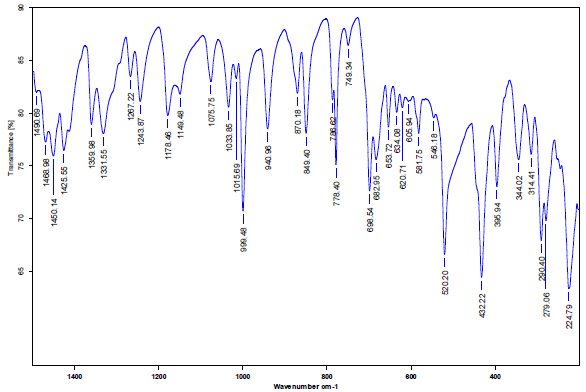


Figure S2. ATR-FTIR of compound **1a**.


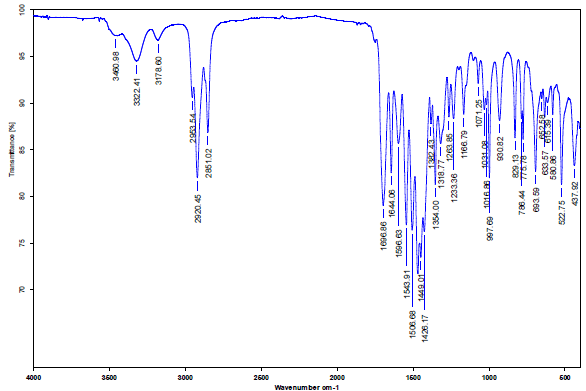


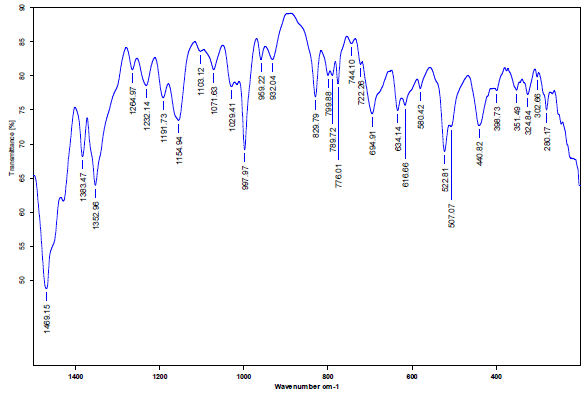


Figure S3. ATR-FTIR of compound **1b**.


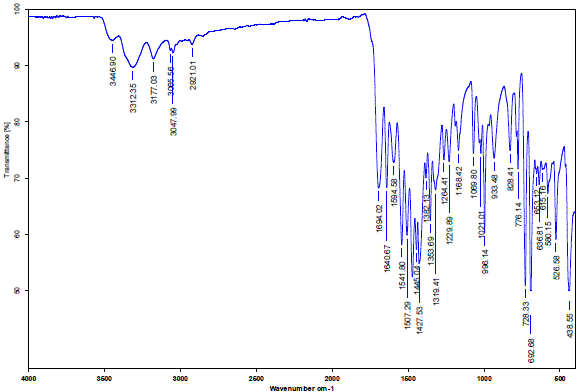


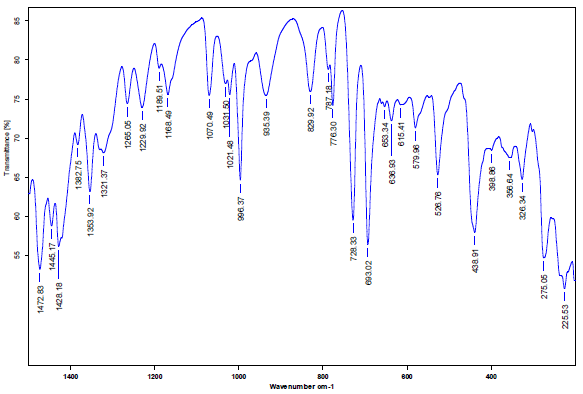


Figure S4. ATR-FTIR of compound **1c**.


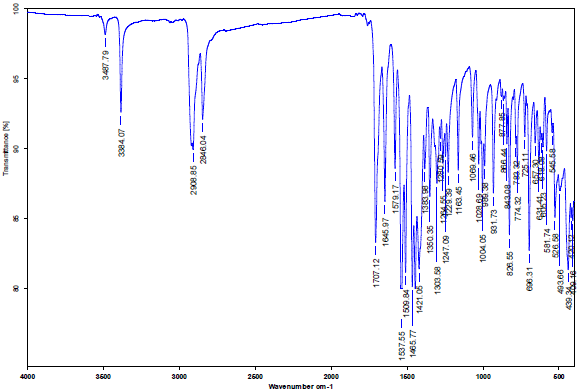


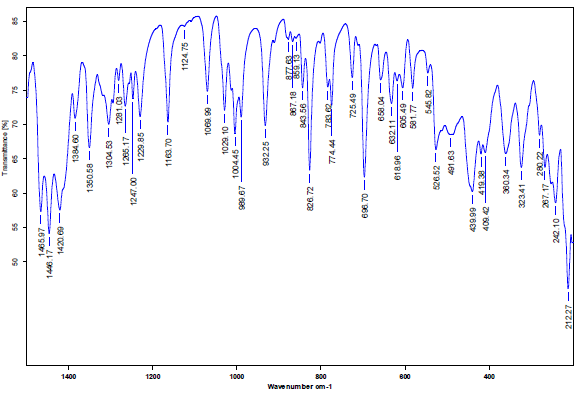


Figure S5. ATR-FTIR of compound **1d**.


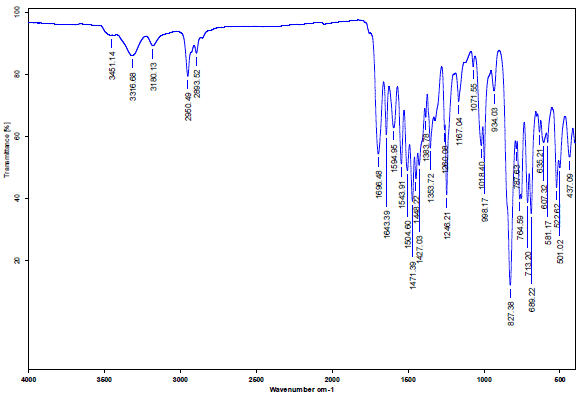


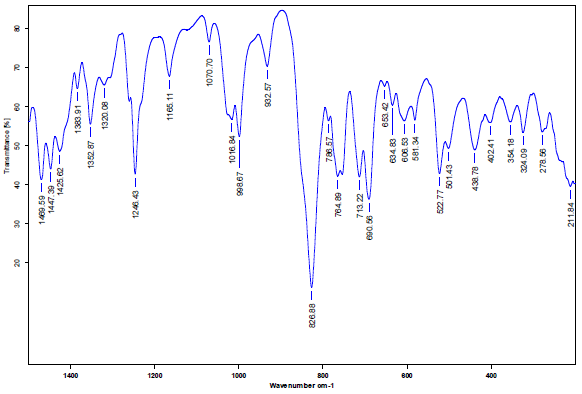


Figure S6. ATR-FTIR of compound **1e**.


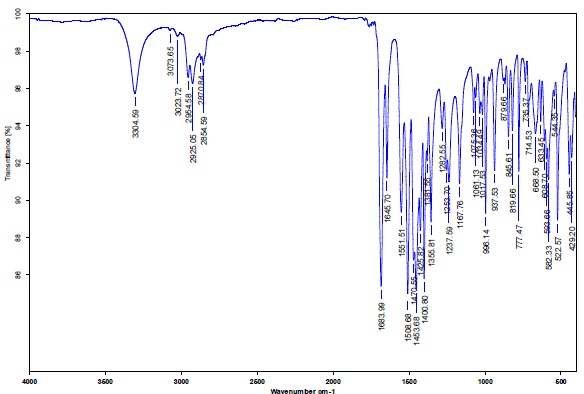


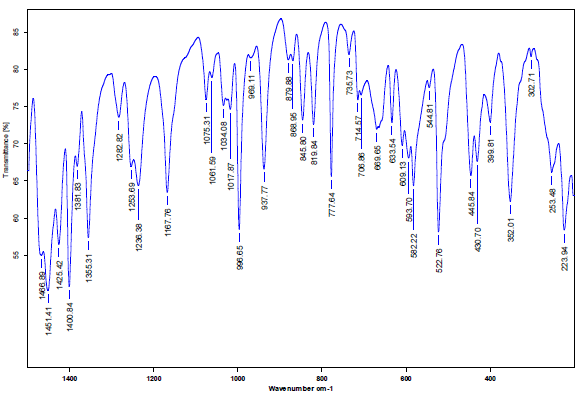


Figure S7. ATR-FTIR of compound **2a**.


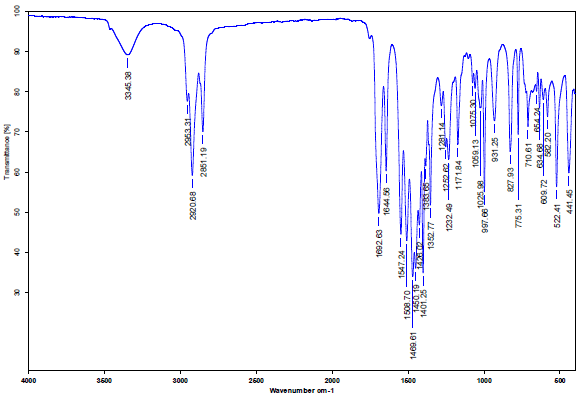


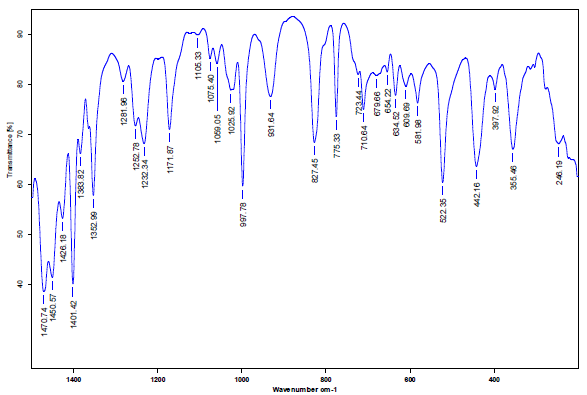


Figure S8. ATR-FTIR of compound **2b**.


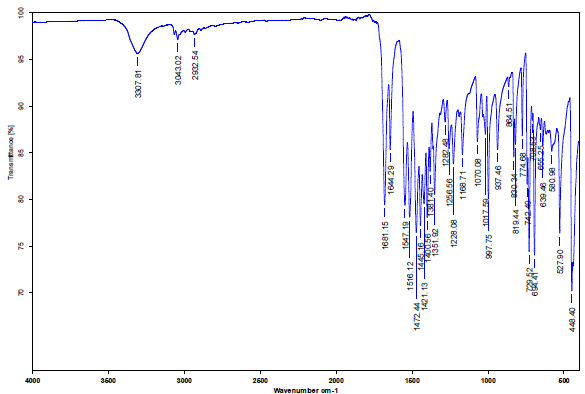


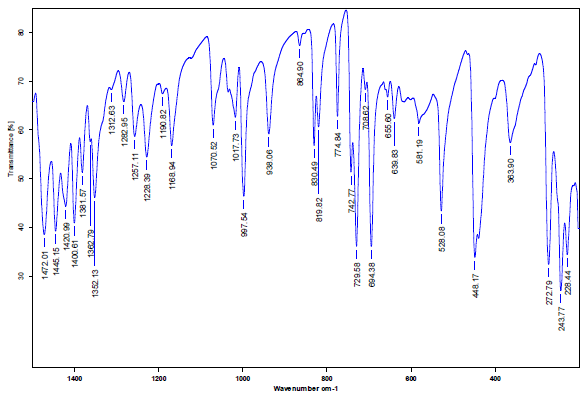


Figure S9. ATR-FTIR of compound **2c**.


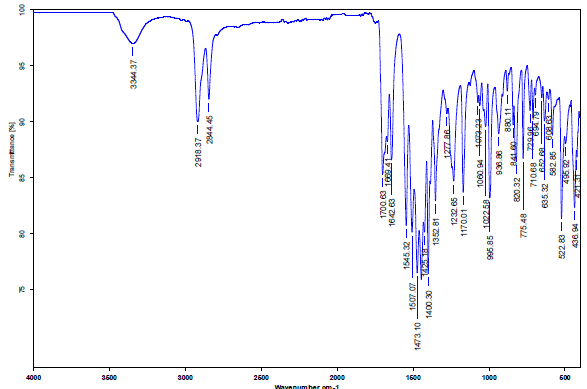


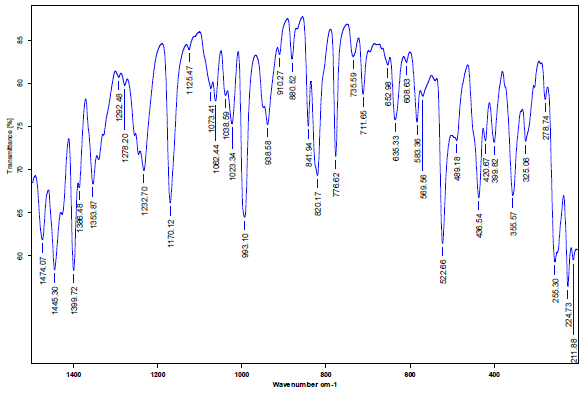


Figure S10. ATR-FTIR of compound **2d**.


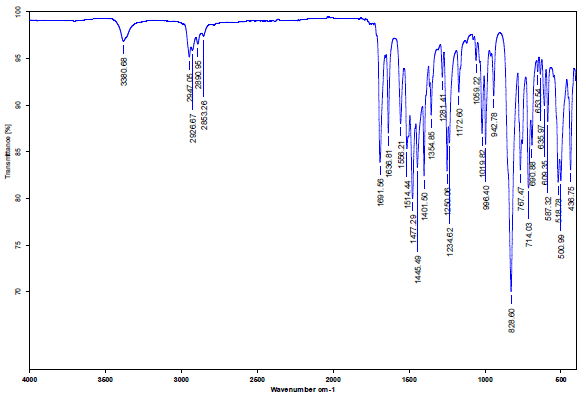


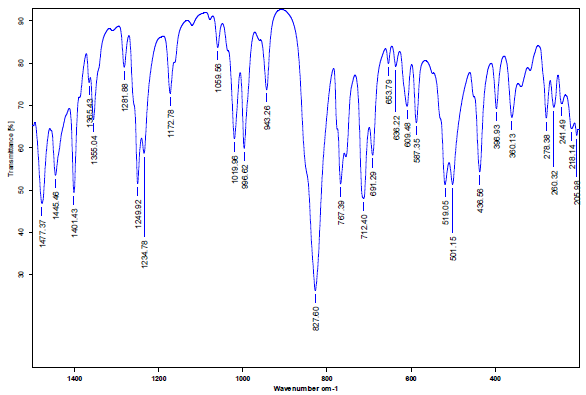


Figure S11. ATR-FTIR of compound **2e**.


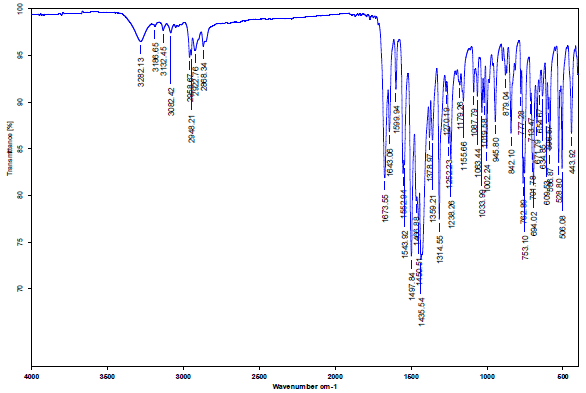


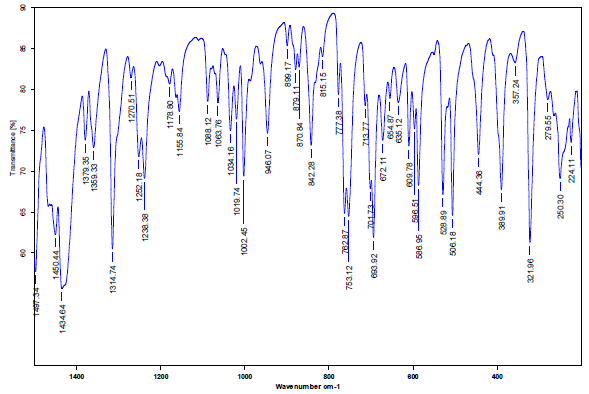


Figure S12. ATR-FTIR of compound **3a**.


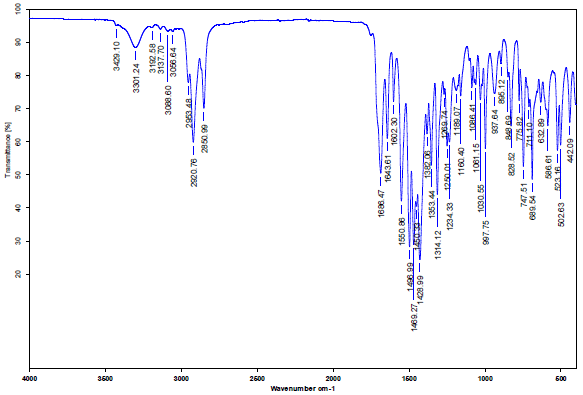


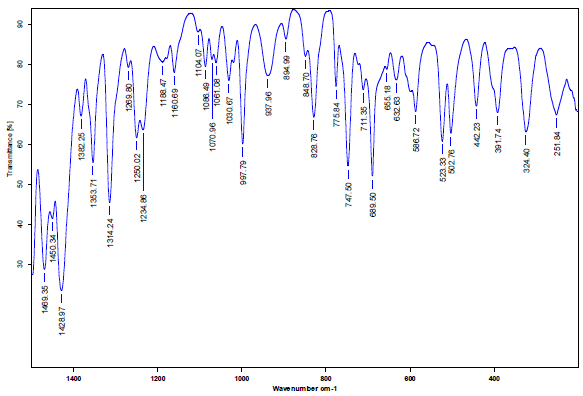


Figure S13. ATR-FTIR of compound **3b**.


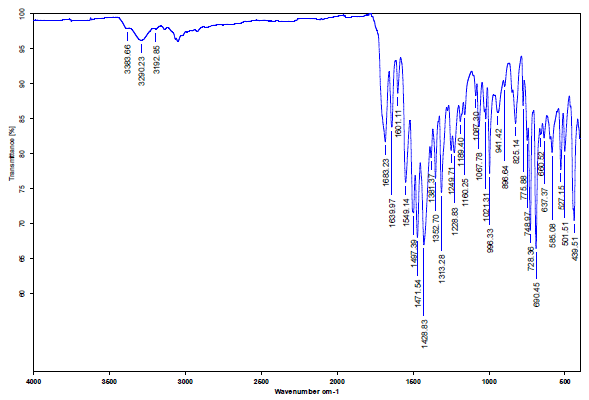


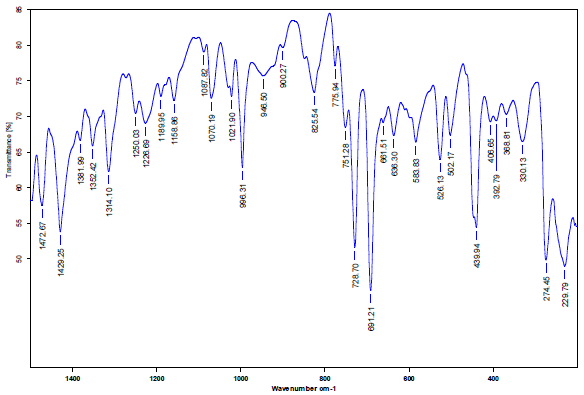


Figure S14. ATR-FTIR of compound **3c**.


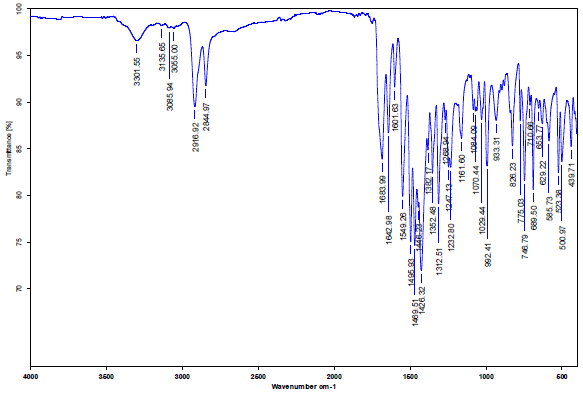


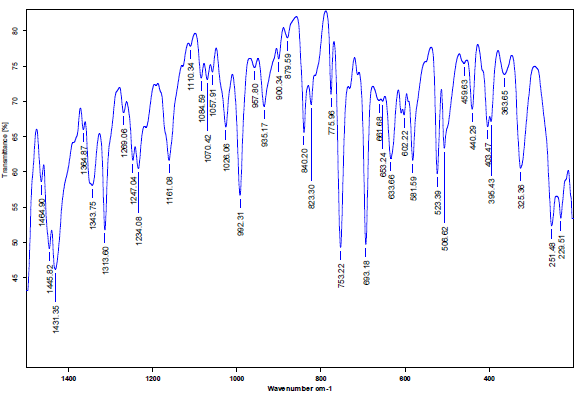


Figure S15. ATR-FTIR of compound **3d**.


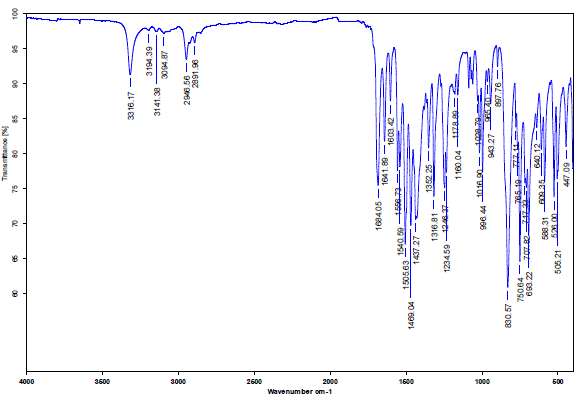


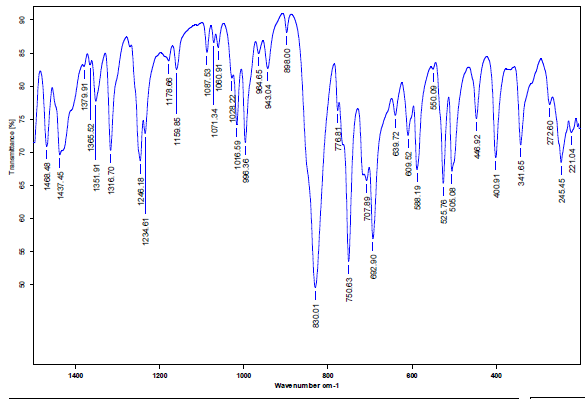


Figure S16. ATR-FTIR of compound **3e**.

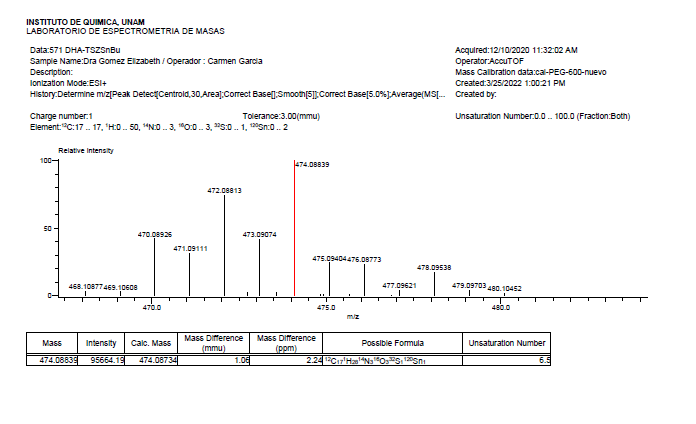


Figure S17. Mass spectrometry (EI) of compound **1a**


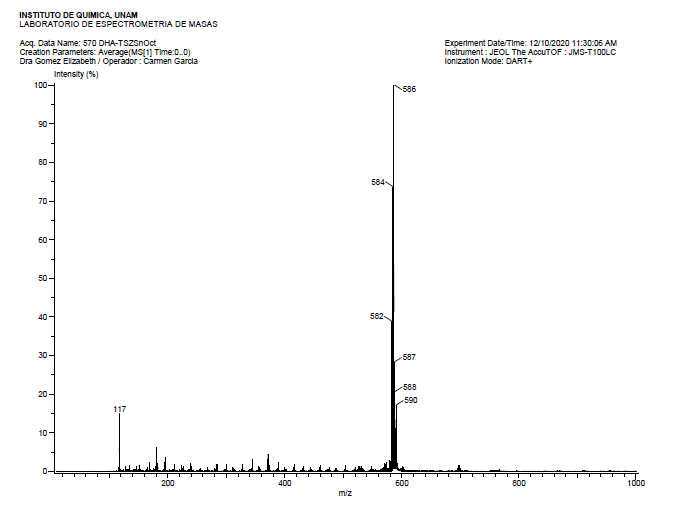


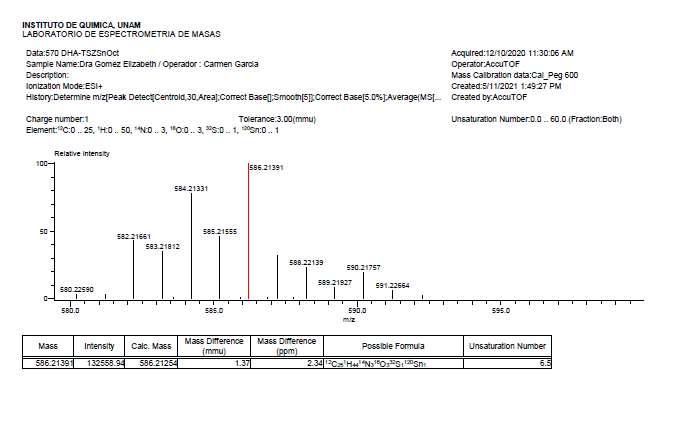


Figure S18. Mass spectrometry (DART) of compound **1b**.

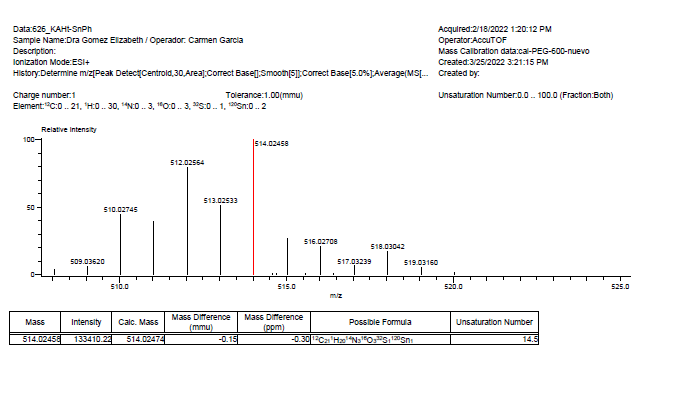


Figure S19. Mass spectrometry (EI) of compound **1c**.

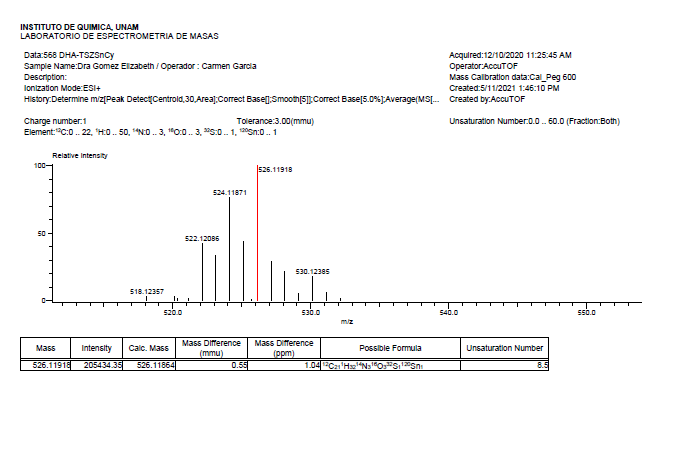


Figure S20. Mass spectrometry (EI) of compound **1d**.

Figure S21. Mass spectrometry (EI) of compound **1e**.


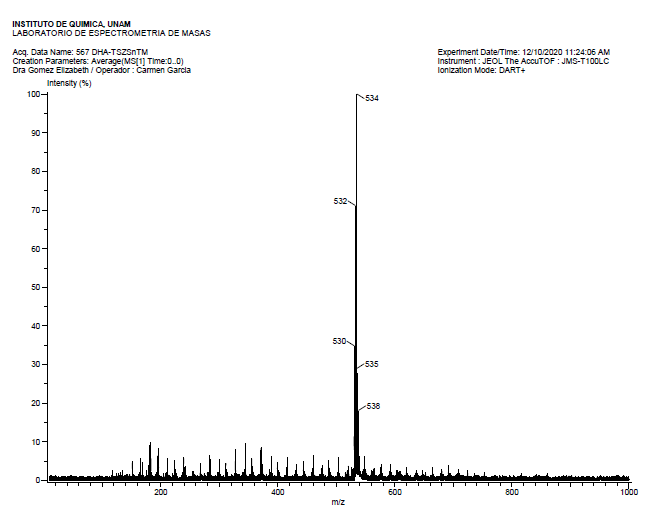


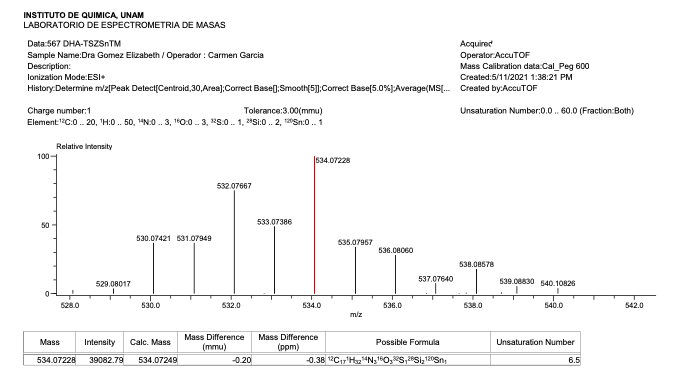


Figure S22. Mass Spectrometry (DART) of compound **1e.**

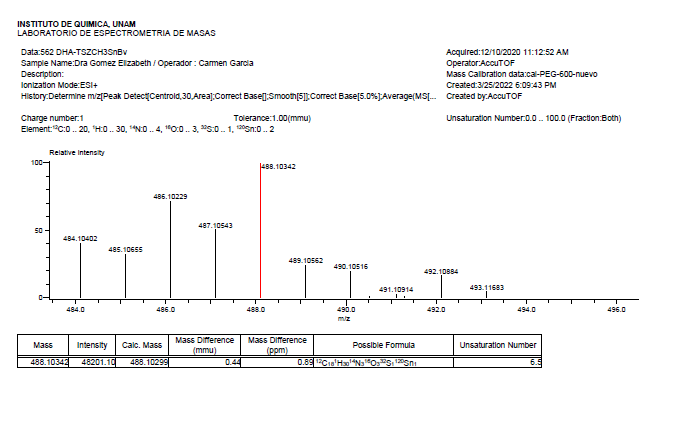


Figure S23. Mass Spectrometry (EI) of compound **2a.**


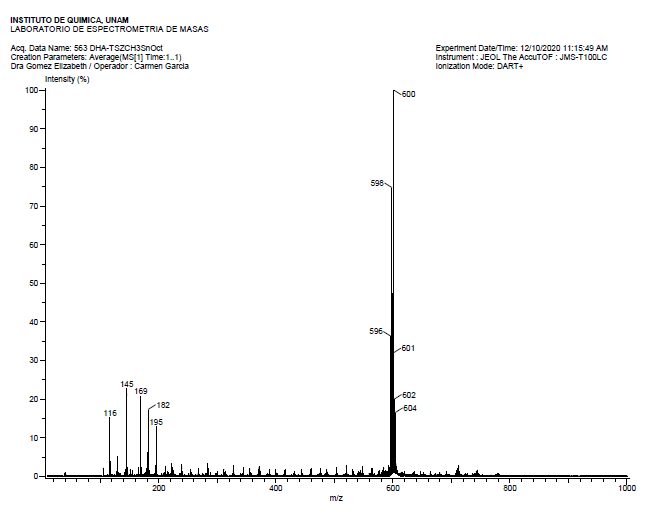


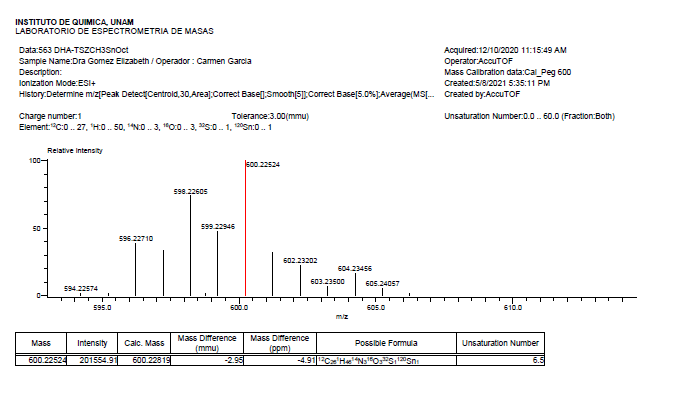


Figure S24. Mass Spectrometry (DART) of compound **2b.**


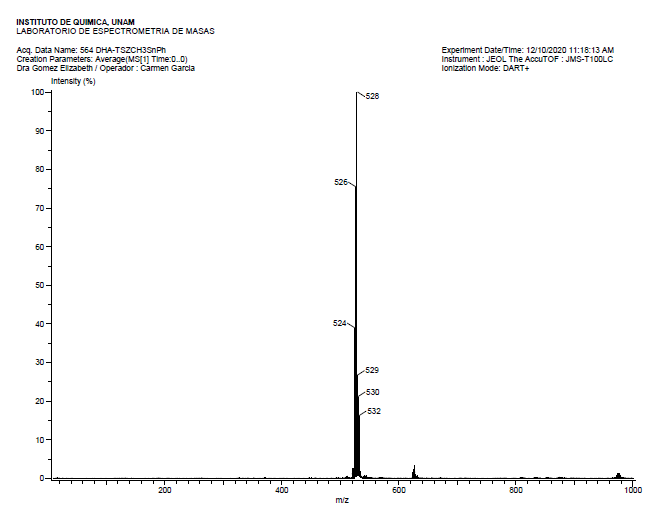


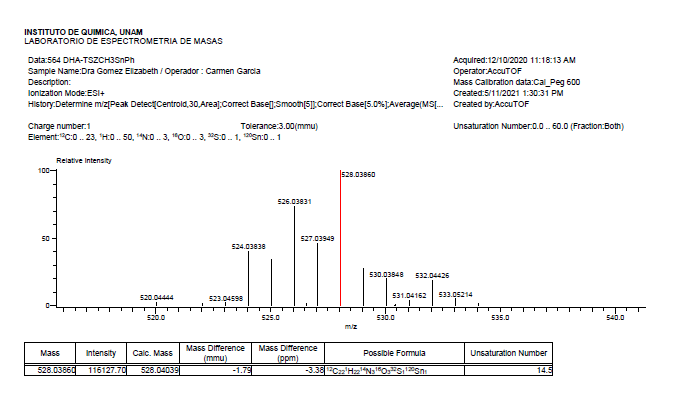


Figure S25. Mass Spectrometry (DART) of compound **2c.**


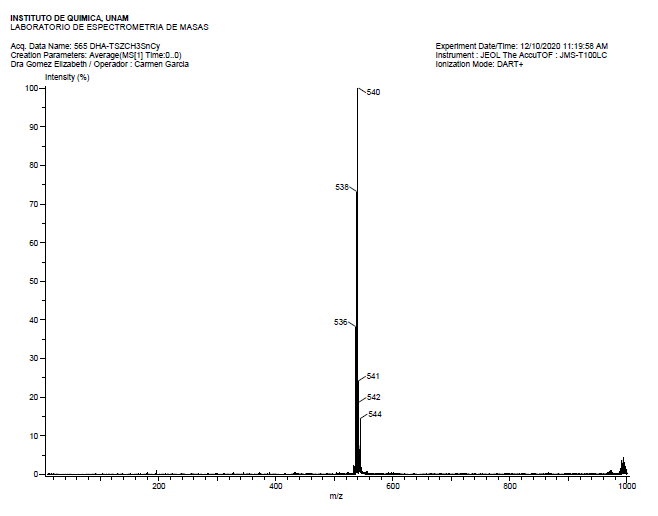


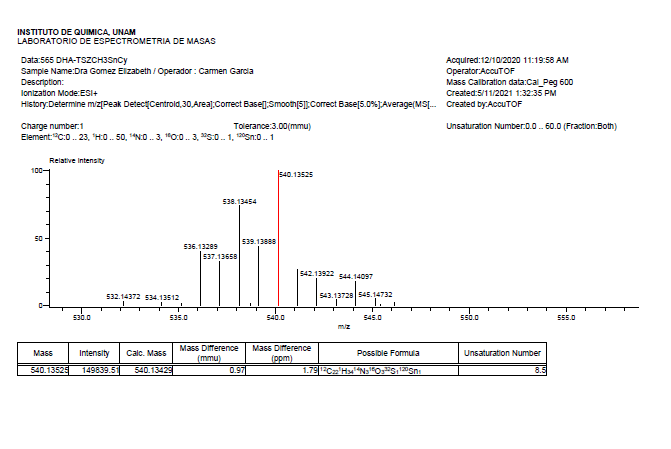


Figure S26. Mass Spectrometry (DART) of compound **2d.**

Figure S27. Mass Spectrometry (EI) of compound **2d.**


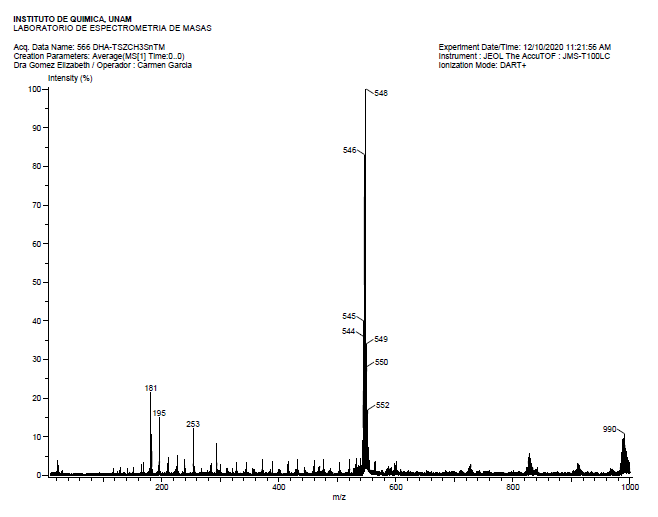


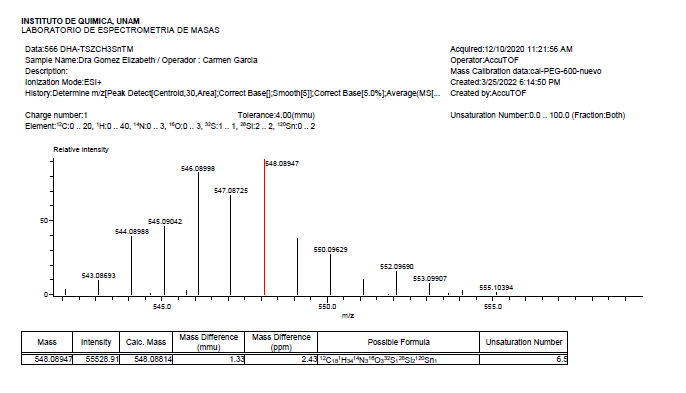


Figure S28. Mass Spectrometry (DART) of compound **2e.**


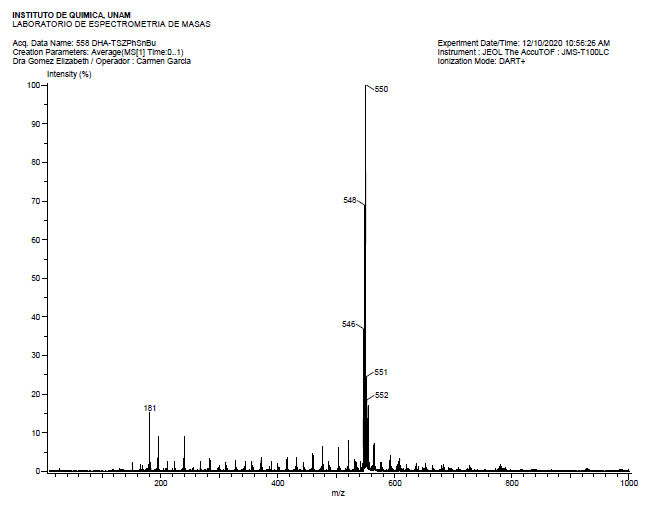


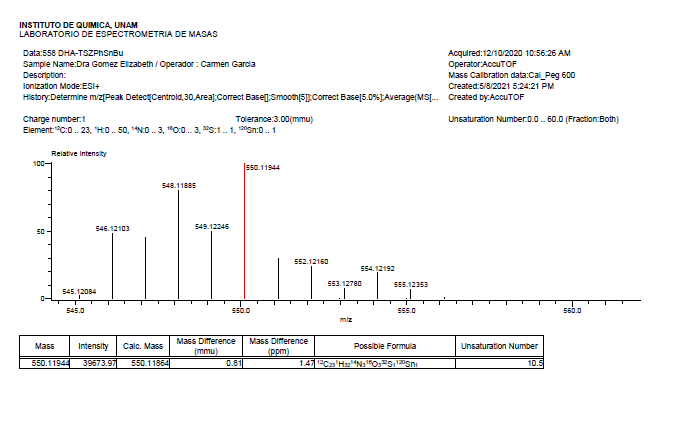


Figure S29. Mass Spectrometry (DART) of compound **3a.**

Figure S30. Mass Spectrometry (EI) of compound **3a.**


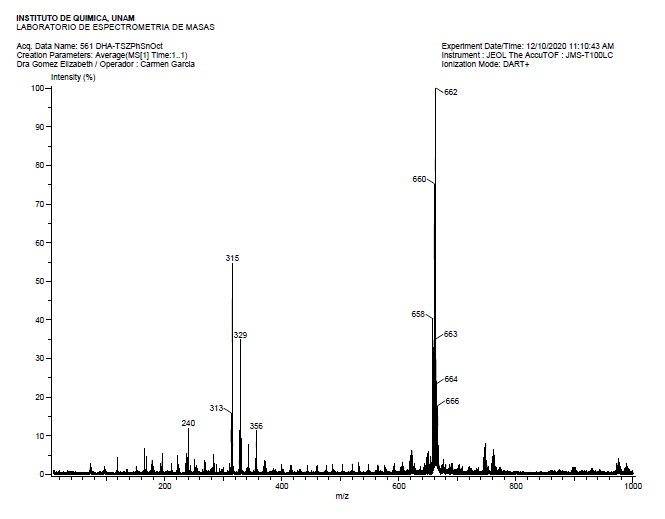


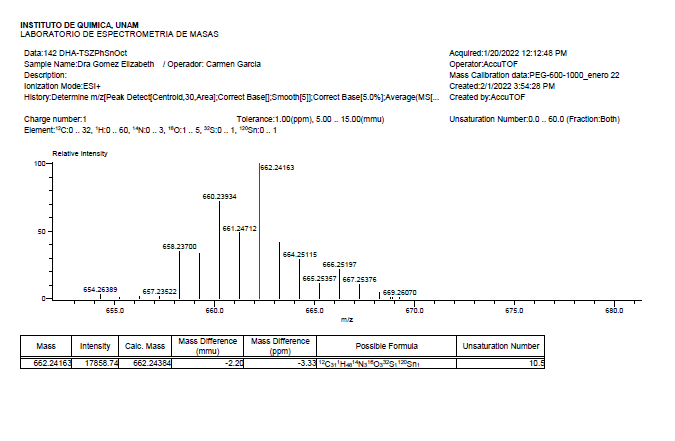


Figure S31. Mass Spectrometry (DART) of compound **3b.**


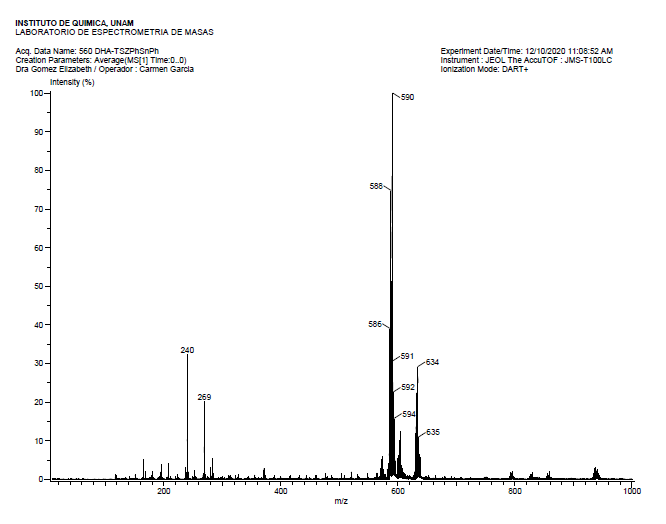


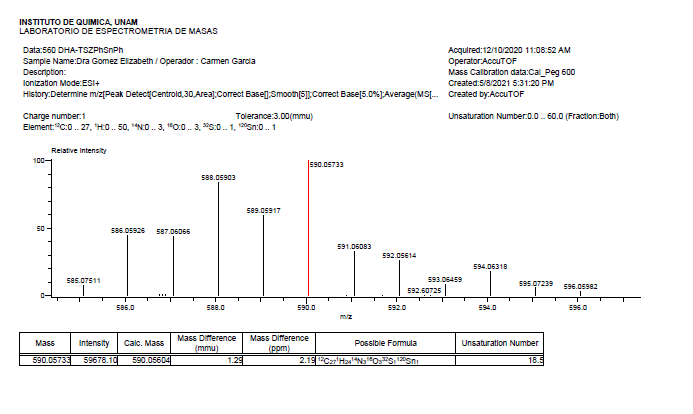


Figure S32. Mass Spectrometry (DART) of compound **3c.**


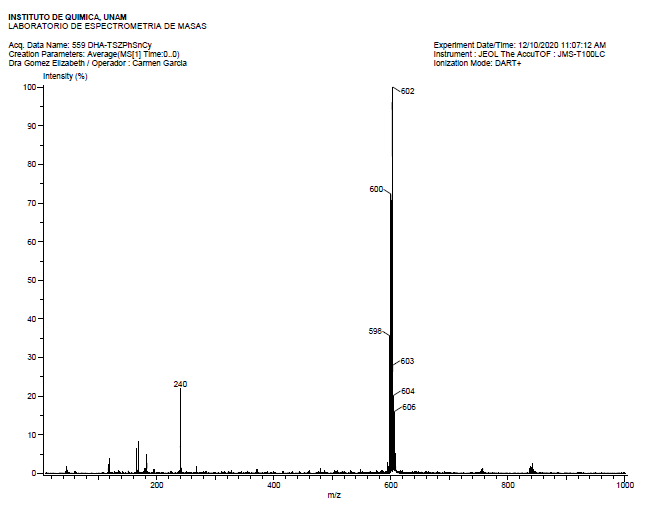


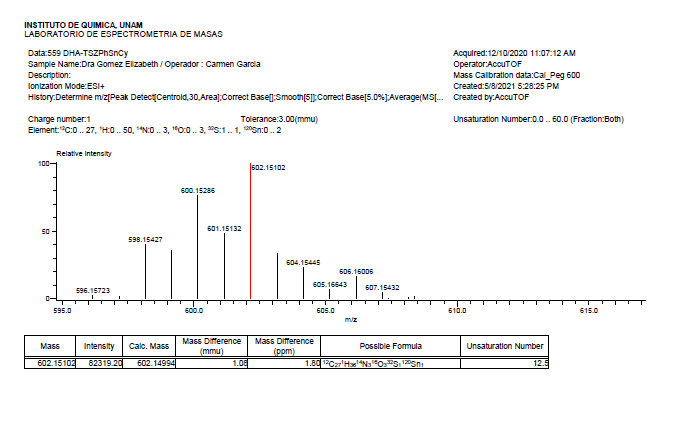


Figure S33. Mass Spectrometry (DART) of compound **3d.**

Figure S34. Mass Spectrometry (EI) of compound **3d.**


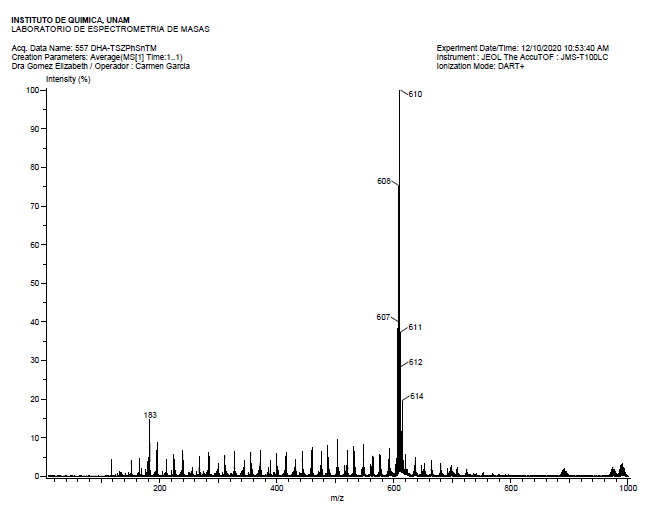


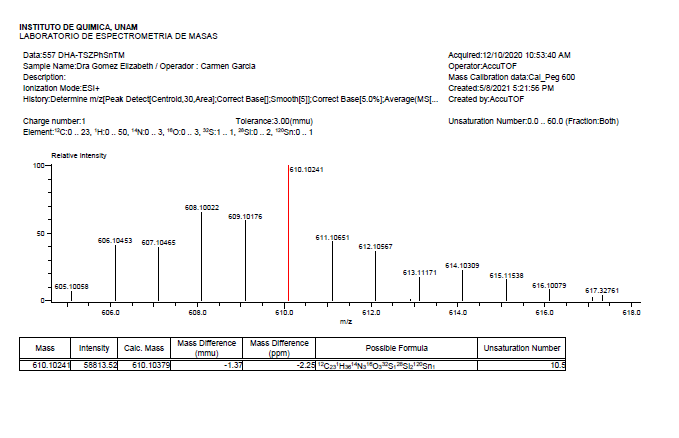


Figure S35. Mass Spectrometry (DART) of compound **3e.**


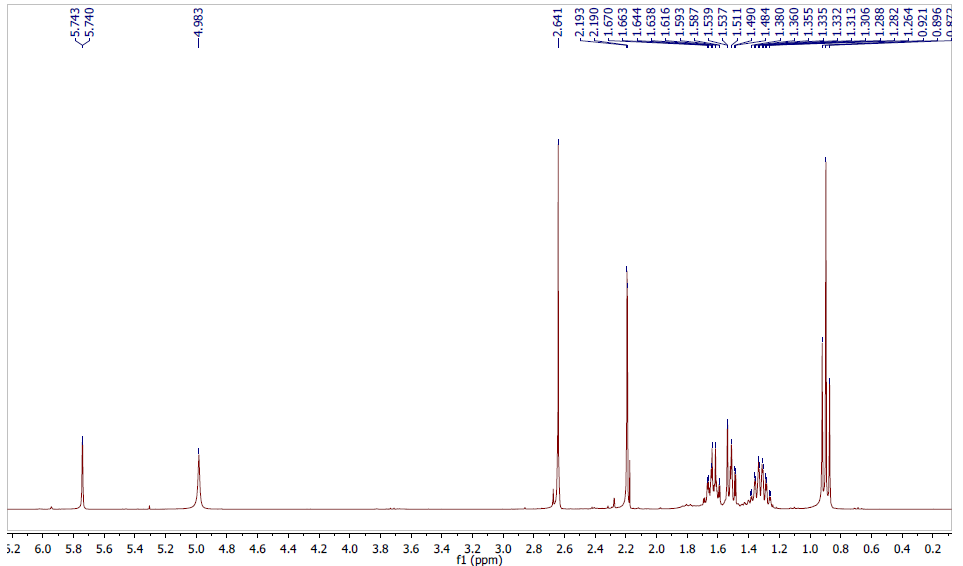


Figure S36. ^1^H NMR (300.52 MHz, CDCl_3_) of compound **1a**.

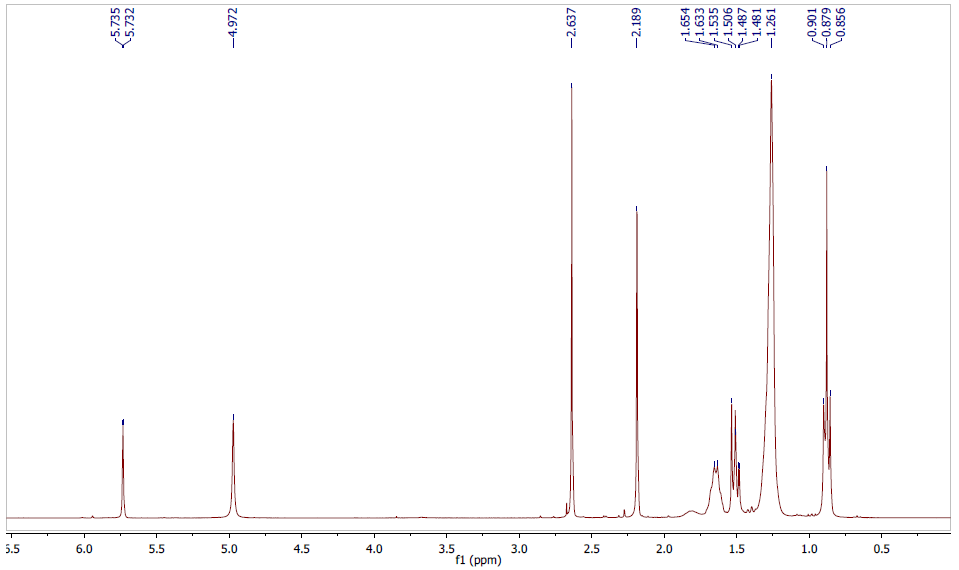


Figure S37. ^1^H NMR (300.52 MHz, CDCl_3_) of compound **1b**.


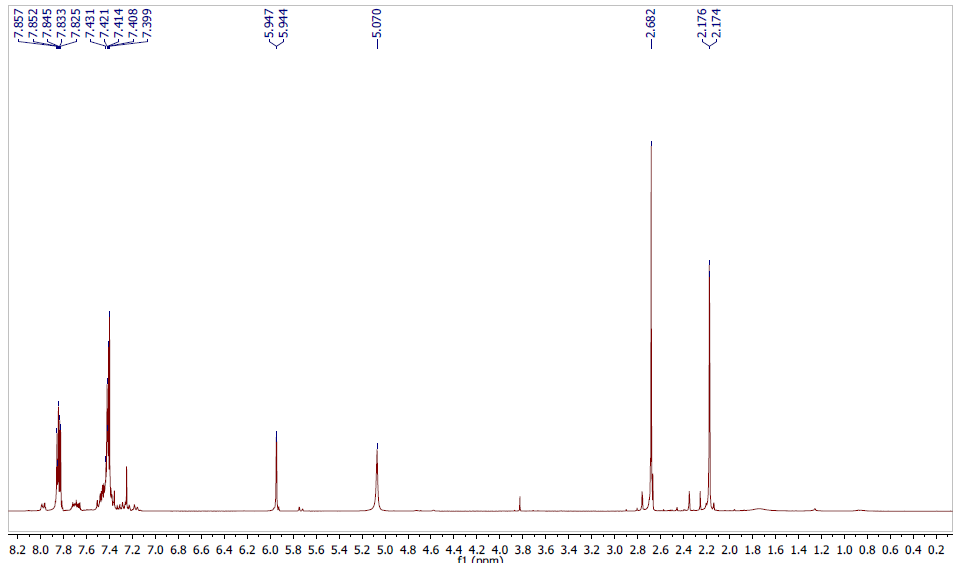


Figure S38. ^1^H NMR (300.52 MHz, CDCl_3_) of compound **1c**.


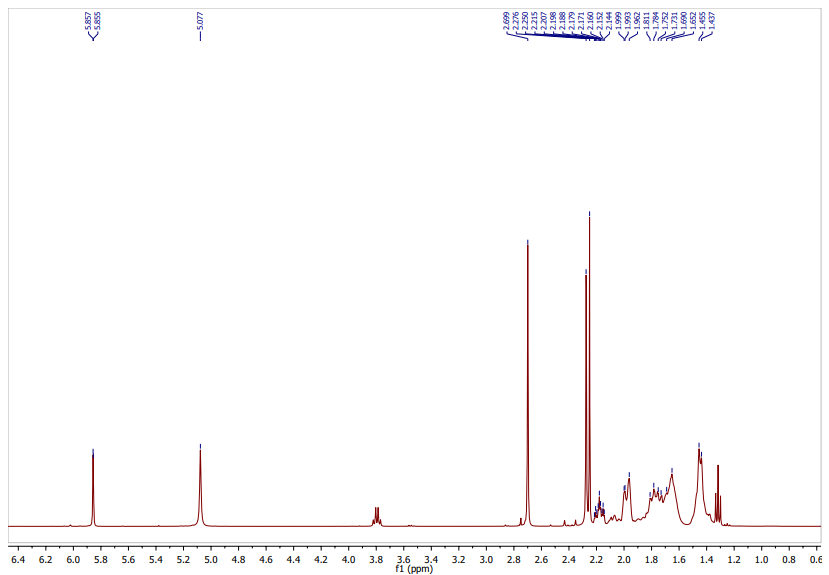


Figure S39. ^1^H NMR (400.13 MHz, CDCl_3_) of compound **1d**.


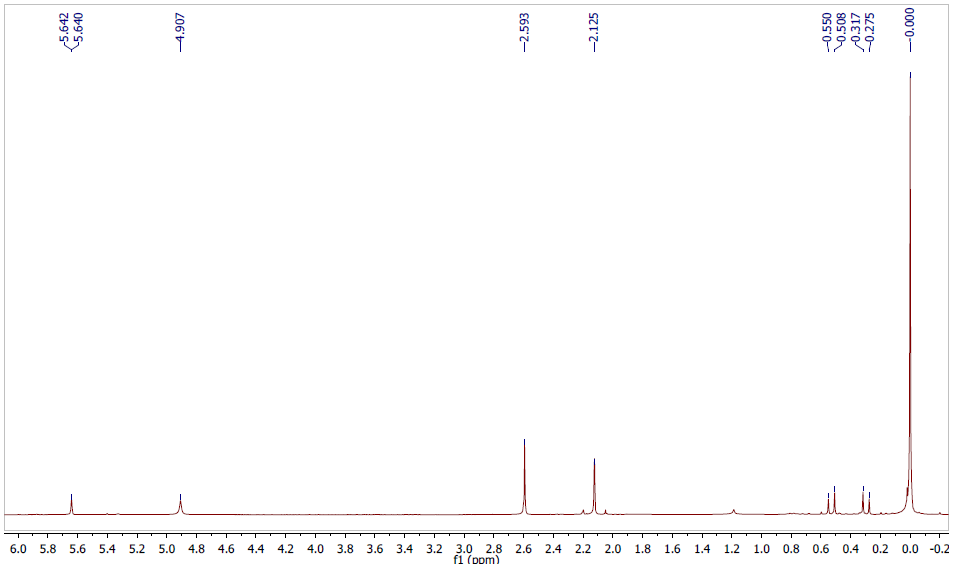


Figure S40. ^1^H NMR (300.52 MHz, CDCl_3_) of compound 1**e**.


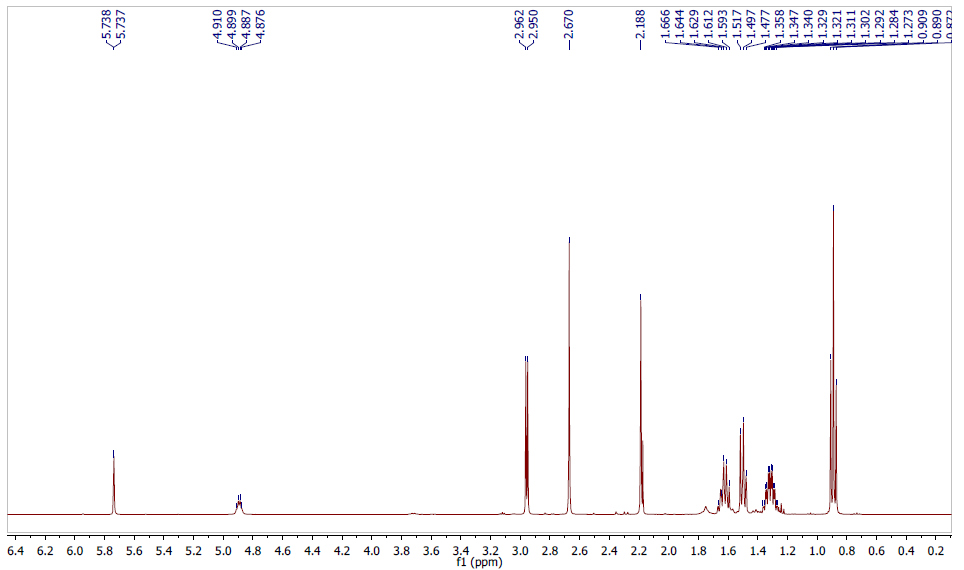


Figure S41. ^1^H NMR (400.13 MHz, CDCl_3_) of compound **2a**.


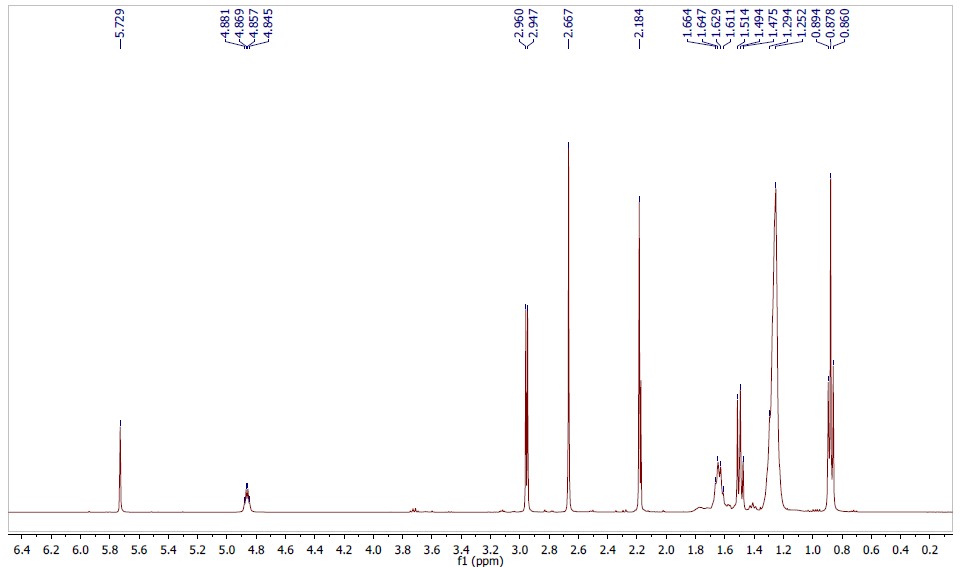


Figure S42. ^1^H NMR (400.13 MHz, CDCl_3_) of compound **2b**.


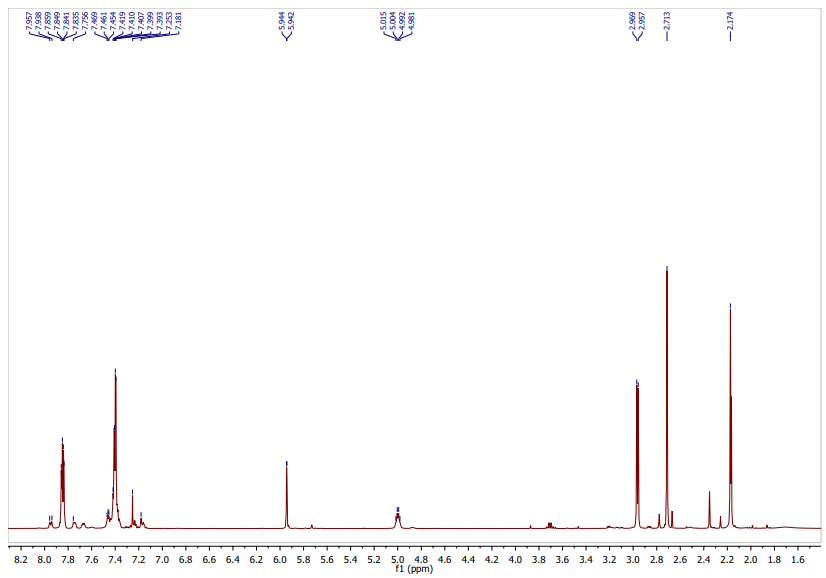


Figure S43. ^1^H NMR (400.13 MHz, CDCl_3_) of compound **2c**.


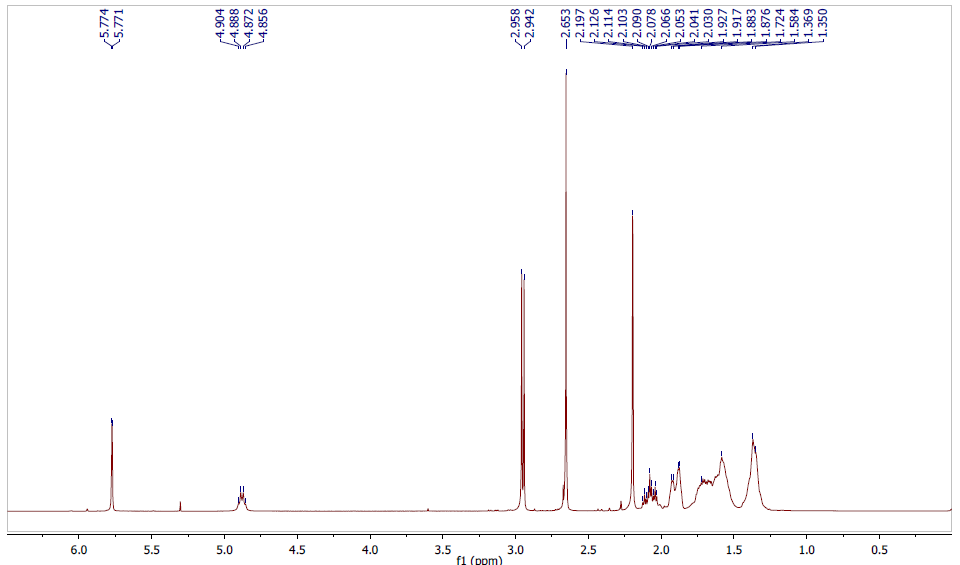


Figure S44. ^1^H NMR (400.13 MHz, CDCl_3_) of compound **2d**.


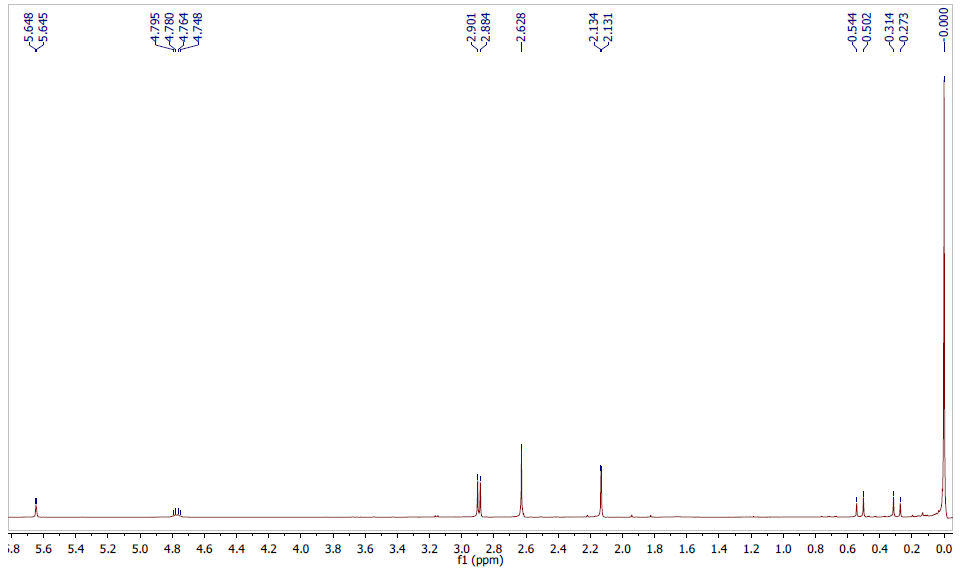


Figure S45. ^1^H NMR (300.52 MHz, CDCl_3_) of compound **2e**.


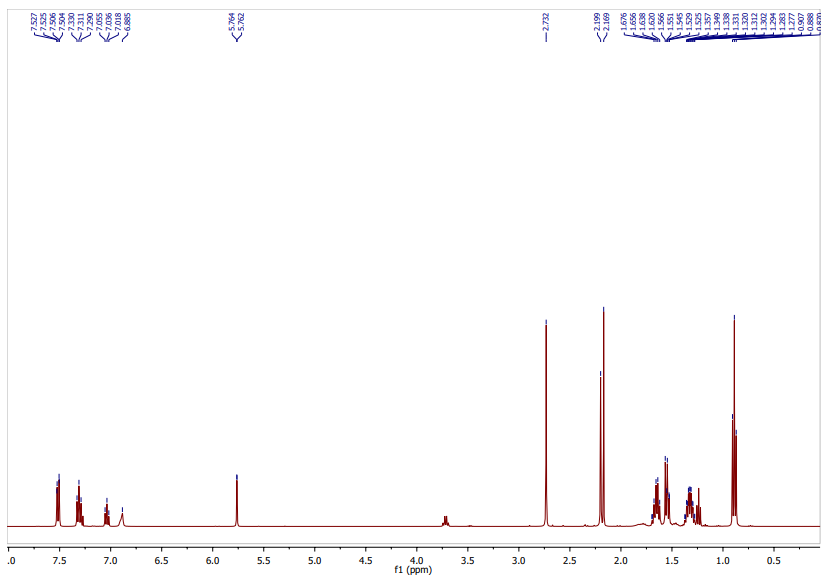


Figure S46. ^1^H NMR (400.13 MHz, CDCl_3_) of compound **3a**.


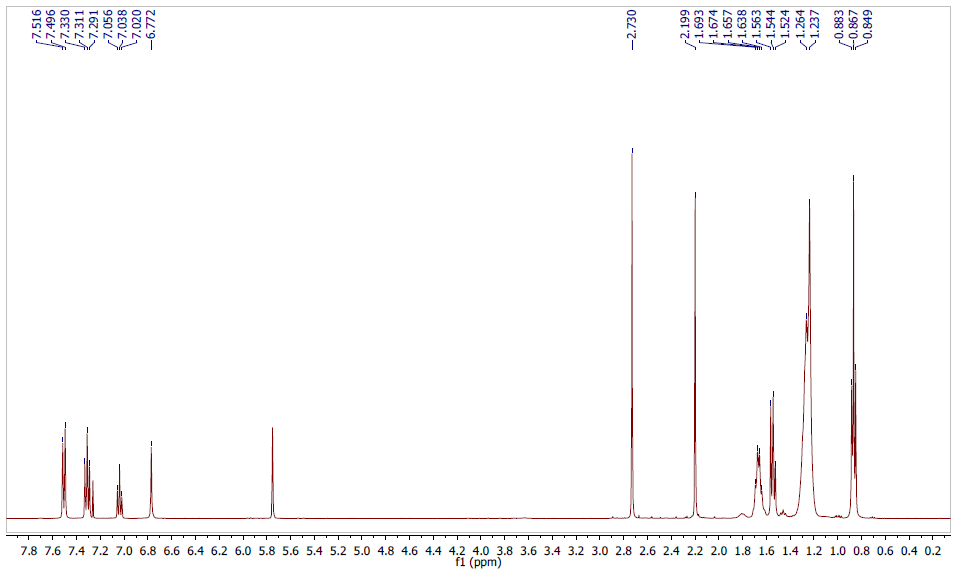


Figure S47. ^1^H NMR (400.13 MHz, CDCl_3_) of compound **3b**.


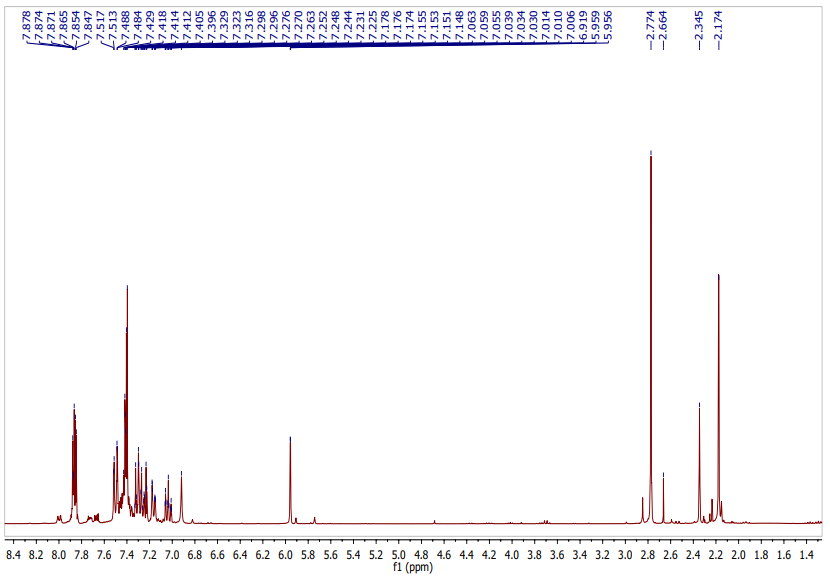


Figure 48. ^1^H NMR (400.13 MHz, CDCl_3_) of compound **3c**.


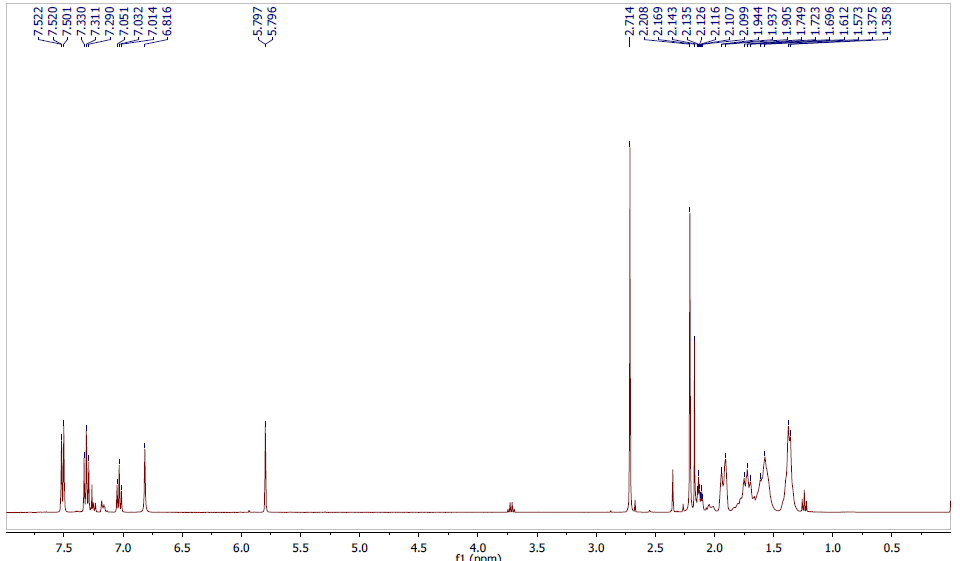


Figure S49. ^1^H NMR (400.13 MHz, CDCl_3_) of compound **3d**.


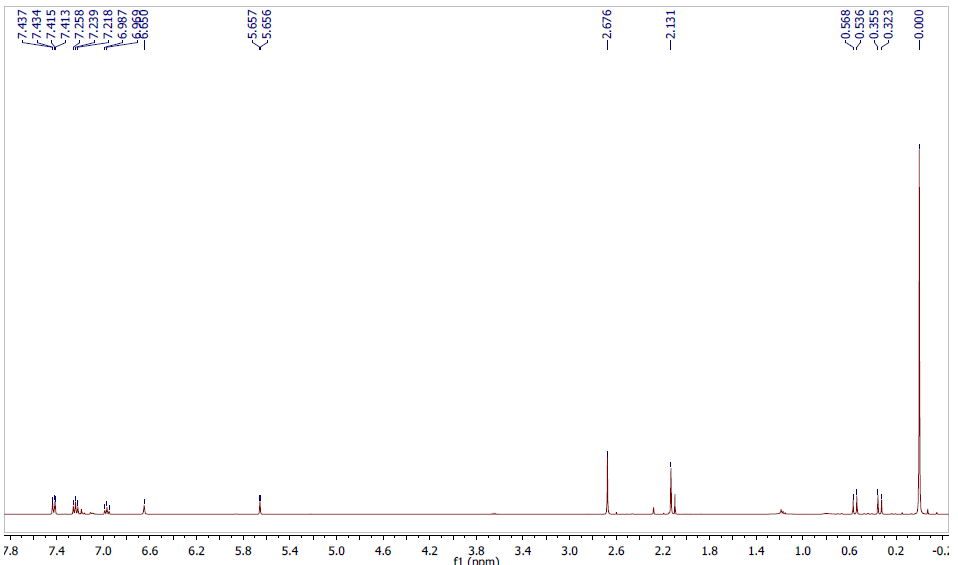


Figure S50. ^1^H NMR (400.13 MHz, CDCl_3_) of compound **3e**


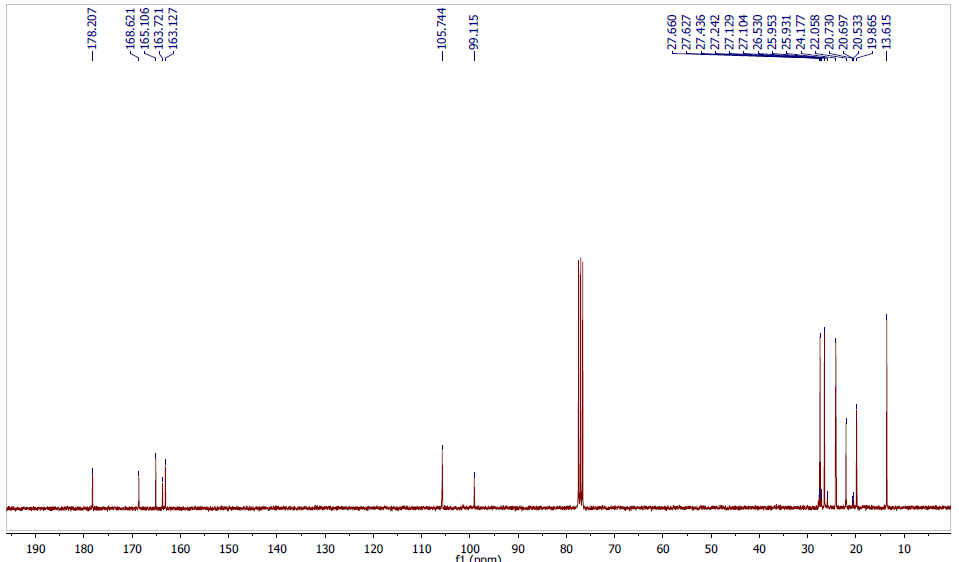


Figure S51. ^13^C NMR (75.57 MHz, CDCl_3_) of compound **1a**.


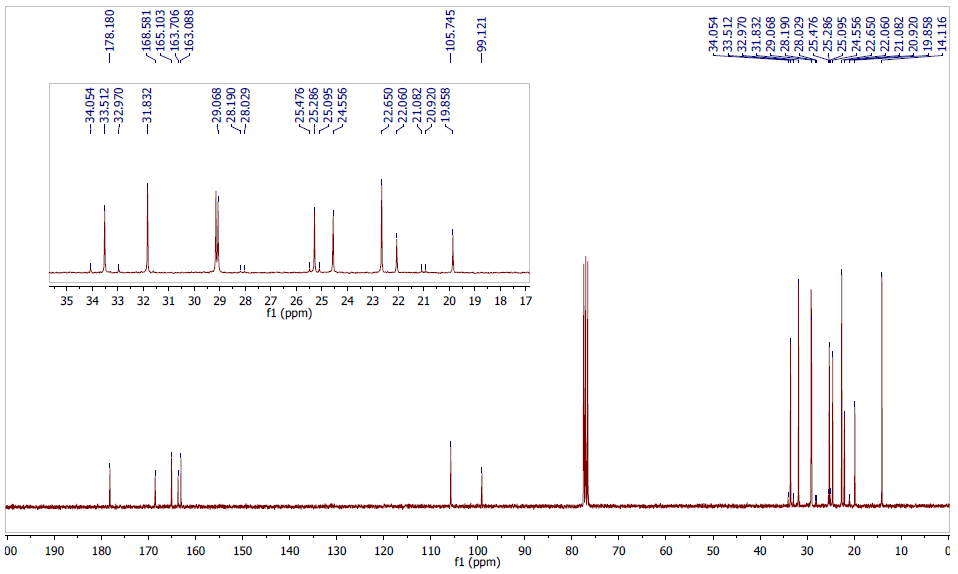


Figure S52. ^13^C NMR (75.57 MHz, CDCl_3_) of compound **1b**.


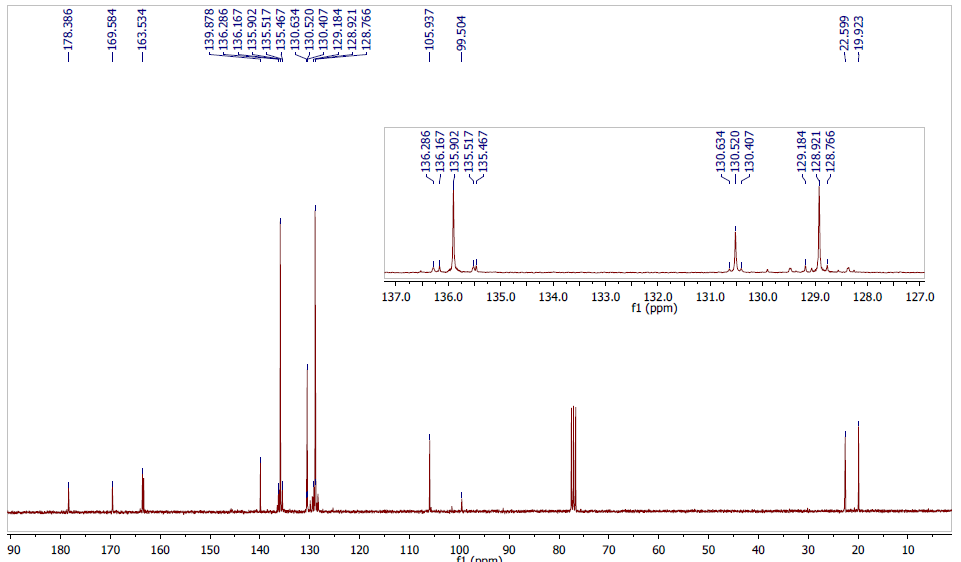


Figure S53. ^13^C NMR (75.57 MHz, CDCl_3_) od compound **1c**.


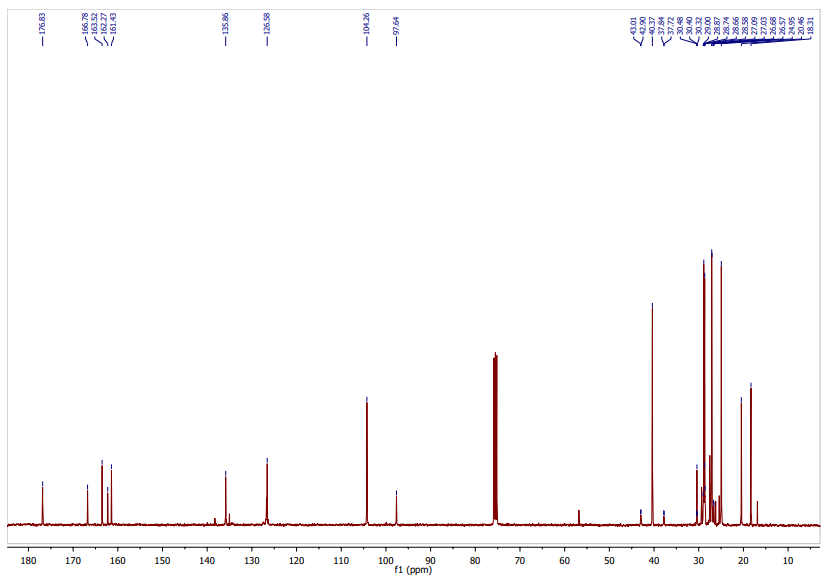


Figure S54. ^13^C NMR (100.62 MHz, CDCl_3_) of compound **1d**.


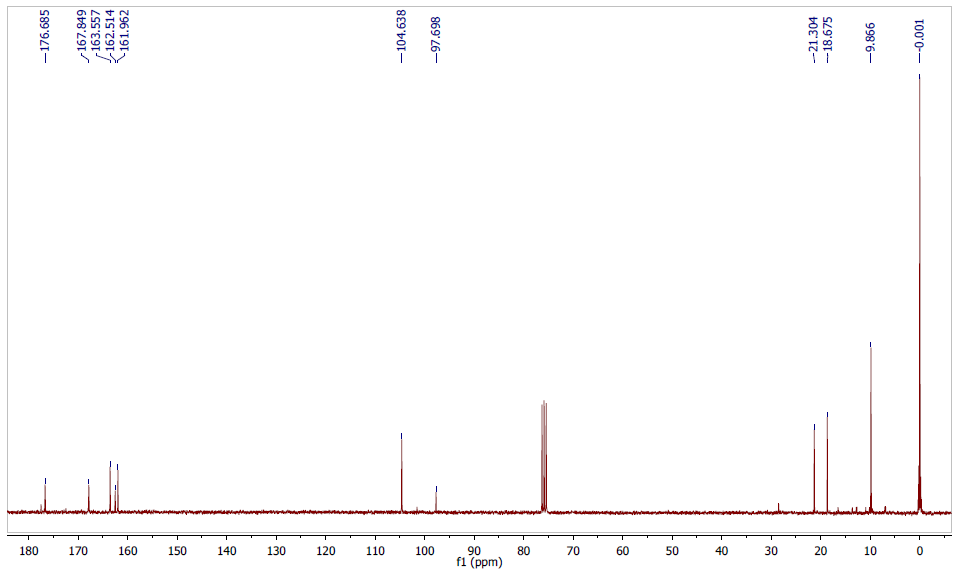


Figure S55. ^13^C NMR (75.57 MHz, CDCl_3_) of compound **1e**.


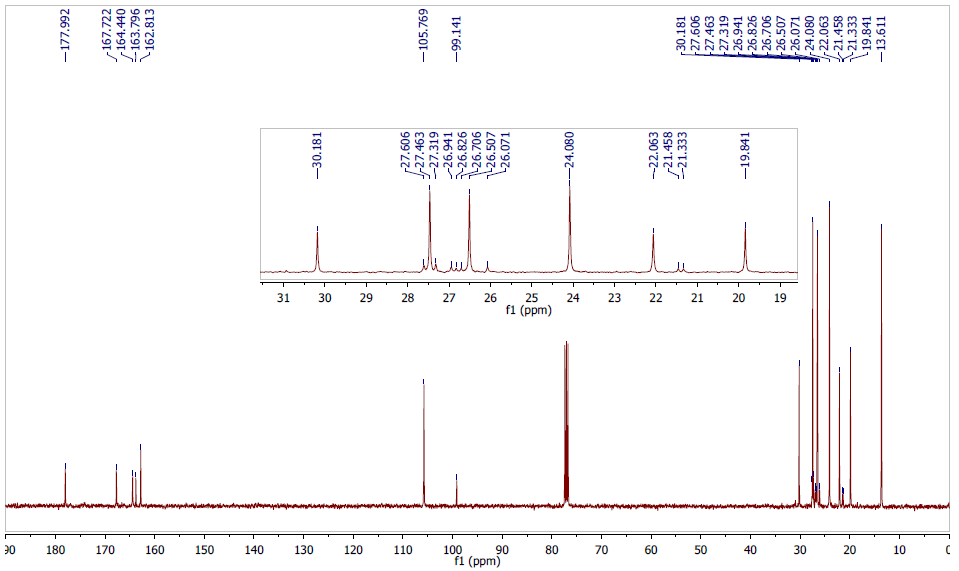


Figure S56. ^13^C NMR (100.62 MHz, CDCl_3_) of compound **2a**.


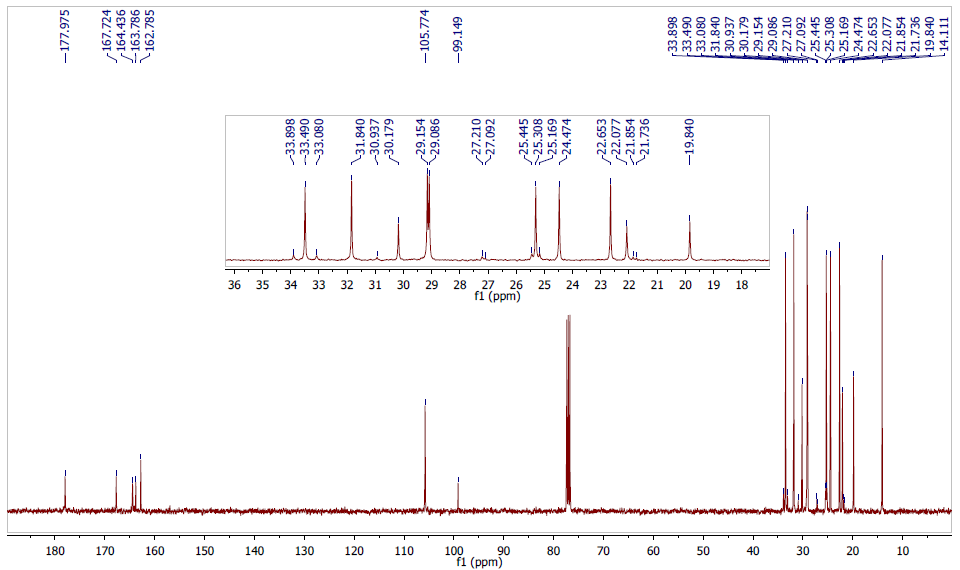


Figure S57. ^13^C NMR (100.62 MHz, CDCl_3_) of compound **2b**.


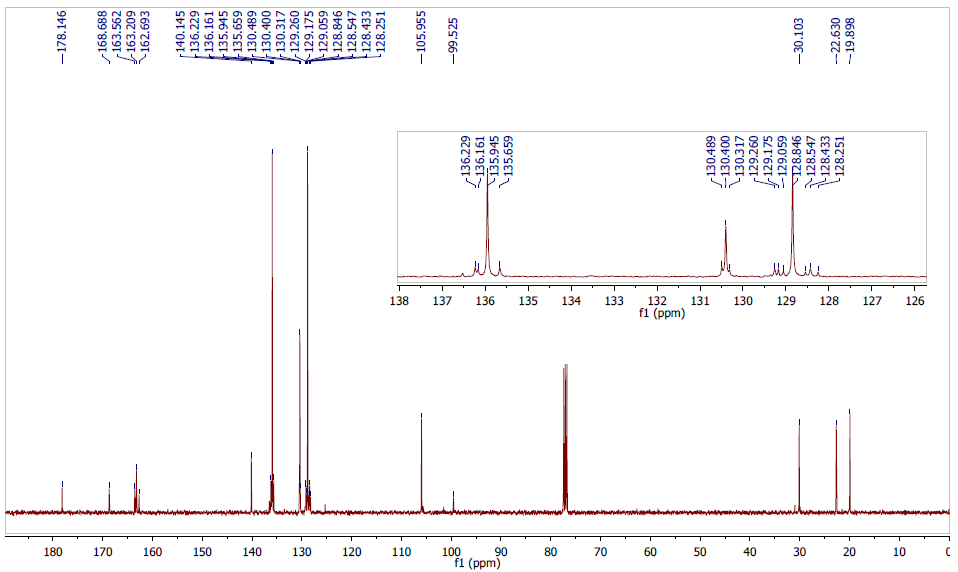


Figure S58. ^13^C NMR (100.62 MHz, CDCl_3_) of compound **2c**.


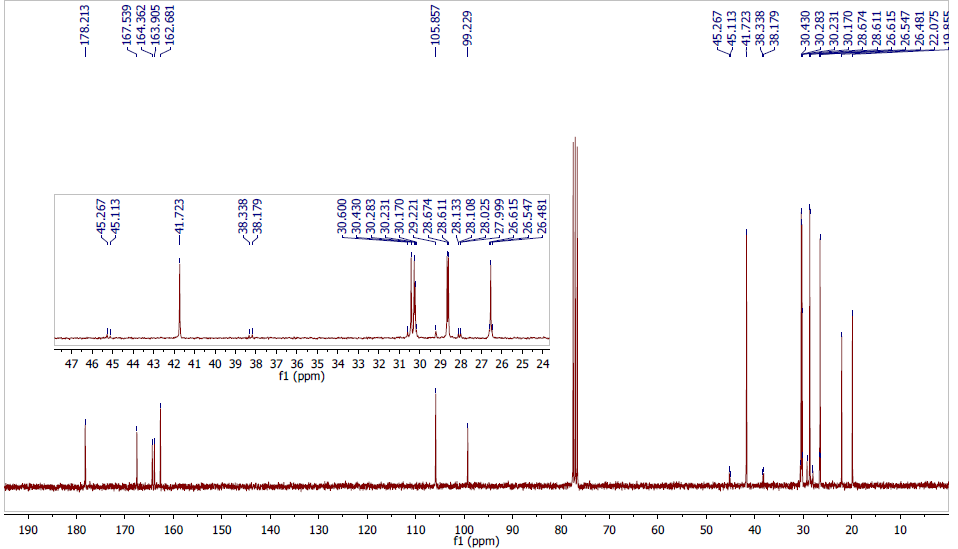


Figure S59. ^13^C NMR (100.62 MHz, CDCl_3_) of compound **2d**.


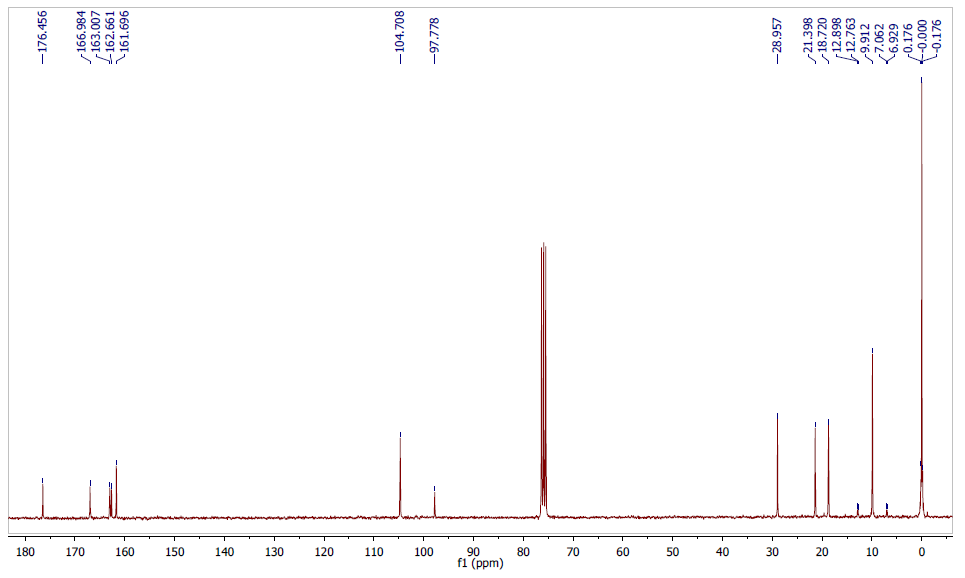


Figure S60. ^13^C NMR (75.57 MHz, CDCl_3_) of compound 2**e**.


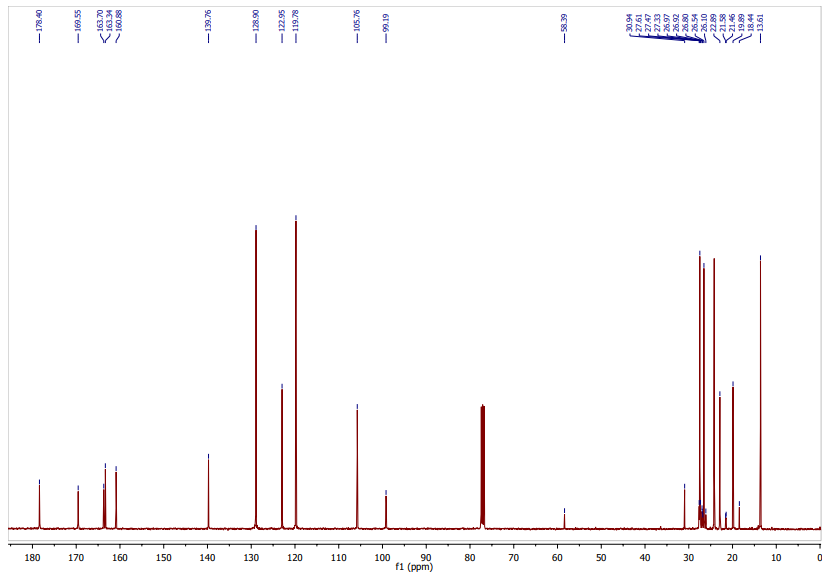


Figure S61. ^13^C NMR (100.62 MHz, CDCl_3_) of compound **3a**.


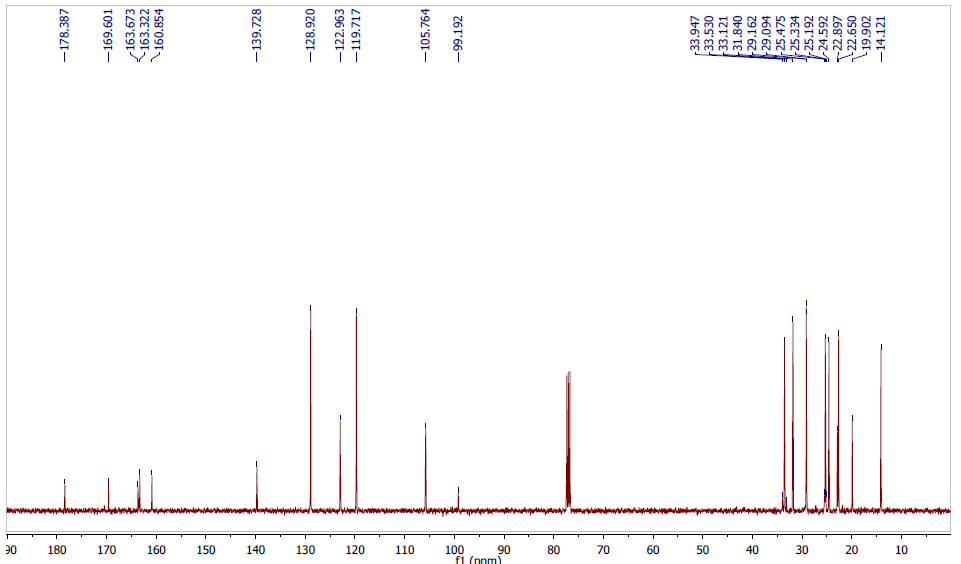


Figure S62. ^13^C NMR (100.62 MHz, CDCl_3_) of compound **3b**.


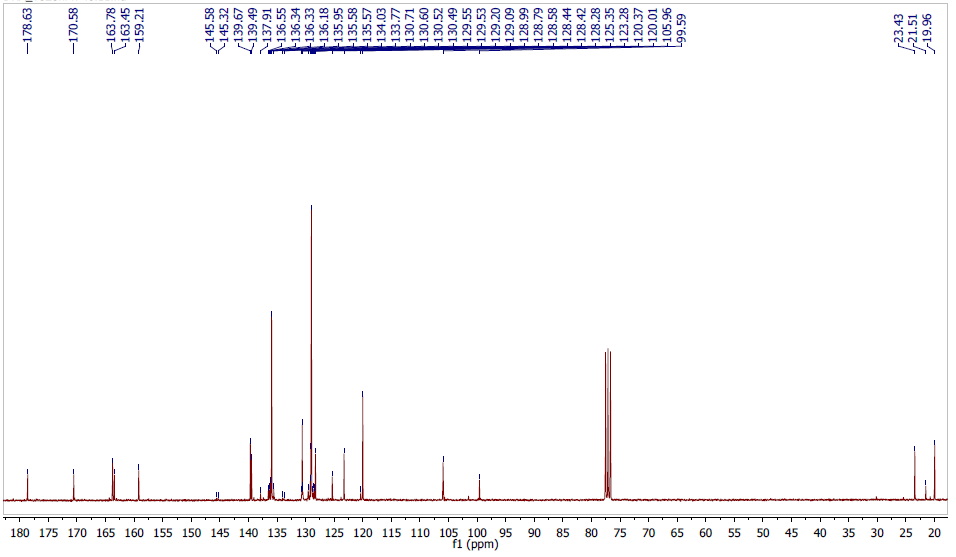


Figure S63. ^13^C NMR (100.62 MHz, CDCl_3_) of compound **3c**.


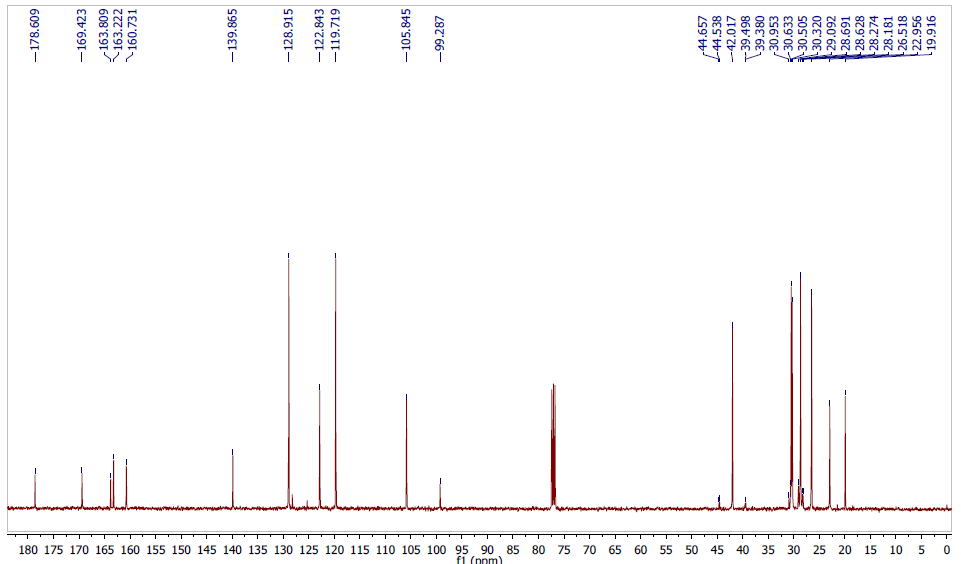


Figure S64. ^13^C NMR (100.62 MHz, CDCl_3_) of compound **3d**.


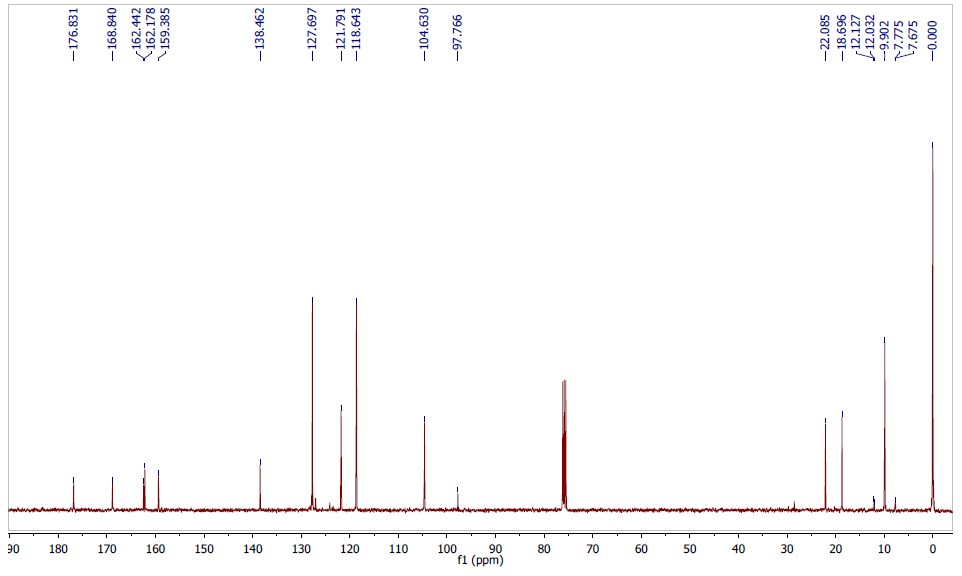


Figure S65. ^13^C NMR (100.62 MHz, CDCl_3_ of compound **3e**.


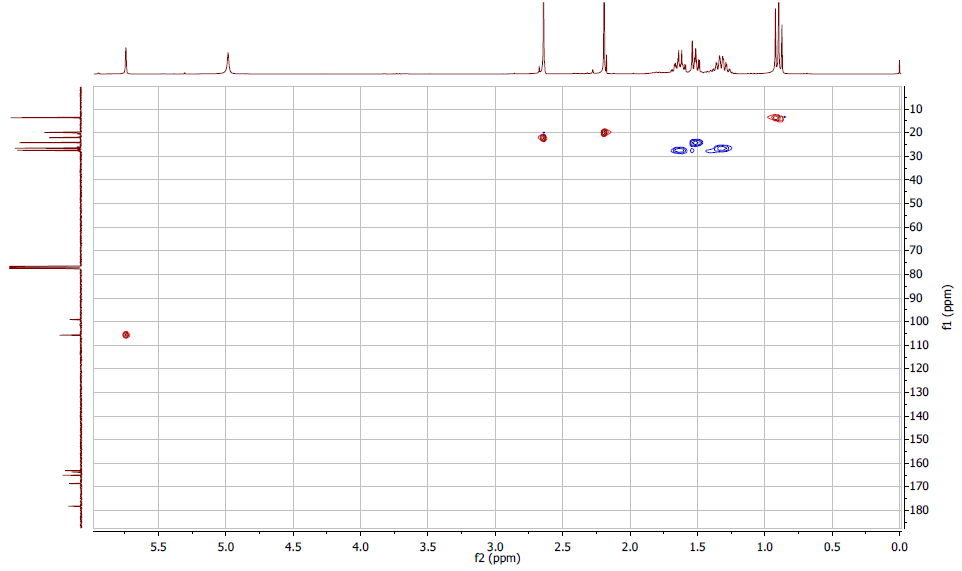


Figure S66. HSQC of compound **1a**.


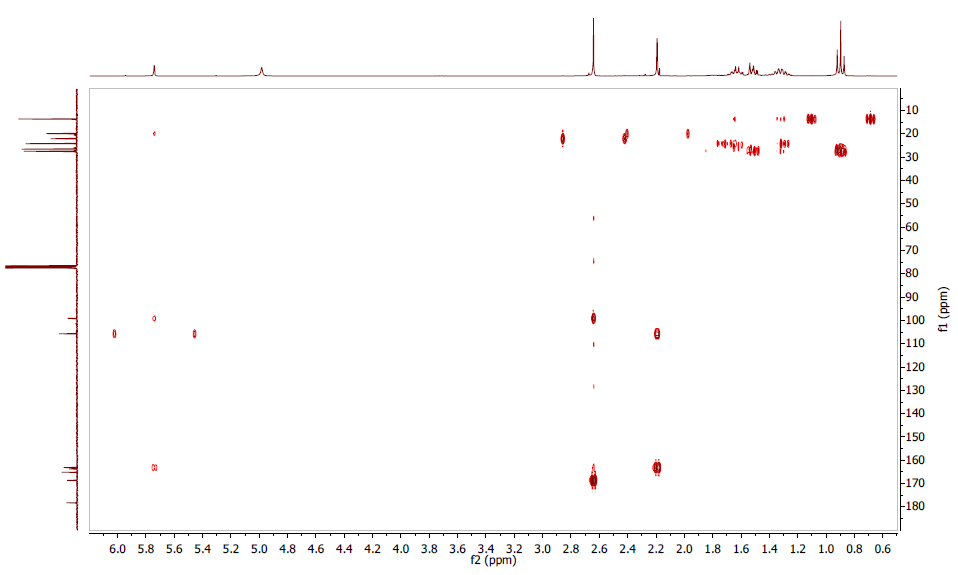


Figure S67. HMBC of compound **1a**.


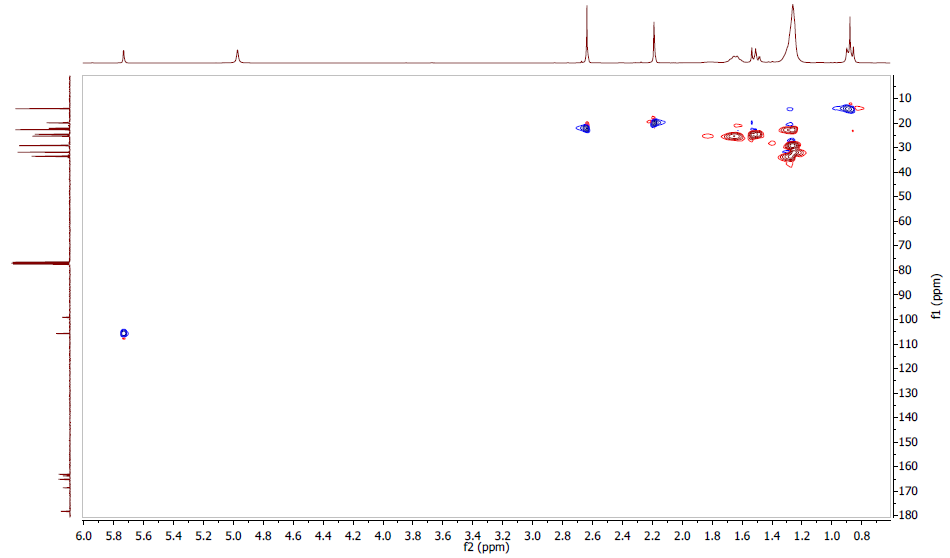


Figure S68. HSQC of compound **1b**.


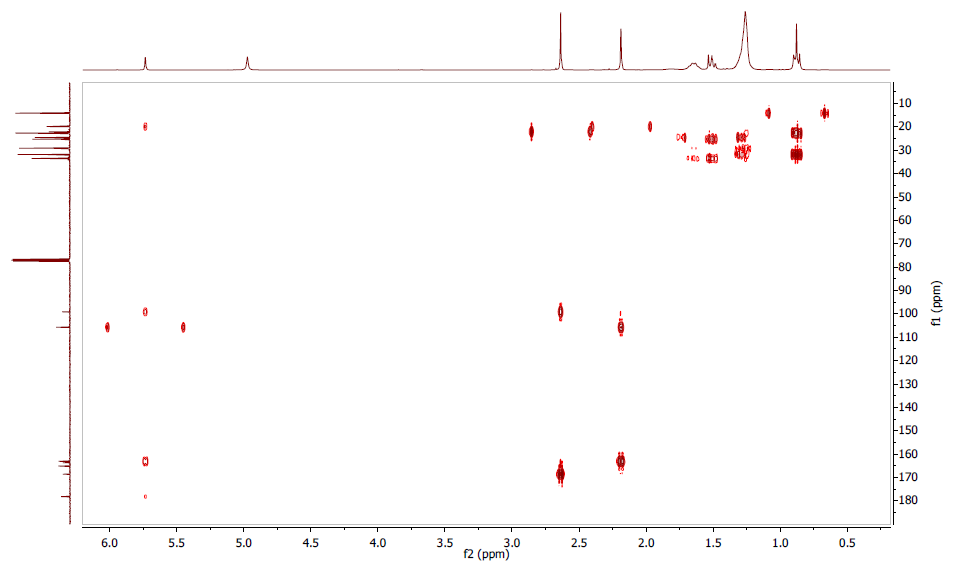


Figure S69. HMBC of compound **1b**.


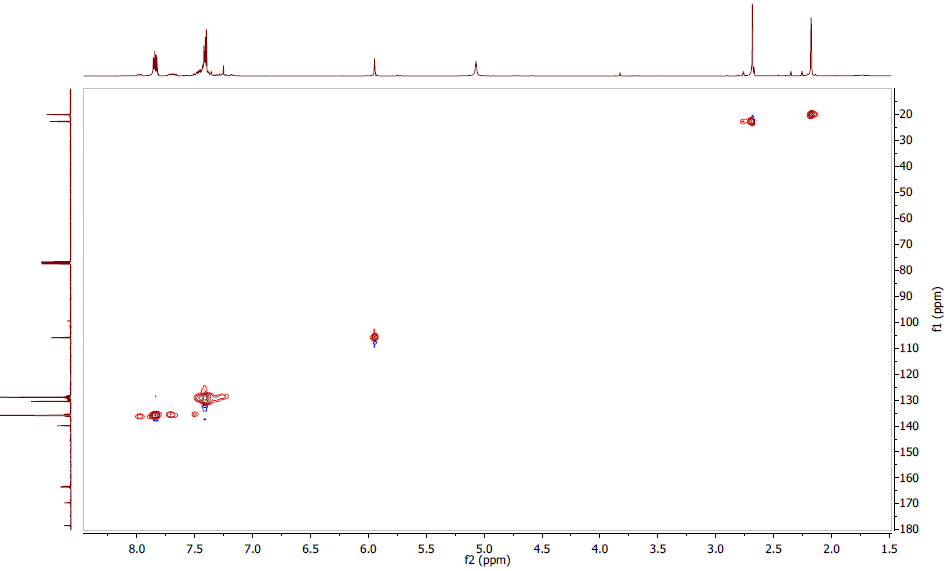


Figure S70. HSQC of compound **1c**.


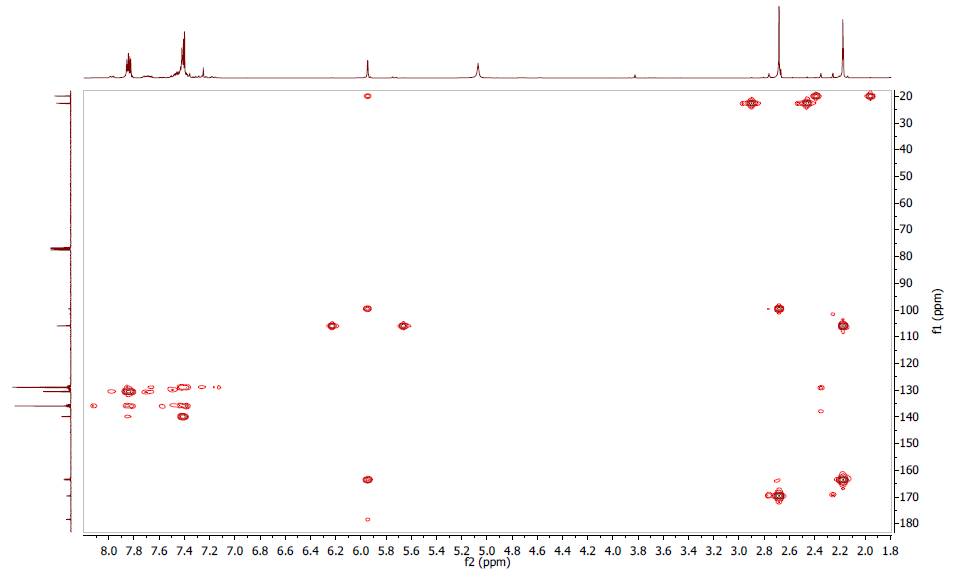


Figure S71. HMBC, of compound **1c.**


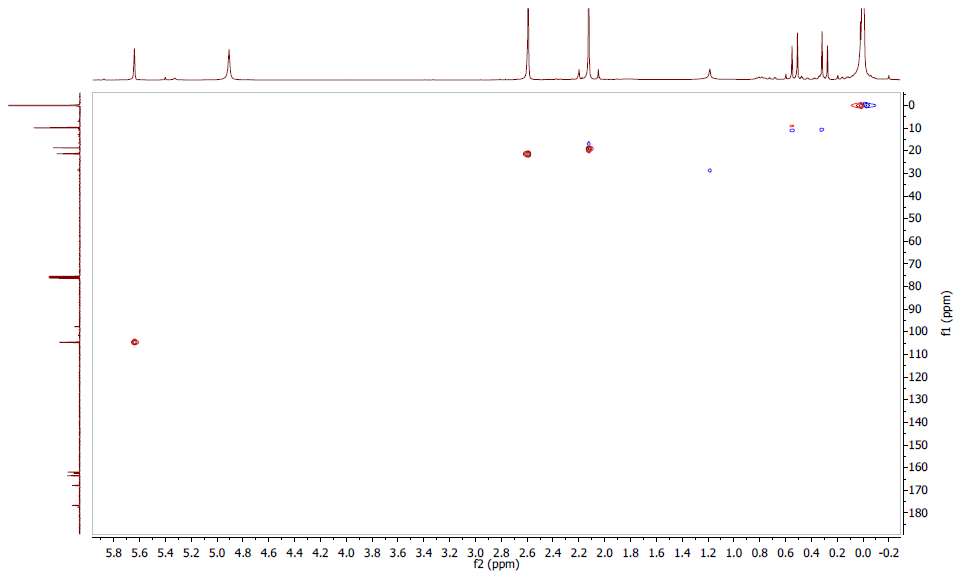


Figure S72. HSQC of compound **1e**.


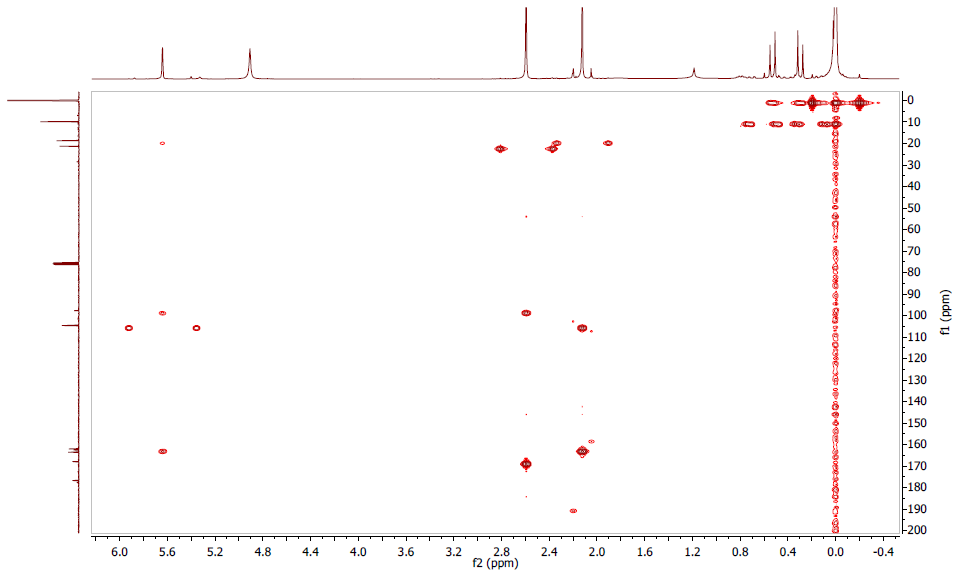


Figure S73. HMBC of compound **1e**.


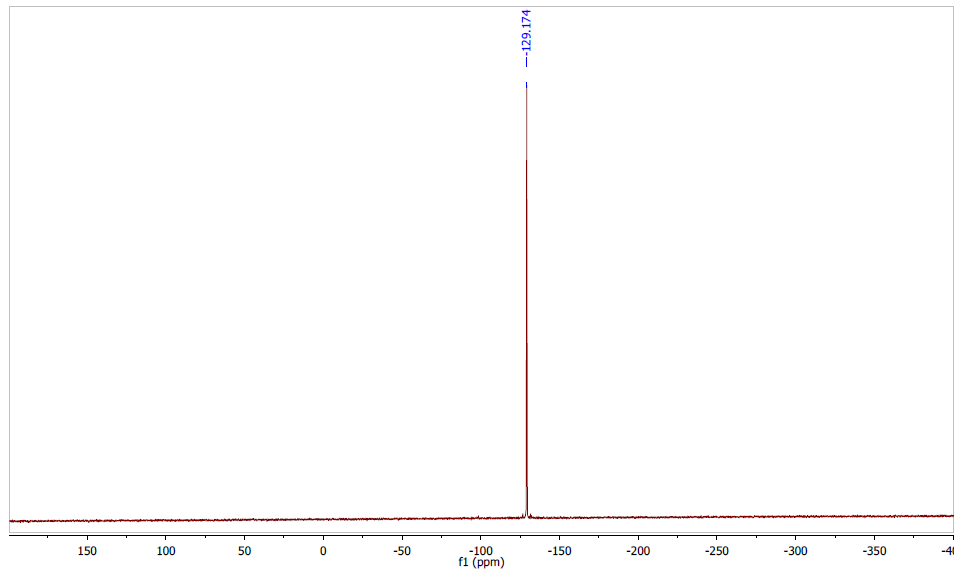


Figure S74. ^119^Sn NMR (112.04 MHz CDCl_3_) of compound **1a**.


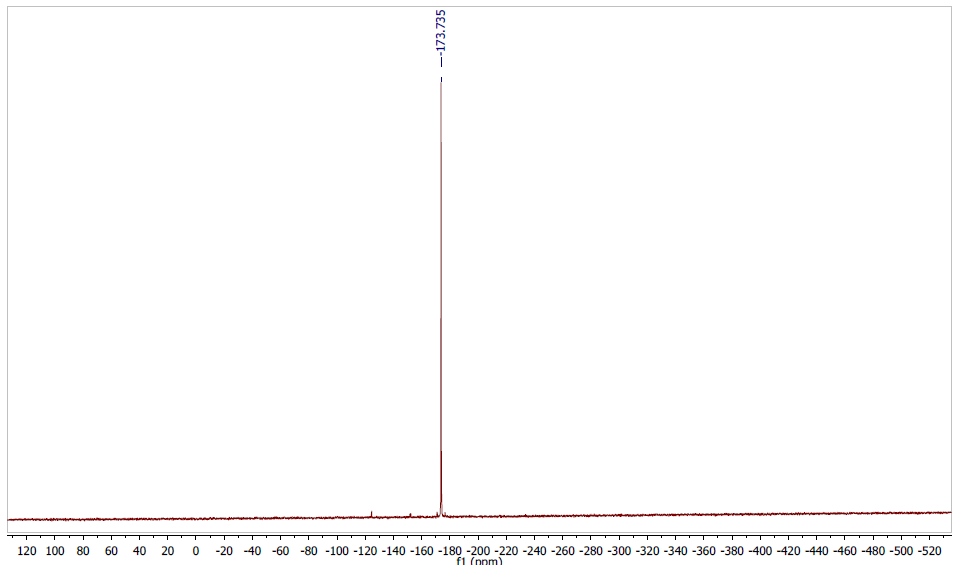


Figure S75. ^119^Sn NMR (112.04 MHz, DMSO-_d6_) of compound **1a**.


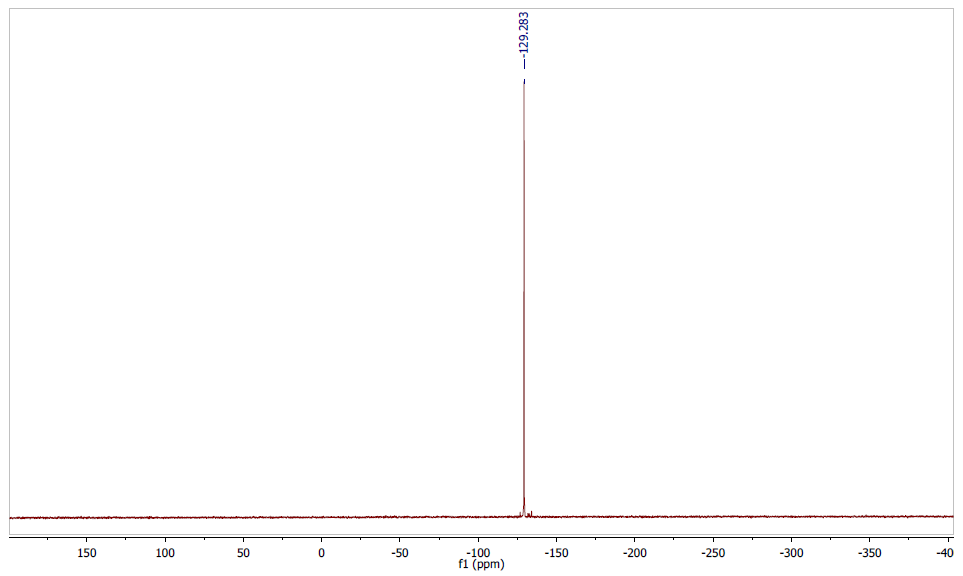


Figure S76. ^119^Sn NMR (112.04 MHz, CDCl_3_) of compound **1b**.

Figure S77. ^119^Sn NMR (112.04 MHz, DMSO-_d6_) of compound **1b**.

Figure S78. ^119^Sn NMR (112.04 MHz, CDCl_3_) of compound **1c**.

Figure S79. ^119^Sn NMR (112.04 MHz, DMSO-_d6_) of compound **1c**.

Figure S80. ^119^Sn NMR (112.04 MHz, CDCl_3_) of compound **1d**.

Figure S81. ^119^Sn NMR (112.04 MHz, DMSO-_d6_) of compound **1d**.

Figure S82. ^119^Sn NMR (112.04 MHz, CDCl_3_) of compound **1e**.

Figure S83. ^119^Sn NMR (112.04 MHz, DMSO_-d6_) of compound **1e**.

Figure S84. ^119^Sn NMR (112.07 MHz, CDCl_3_) of compound **2a**.

Figure S85. ^119^Sn NMR (112.07 MHz, DMSO-_d6_) of compound **2a**.

Figure S86. ^119^Sn NMR (149.21 MHz, CDCl_3_) of compound **2b**.

Figure S87. ^119^Sn NMR (149.21 MHz, DMSO-_d6_) of compound **2b**.

Figure S88. ^119^Sn NMR (149.21 MHz, CDCl_3_) of compound **2c**.

Figure S89. ^119^Sn NMR (149.21 MHz, DMSO-_d6_) of compound **2c**.

Figure S90. ^119^Sn NMR (111.91 MHz, CDCl_3_) of compound **2d**.

Figure 91. ^119^Sn NMR (111.91 MHz, CDCl_3_) of compound **2d**.

Figure S92. ^119^Sn NMR (149.21 MHz, CDCl_3_) of compound **2e**.

Figure S93. ^119^Sn NMR (149.21 MHz, DMSO_-d6_) of compound **2e**.

Figure S94. ^119^Sn NMR (149.21 MHz, CDCl_3_) of compound **3a**.

Figure S95. ^119^Sn NMR (149.21 MHz, DMSO_-d6_) of compound **3a**.

Figure S96. ^119^Sn NMR (149.21 MHz, CDCl_3_) of compound **3b**.

Figure S97. ^119^Sn NMR (149.21 MHz, DMSO_-d6_) of compound **3b**.

Figure S98. ^119^Sn NMR (149.21 MHz, CDCl_3_) of compound **3c**.

Figure S99. ^119^Sn NM NMR (149.21 MHz, DMSO_-d6_) of compound **3c**

Figure S100. ^119^Sn NMR (149.21 MHz, CDCl_3_) of compound **3d**.

Figure S101. ^119^Sn NMR (149.21 MHz, DMSO_-d6_) of compound **3d**.

Figure S102. ^119^Sn NMR (149.21 MHz, CDCl_3_) of compound **3e**.

Figure S103. ^119^Sn NMR (149.21 MHz, DMSO_-d6_) of compound **3e**.

Figure S104. UV-Vis in DMSO-Tris-HCl-NaCl buffer A) 0 h, B) 72 h.

Figure S105. ^1^H NMR (300 MHz, DMSO-d6) of compound **1a** up to 72 h.

Figure S106. ^1^H NMR (300 MHz, DMSO-d6) of compound **1d** up to 72 h.

Figure S107. ^1^H NMR (300 MHz, DMSO-d6) of compound **2a** up to 72 h.

Figure S108. ^1^H NMR (300 MHz, DMSO-d6) of compound **2d** up to 72 h.

Figure S109. ^1^H NMR (300 MHz, DMSO-d6) of compound **3a** up to 72 h.

Figure S110. ^1^H NMR (300 MHz, DMSO-d6) of compound **3d** up to 72 h.

**1a**

**1b**

**1c**

**1d**

**1e**

**2a**

**2b**

**2c**

**2d**

**2e**

**3a**

**3b**

**Figure S111.** Representation of the interactions between A B-DNA dodecamer and diorganotin (IV) derivatives **1a-e**, **2a-e**, **3a-b**. In 3D and 2D models, the interactions and DNA residues are shown (red). In 3D and 2D models, conventional hydrogen bonds (dark green dotted lines), carbon-hydrogen bonds (light green), π-sigma (purple), π-π T-shaped (fuchsia), π-alkyl (pink), and π -anion (orange) are shown.
